# Detailed re-analysis of satellitome mapping facilitated by the telomere-to-telomere (T2T) assembly of bread wheat genome

**DOI:** 10.1007/s11103-026-01745-3

**Published:** 2026-08-28

**Authors:** Manuel A. Garrido-Ramos, Pilar Prieto

**Affiliations:** 1 Departamento de Genética, Facultad de Ciencias, Universidad de Granada, Avda. Fuentenueva s/n, 18071 Granada, Spain; 2 Plant Breeding Department, Institute for Sustainable Agriculture, Agencia Estatal Consejo Superior de Investigaciones Científicas (CSIC), Avda. Menéndez Pidal, Campus Alameda del Obispo s/n, 14004 Córdoba, Spain

**Keywords:** Bread wheat, Satellite DNA, Satellitome, Transposable elements, Recombination, Centromeres, Subtelomeres, Telomeres

## Abstract

The satellitome of *Triticum aestivum* cv. Chinese Spring consists of 36 satellite DNA (satDNA) families that were previously molecularly characterized and chromosomally mapped. The recent release of a complete gap-free telomere-to-telomere (T2T) assembly of the species offers a unique opportunity to refine the physical map and evolutionary interpretation of these sequences. Here, we re-evaluate the organization of the wheat satellitome by analyzing the T2T assembly with RepeatMasker and visualizing the results using the CHRISMAPP script. This research strengthens the notion that more than half of wheat satDNAs are connected to transposable elements (TEs) and enables us to propose a model of satDNA origin and evolution driven by TEs and other random genome sequences. By integrating T2T based physical mapping with previous FISH analyses, we refine the genomic distribution of tandem repeats, identify complex satellite organizations and length variants, and resolve several discrepancies between cytogenetic and genomic analyses. Finally, the detailed mapping of satDNA in centromeric and the subtelomeric regions highlight clear chromosome and subgenome specific patterns, suggesting potential roles of satellites in chromosome architecture. Together these findings provide the most complete and accurate satellitome map available for bread wheat, offering new insights into repeat evolution and genome organization in polyploid species.

**Supplementary Information:**

The online version contains supplementary material available at https://doi.org/10.1007/s11103-026-01745-3.

## Key message

The organization of the different satellite DNA families that constitutes its satellitome is re-evaluated in light of the information provided by the new T2T assembly of the bread wheat genome, revealing relevant information about their origin and evolution, distribution, and role in centromeric and subtelomeric regions.

**Supplementary Information:**

The online version contains supplementary material available at https://doi.org/10.1007/s11103-026-01745-3.

## Introduction

*Triticum aestivum*, bread wheat, is a hexaploid species (2*n* = 6*x* = 42) whose genome is composed of three subgenomes (AABBDD) derived from 3 different ancestral species, reflecting the hybridization and polyploidization processes that have shaped its evolutionary history. Its origin dates back ~ 9000 years to the spontaneous hybridization between *T. turgidum* (AABB; *2n* = *4x* = 28) and *Aegilops tauschii* (DD; *2n* = *2x* = 14) (McFadden and Sears 1946; Dvorak et al. 1998; Petersen et al. 2006; Matsuoka 2011; Brenchley et al. 2012; Deng et al. 2013; Wang et al. 2013). The AABB genome itself likely originated from polyploidization of hybrids between *T. urartu* (AA; *2n* = *2x* = 14) and another closely related to *Ae. speltoides* (BB; *2n* = *2x* = 14) (Dvořák et al. 1993; Feldman et al. 2023; Baum and Grant Bailey 2004; Petersen et al. 2006; Levy and Feldman 2022; Li et al. 2022).

Bread wheat has long been a species of enormous scientific and agronomic interest, not only because of its complex evolutionary origins but also because understanding its genetics and genomics structure is crucial in everything related to its cultivation and crop improvement and global food security. Hence, there has been enormous interest in the complete sequencing of its genome. However, the wheat genome poses major challenges for sequencing and assembly. It contains three sets of homeologous chromosomes, extremely large genome size, high concentration of repetitive sequences (> 85% of the genome), as well as the great diversity of cultivated wheat varieties. Among the ~ 20 accessions sequenced (reviewed in Liu et al. 2025), the sequencing of the genome of the Chinese Spring (CS) variety by the International Wheat Genome Sequencing Consortium (IWGSC) stands out as one of the greatest efforts to complete the first reference-quality genome assembly for the Chinese Spring (CS) variety (RefSeq v.1.0) (IWGSC 2018) which was updated to CS RefSeq (v.2.1) in 2021 (Zhu et al. 2021). Even so, this assembly retained 183,603 gaps, particularly in regions enriched in repetitive DNA, especially tandem repetitive DNA or satellite DNA (satDNA) (Zhu et al. 2021; Liu et al. 2025).

A large portion of the eukaryotic genome is comprised of the repeatome (Kim et al. 2014), which refers to different kinds of repetitive DNA. This genetic material is broadly divided into two groups based on its genomic organization (López-Flores and Garrido-Ramos 2012): dispersed repeats (which spread out across the genome) and tandem repeats (which bunch together in rows). In addition to a few dispersedly repeated gene families, the dispersed category is overwhelmingly dominated by transposable elements (TEs), which typically constitute the vast majority of all repetitive sequences (López-Flores and Garrido-Ramos 2012; Biscotti et al., 2015). Tandemly repetitive DNA encompasses tandemly repeated gene families, telomeric DNA, and various types of genomic sequence repeats such as microsatellites, minisatellites, and satDNA (López-Flores and Garrido-Ramos 2012). Tandem repeats are broadly classified by monomer size into microsatellites (2–10 bp) —also known as Simple Sequence Repeats (SSRs) and Short Tandem Repeats (STRs)—, minisatellites (11–60 bp), and satDNAs (> 60 bp, with monomers > 500 bp sometimes termed macrosatellites) (Garrido-Ramos 2017, 2021; Garrido-Ramos et al. 2025). Due to their high polymorphism, individual variations within short arrays of micro- and minisatellites (collectively termed variable number of tandem repeats or VNTRs) serve as valuable genetic markers in population genetics and forensics. Classically, mini- and microsatellites were thought to primarily reside in euchromatin while satDNAs occupied heterochromatin. However, modern genomics has rendered these rigid size- and location-based boundaries obsolete (Garrido-Ramos 2017, 2021; Garrido-Ramos et al. 2025). For researchers investigating satDNA, the advent of novel genomic tools has opened the door to exploring the satellitome —the complete repertoire of satDNA families within a genome (Ruiz-Ruano et al. 2016)— uncovering an unprecedented diversity level of repeat unit sequence, repeat unit length, and organization (Garrido-Ramos 2017, 2021; Šatović-Vukšić and Plohl 2023; Garrido-Ramos et al. 2025). Beyond the classical view of satDNA as abundant, heterochromatic blocks, high-throughput approaches have revealed that satellitomes also comprise short arrays scattered throughout the genome, with both short and long arrays occurring independently of heterochromatin. These dispersed arrays can act as evolutionary “seeds” capable of amplifying into longer arrays. Consequently, satDNA genomic organization and abundance vary drastically both within a genome and between species (Garrido-Ramos 2017, 2021; Camacho et al. 2022; Šatović-Vukšić and Plohl 2023; Garrido-Ramos et al. 2025) Ultimately, this structural diversity reflects different evolutionary stages of tandem repeat dynamics, often driven by the amplification of other repetitive elements like TEs (Šatović-Vukšić and Plohl 2023; Šatović-Vukšić et al. 2025; Garrido-Ramos et al. 2025).

Using computational tools specially designed for the detection of repetitive DNA from short DNA reads (Novák et al. 2010, 2013, 2017, 2020; Ruiz-Ruano et al. 2016), we were able to present the bread wheat satellitome, including the molecular definition of all satDNA families of the species and their chromosomal distribution by FISH (Gálvez‑Galván et al. 2024a). A comparable satellitome characterization was later achieved for durum wheat (*T. turgidum*) (Gálvez‑Galván et al. 2024b), providing an evolutionary framework for understanding satDNA dynamics in polyploid wheat. A major leap forward came with the recent publication of the telomere‑to‑telomere (T2T), gap‑free CS‑IAAS assembly (Liu et al. 2025), which spans 14.51 Gb and includes all 21 centromeres and 42 telomeres with unprecedented completeness and accuracy. This resource finally allows the full physical mapping of tandem repeats across the wheat genome, overcoming the limitations of previous assemblies where highly repetitive regions were fragmented or collapsed. In this context, we have reevaluated in this work the bread wheat satellitome using the T2T assembly combined with additional bioinformatics tools. This approach has resulted in more detailed and comprehensive information on the bread wheat satellitome. By integrating T2T‑based mapping with previous approaches, we refine the distribution of repetitive sequences, identify previously undetected arrays and complex organizational patterns, and resolve inconsistencies found in earlier cytogenetic interpretations enabling us to develop a model of wheat satDNAs evolution. In addition, we gained insights into genome architecture of tandem repeats and the role of satellites in centromeric and subtelomeric regions in genome organization. Together, these advances offer the most complete assessment to date of the wheat satellitome and provide essential insights into the role of tandem repeats in the structural and functional evolution of polyploid genomes.

## Materials and methods

### Satellitome mapping in the T2T assembly of the bread wheat genome

We analyze here the genome mapping of the bread wheat satellitome obtained in a previous work (Gálvez-Galván et al. 2024a). For the satellitome identification, we used the satMiner protocol (Ruiz-Ruano et al. 2016), which recursively uses RepeatExplorer2 (Novák et al. 2020), a pipeline that uses graph-based clustering to search, identify, and characterize novel satellites from NGS data (Novák et al. 2010, 2013) and TAREAN (Novák et al. 2020), integrated in RepeatExplorer2, which performs automated identification of satDNA repeats based on the topology of their cluster graphs (circular shape in the case of satDNAs) and generates a consensus monomer sequence for each satDNA family. Based on this operational criterion, a satDNA family is the set of sequencing reads that share a sufficiently high sequence identity (90% similarity over at least 55% of the read length) to be grouped into the same three-dimensional cluster and that share a common basic repeating unit (monomer). Each biological family identified by the program must be capable of collapsing into a single consensus sequence (a single representative monomer). satDNA families were named using a standard, four-part formula proposed by Ruiz-Ruano et al (2016). The naming structure is formatted as [Species Abbreviation] + Sat + [Catalog Number] + [Monomer Length]; therefore, the name of each satellite contains important information, such as the length of its consensus monomer. Then, we generated a database composed of 34 satDNA families isolated by us as well as 2 additional families identified in Kapustová et al. (2019), totaling the 36 satDNA families comprising the bread wheat satellitome analyzed in Gálvez-Galván et al. (2024a). Thirteen of the families analyzed matched all known satDNA families previously isolated as pAs1, Afa, TaiI, PSc119.2, CentT566, CentT550, etc. (see Table S1 in Gálvez-Galván et al. 2024a).

The T2T assembly of the bread wheat genome cv. CS was downloaded from https://doi.org/10.5281/zenodo.10716954 (Liu et al. 2025). The assembly was then analyzed locally with RepeatMasker (Smit et al. 2015) using our custom database. The data tables generated were analyzed manually and the resulting.gff file, containing comprehensive annotations, underwent processing in RStudio (R Core Team 2024), using the ggplot2 package (Wickham 2016) using the R script CHRISMAPP_V2.1.R (Rico-Porras et al. 2024) to visualize the annotations, with some modifications and additions so that the script can recognize the.gff files generated by RepeatMasker and so that the script identifies and draws the centromeres (see the Appendix 1 in Supplementary Information).

With these data we defined organizational patterns of wheat satDNAs. Given the wide range of variation in monomer length among the 36 satellites analyzed (44–2619 bp), we believe that the most objective criterion for defining an array is the number of repeat units (RUs) per array. In this context, we considered a long array to be one with a number of RUs greater than or equal to 1000. Structural pattern A includes both long and short arrays (fewer than 1000 RUs), while structural pattern B includes only short arrays. Among the short arrays, we highlighted those that do not exceed 10 bp (referred to here as "very short arrays") for two main reasons. First, they define a model: numerous copies of each satellite, regardless of whether they belong to Pattern A or B, are scattered throughout the genome (either isolated or forming arrays of a few RUs) and serve as the building blocks for future, new longer arrays. These very short arrays have also been shown to play a role in the regulation of gene expression (Garrido-Ramos et al. 2025). Second, they define a structural pattern (structural pattern C) that is interesting from an evolutionary perspective; they correspond to dispersed repetitive elements linked to TEs that occasionally undergo duplication, giving rise to mini-arrays of fewer than 10 RUs, thus constituting TE-derived tandem repeats, which could serve as the foundation for future arrays of new satDNAs, although their long-term success remains uncertain. A custom R script (see the Appendix 2 in Supplementary Information) was used to organize the data and generate statistical summaries and comparative figures by patterns and satDNA families. Statistical analyses and data visualization were performed using R within the RStudio integrated development environment (R Core Team 2024). Box plots were generated using the ggplot2 (Wickham 2016) and patchwork (Pedersen 2024) packages, both integrated within the tidyverse ecosystem (Wickham et al. 2019), after data import via readxl (Wickham and Bryan 2023).

As additional tools for analyzing the arrays of each satDNA identified by RepeatMasker in the assembly, we used the Tandem Repeat Finder (TRF) program (Benson 1999). Also, we searched Repbase (Bao et al. 2015) for satDNAs homologies with transposable elements using CENSOR (Kohany et al. 2006), with the “no_low” and “no_is” options, using “Triticum” as the sequence source.

### Mapping of satDNAs in centromeric and subtelomeric regions

We extracted the sequences corresponding to the centromeres of each chromosome using bedtools (Quinlan and Hall 2010):

$ bedtools getfasta [options] -fi assembly.fasta -bed Centromeres.bed.

Where “Centromeres” is a file in bed format with 21 lines (one for each chromosome) and three columns: a) chromosome number and subgenome to which it belongs; b) centromere start site on each chromosome; and c) centromere end site on each chromosome, according to data provided by Liu et al. (2025). Whereas the “assembly” file in fasta format is the T2T assembly of Liu et al. (2025).

The chromosomes of bread wheat are very long (average length 691 Mb, with extreme values between 505 and 874 Mb), so the graphic image generated by CHRISMAPP is highly compressed and it is difficult to obtain a detailed physical map of a specific region rich in satDNA; for example, the ends of the chromosomes. For this reason, we created a loop in the R script that generates chromosome graphs by windows of a specific length, 30 Mb for example (about 5% of the average length of the species' chromosomes), so that we can analyze each chromosomal region in detail from one end to the other, window by window. This also gives us a more accurate view of what is happening in the most distal regions of the chromosomes. Since each chromosome of the species has a different length, we included instructions to generate a graph that aligns the last 30 Mb of each chromosome (the end of the long arm of each chromosome). These additions to the CHRISMAPP script are described in the Annex in Supplementary Information. The development of the code has been assisted by Perplexity AI.

## Results and discussion

### Organizational patterns of wheat satDNAs

**Fig. 1:**
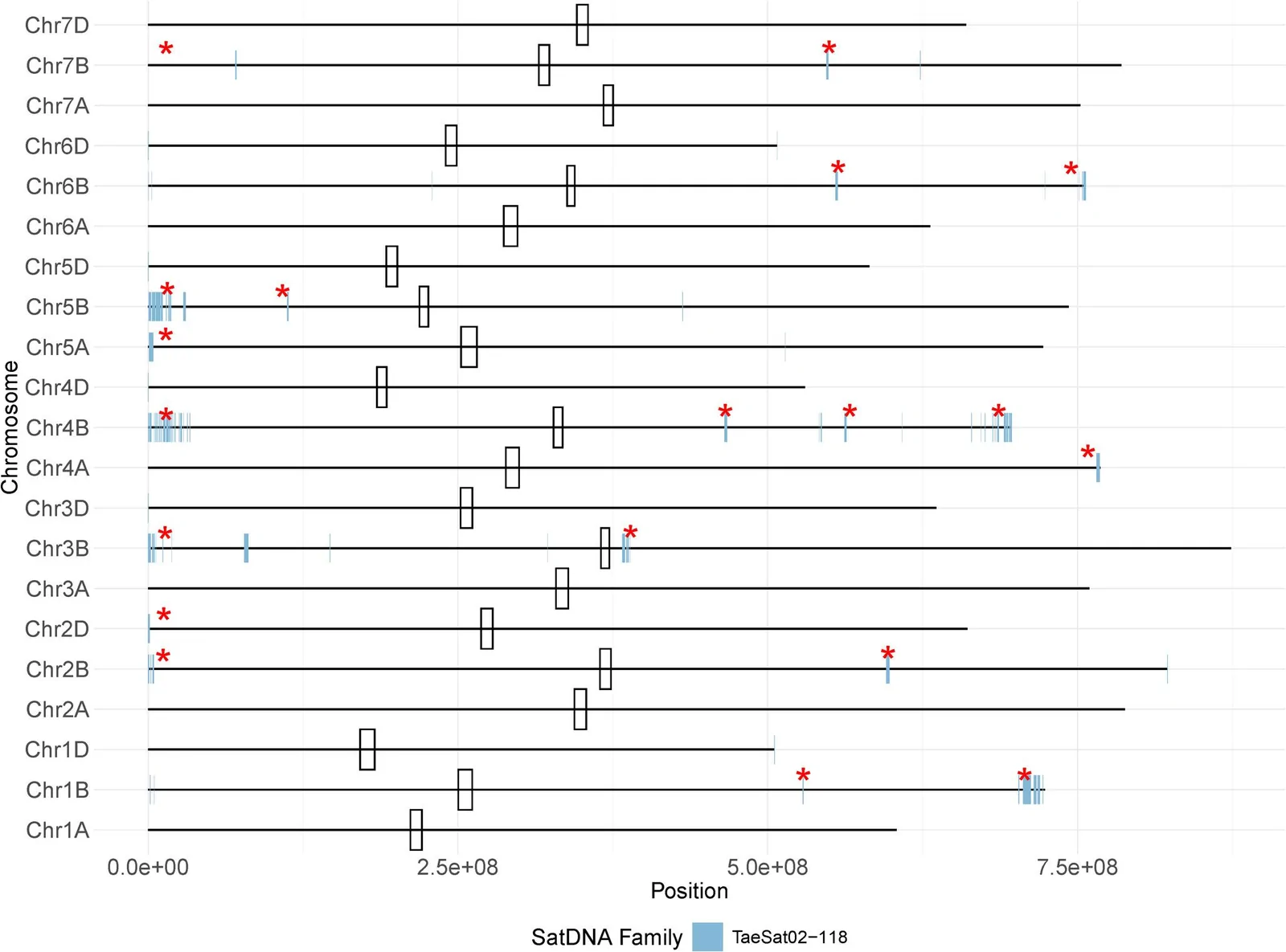
*T. aestivum* cv. Chinese Spring chromosomes representation showing the distribution of TaeSat02-118 satDNA family as obtained through the CHRISMAPP approach. The centromeres of each chromosome are indicated as boxes. Asterisks indicate loci detected in Gálvez-Galván et al. (2024a) using FISH. The graphic only includes loci that comprise at least one full repeat unit

**Fig. 2:**
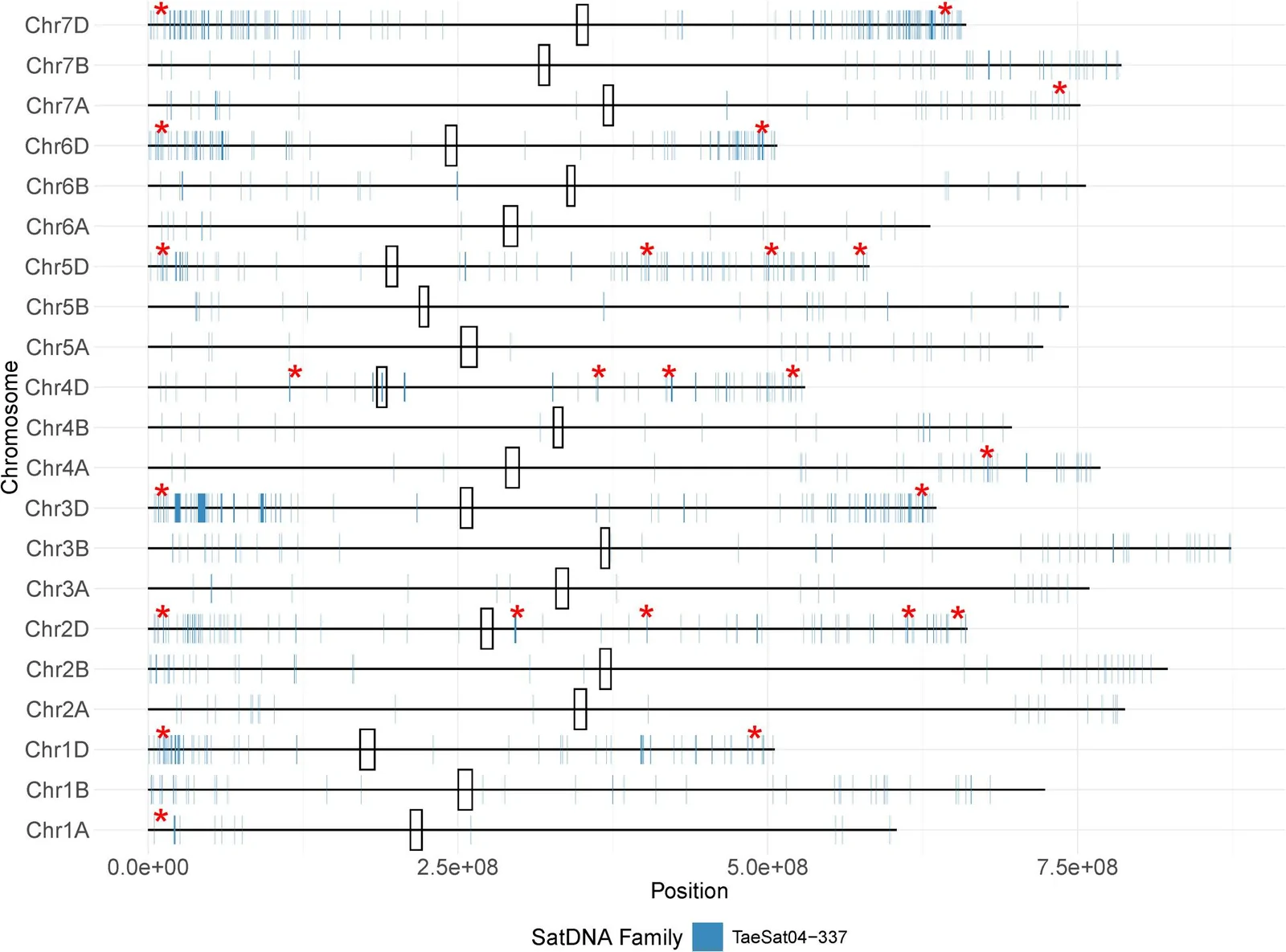
*T. aestivum* cv. Chinese Spring chromosomes representation showing the distribution of TaeSat04 − 337 satDNA family as obtained through the CHRISMAPP approach. The centromeres of each chromosome are indicated as boxes. Asterisks indicate loci detected in Gálvez-Galván et al. (2024a) using FISH. The graphic only includes loci that comprise at least one full repeat unit

**Fig. 3:**
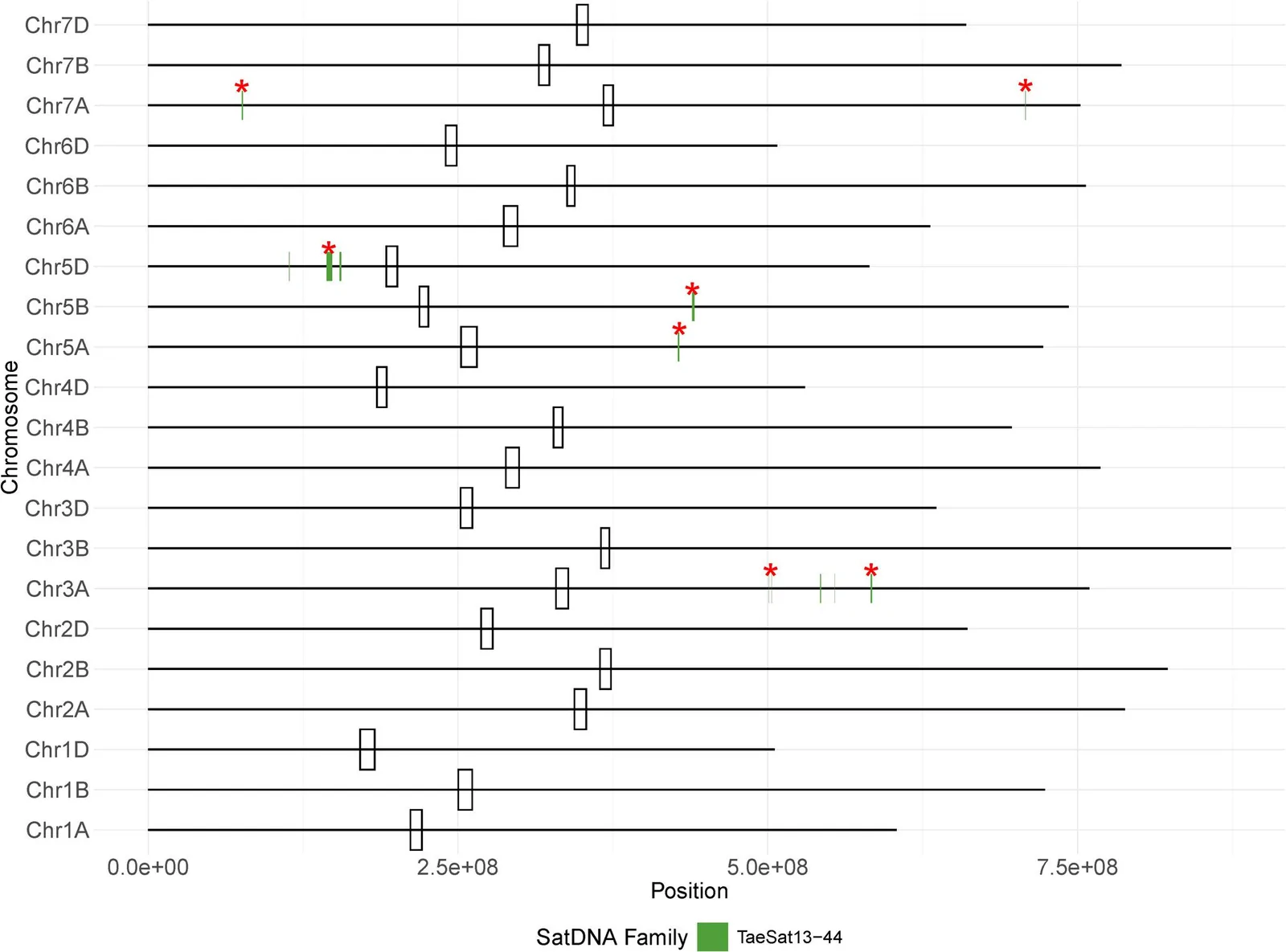
*T. aestivum* cv. Chinese Spring chromosomes representation showing the distribution of TaeSat13 − 44 satDNA family as obtained through the CHRISMAPP approach. The centromeres of each chromosome are indicated as boxes. Asterisks indicate loci detected in Gálvez-Galván et al. (2024a) using FISH. The graphic only includes loci that comprise at least one full repeat unit

Table 1 summarizes the different organizational patterns of the 36 satellites in the wheat genome assembly together with their relationships with TEs (see also Table S1 for details). These patterns were inferred from RepeatMasker analysis of the T2T assembly using the full satellitome including the 36 satDNA families as database (Table S2) and from the graphical visualization of these results (Figs. 1, 2, 3 and Figure S1).

**Table 1:** Genomic patterns of *T. aestivum* cv. Chinese spring satDNA families

Satellite name	Repeat unit length (nt)	Location according FISH	Nº full-length copies	Genomic pattern*	TE homology (% coverage)
TaeSat01-584	584	Dispersed	15,626	C’	CACTA (100)
TaeSat02-118	118	Multiple locations	517,906	A^$^	
TaeSat03-2619	2619	Terminal	126	B^#^	Gypsy (17)
TaeSat04-337	337	Multiple locations	140,478	A^$^	CACTA (100)
TaeSat05-500	500	Dispersed	108,483	B^$^	CACTA (100)
TaeSat06-403	403	Dispersed	46,206	C’	Gypsy (99)
TaeSat07-343	343	Multiple locations	101,770	A^$^	CACTA (100)
TaeSat08-663	663	Terminal	25,369	B^$^	CACTA (100)
TaeSat09-335	335	Multiple locations	31,249	A^$^	CACTA (100)
TaeSat10-206	206	Multiple locations	12	C	CACTA (100)
TaeSat11-506	506	Dispersed	20,125	B^$^	CACTA (100)
TaeSat12-369	369	Terminal	20,378	A^#^	
TaeSat13-44	44	Multiple locations	183,387	A^#^	
TaeSat14-1463	1463	(Peri)centromeric	844	B^#^	Mutator (100)
TaeSat15-620	620	Multiple locations	2398	C	Gypsy (87)
TaeSat16-567	567	(Peri)centromeric	4637	B^$^	
TaeSat17-323	323	Terminal	7506	B^$^	Mutator (100)
TaeSat18-733	733	Multiple locations	2879	B^#^	
TaeSat19-653	653	Multiple locations	1544	B^$^	CACTA (97)
TaeSat20-322	322	Terminal	2663	B^$^	CACTA (100)
TaeSat21-1590	1590	Dispersed	439	C	Gypsy (100)
TaeSat22-320	320	Terminal	1769	B^$^	
TaeSat23-319	319	Terminal	2014	B^$^	Mutator (100)
TaeSat24-338	338	NA	1772	C	CACTA (100)
TaeSat25-318	318	Multiple locations	1271	B^$^	Mutator (100)
TaeSat26-210	210	Terminal	4572	B^#^	
TaeSat27-72	72	Terminal	8524	A^#^	
TaeSat28-543	543	(Peri)centromeric	1252	B^$^	
TaeSat29-319	319	Multiple locations	920	C	Mutator (100)
TaeSat30-1389	1389	Multiple locations	155	B^#^	
TaeSat31-889	889	(Peri)centromeric	569	B^#^	
TaeSat32-528	528	Terminal	491	B^#^	
TaeSat33-54	54	Multiple locations	2908	B^#^	
TaeSat34-175	175	Multiple locations	397	B^#^	
TaeSat35-1390	1390	Terminal	85	B^#^	Mutator (21)
TaeSat36-1242	1242	Terminal	12	B^#^	

The table includes Repeat unit length, location according to FISH (taken from Gálvez-Galván et al. 2024a), total number of full-length copies per satDNA estimated in this study, and the homology to transposable elements (TEs), indicating what percentage of the total length of the satDNA sequence is homologous to the corresponding TE (% coverage)

^*^See text for details

Assuming that a long array consists of 1000 or more RUs (see Materials and Methods), overall, two major types of organization can be distinguished. That is satellites organized both into long and short arrays (pattern A) and others that are organized into a variable number of short arrays (pattern B). Thus, in pattern A, seven satDNAs are organized into long and short arrays. Four of them (A^$^) are organized into numerous long and short arrays in addition to very short arrays, composed of less than ten repeat units, as well as isolated copies distributed across most or all chromosomes. These four satellites are among the most abundant families (those 9 above 0.1% of the genome) and three of them are derived from CACTA elements. The three remaining satellites in this group (A^#^) are organized into a few arrays rather than numerous ones and are not related to TEs. In contrast, 22 satDNAs show the B pattern: satDNAs are organized into short arrays. Eleven of them (B^$^) are distributed across numerous short arrays, including many very short arrays (< 10 repeat units each) and single repeats scattered throughout the genome. The other eleven (B^#^) are organized just into one or few short arrays, with occasionally few additional very short arrays (< 10 repeat units each). Three B-pattern satellites are among the most abundant in the genome, and eleven showed homologies to CACTA or MuDR transposons, or to Gypsy retrostranspons. In addition, seven repetitive sequences (C pattern) derived from CACTA, MuDR, or Gypsy elements occur exclusively as very short arrays of fewer than 10 repeat units each, scattered throughout the genome. Within this class, two repetitive sequences are highly abundant compared to the others: a CACTA transposon (TaeSat01-584) and a Gypsy retrotransposon (TaeSat06-403). These two sequences occasionally appear duplicated, forming a few dimers, trimers, or tetramers. They could represent a distinct subclass of the C pattern (C’).

**Fig. 4:**
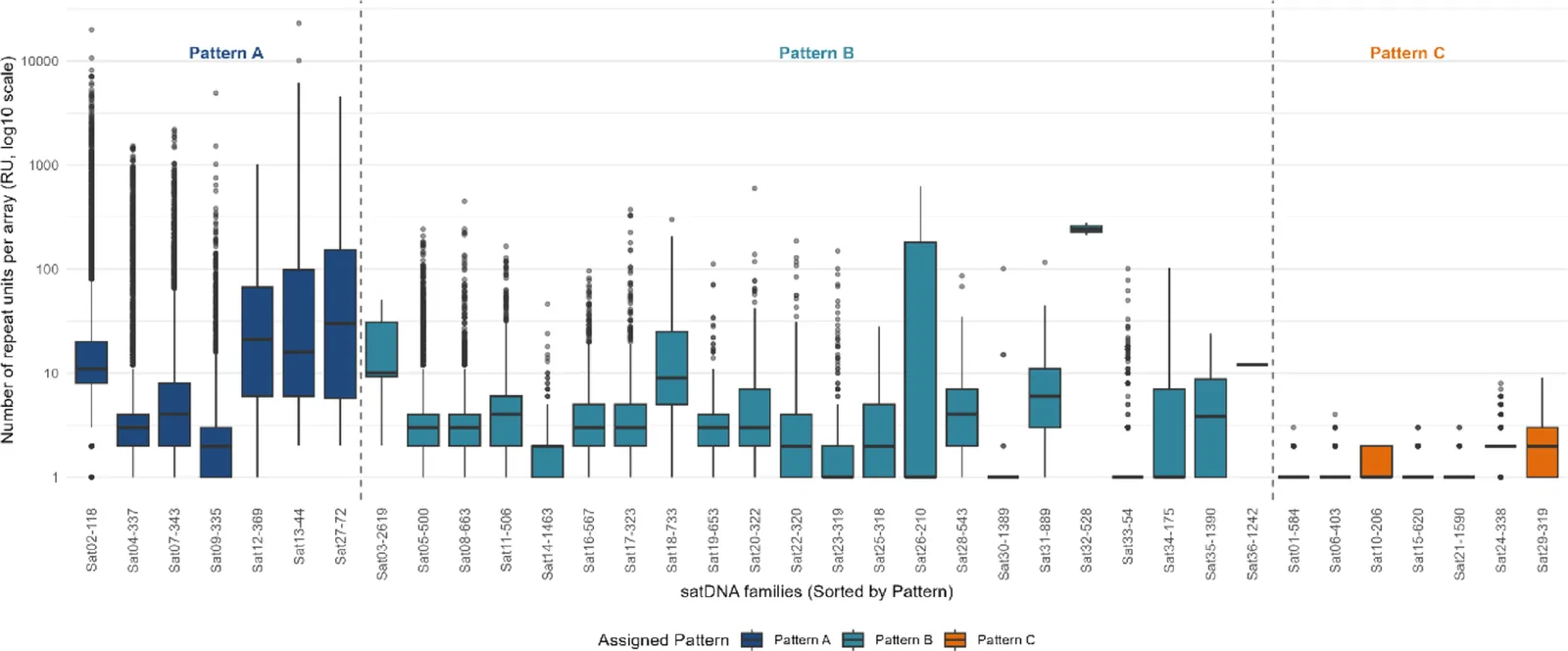
Distribution of the number of repeat units (RUs) per array for each individual satDNA family sorted by the defined structural patterns. Families are grouped and color-coded according to their assigned structural pattern

Figure 4 illustrates graphically the distribution of the number of repeats per array for each individual satellite sorted by the defined structural patterns. The detailed breakdown of the 36 satellite DNA families reveals a clear stratification in the number of repeat units (RU) per array when organized according to their assigned structural patterns. Families grouped under Pattern A occupy the leftmost portion of the plot and exhibit the widest distribution verticality, encompassing arrays with up to 10^4^ repeat units alongside isolated low-RU outliers. In the center, families assigned to Pattern B show an intermediate and stabilized distribution, with interquartile ranges consistently maintained between approximately 10^1^ and 10^2^ RU. Conversely, the rightmost block representing Pattern C is characterized by a marked compression of all boxplots, which are strictly restricted below the 10^1^ threshold. This global arrangement confirms that individual family behavior remains highly uniform within each partitioned structural group.

**Fig. 5:**
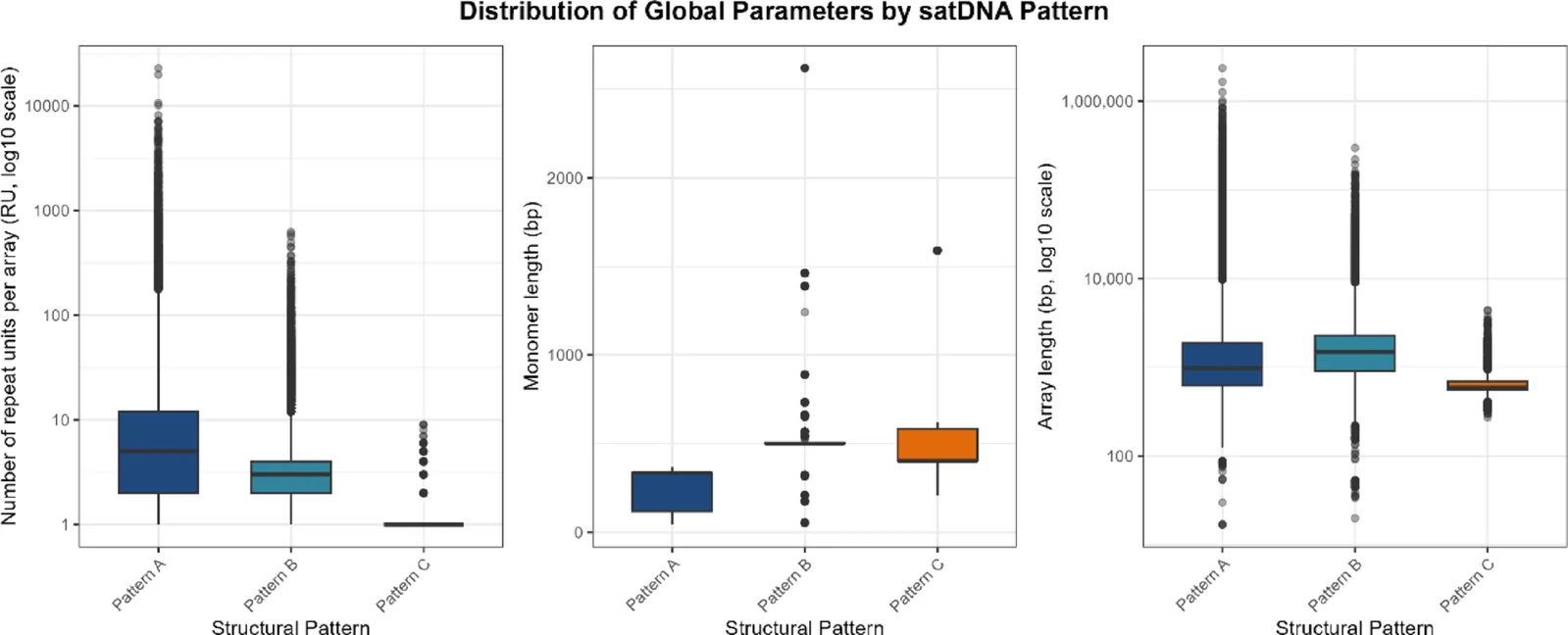
Distribution of global parameters (number of repeat units per array, monomer length and array length) by satDNA pattern. Families are grouped and color-coded according to their assigned structural pattern

Figure 5 illustrates the distribution of global parameters (number of repeat units per array, monomer length, and array length) by satDNA pattern. Regarding the number of repeat units per array (left-hand panel), distinct structural behaviors were observed among the three groups. As in Fig. 4, Pattern A, exhibited the highest variability and the most extreme values; despite its low median value and interquartile ranges between 2 and 12, it displayed a continuous distribution of outliers exceeding 10,000 units per array, demonstrating a dual behavior that signals a highly dynamic evolutionary regime driven by localized expansions. Conversely, Pattern B showed a more constrained dispersion, and a slightly lower median, and narrower interquartile ranges (between 2 and 4), though it still retained a tail of outliers reaching 624 repetitions (three outliers above 500 RUs). Finally, Pattern C was characterized by high homogeneity, with its distribution collapsing strictly at a value of 1, indicating that the vast majority of these satellites occur as single units, with only a few outliers ranging from 2 to 9 repeats. Regarding monomer length (central panel), the analyzed patterns revealed distinct size profiles. Pattern A was characterized by having the shortest repetitive units, with a median positioned at 335 bp and its entire distribution strictly confined below 400 bp. In contrast, Pattern B, which displays the widest variation in monomer size, showed a median of 535.5 bp and exhibited a wide dispersion of outliers ranging from very short lengths to an isolated maximum of 2619 bp. Finally, Pattern C presented a median of 403 bp and a wide dispersion. This central panel provides insight into the criteria used to establish the patterns. Although structural patterns could alternatively be defined by total array length (right-hand panel), using the number of repeat units (RUs) per array offers a far more objective classification. Array length is a compound metric driven by both RU count and monomer length. However, as monomer length is highly variable, relying solely on total array length can be misleading: an array can achieve substantial length with very few but exceptionally long monomers. Conversely, an array of many copies may remain relatively short if its monomer size is minimal. Therefore, defining patterns based strictly on the number of RUs effectively isolates the true dynamics of tandem duplication from the confounding variable of monomer size.

Historically, satDNAs were thought to be mainly organized into long clusters located in heterochromatin regions (reviewed in Garrido-Ramos 2017; Šatović-Vukšić and Plohl 2023; Garrido-Ramos et al. 2025). This view became obsolete when it was discovered that many satDNA also formed short arrays and that could appear even as isolated copies scattered throughout the euchromatin (reviewed in Garrido-Ramos 2017; Šatović-Vukšić and Plohl 2023; Garrido-Ramos et al. 2025), which could affect the expression of nearby genes (Garrido-Ramos et al. 2025). This is especially relevant today, given that analyses of satellitomes from a wide variety of animal and plant species have revealed the existence of satDNAs organized exclusively in short arrays, and that both short and long arrays may be uncoupled from heterochromatin (reviewed in Garrido-Ramos et al. 2025). Indeed, genomes contain multiple satDNAs composed of either long or short repeat units, arranged in long or short arrays, and located in both heterochromatin and euchromatin. The different organizational levels observed within a species' satellitome support the model proposed by Ruiz-Ruano et al (2016), in which a satDNA originates by the de novo duplication of a genomic sequence that produces a short array at a single genomic location, followed by dissemination throughout the genome and local amplification, becoming into a clustered satDNA. While amplification broadly denotes the proliferation of repeat units both at the original locus and across the genome —a localized expansion largely mediated by unequal sister chromatid exchange, though mechanisms like replication slippage may also be involved— the spreading and colonization of novel genomic loci is primarily driven by transposition events and the chromosomal reintegration of sequences synthesized via the rolling-circle replication of extrachromosomal circular intermediates, which in turn inherently contribute to the overall amplification of satDNA families (Garrido-Ramos 2017, 2021). The study of the bread wheat satellitome suggests a prominent role for TEs in the origin of many satDNAs and allows us to link the observed patterns to specific evolutionary stages. Thus, pattern C would represent a starting point at which part of a TE is duplicated to produce tandems of two or a few repeats. Patterns B^$^ and A^$^ represent minor or major clustering across multiple dispersed loci throughout the genome, producing arrays of different lengths. Of course, other types of random sequences, besides TEs, could also follow these specific evolutionary stages of the model, since 40% of the satellites have not shown homology with TEs. In fact, most satellites with pattern A^#^ or B^#^, that have been amplified in only one or a few loci, show no homology with TEs (11 out of 14) (Table 1). Furthermore, most of these 14 satDNAs are not disseminated throughout the genome but have been amplified, probably recently, in only one or a few chromosomes (Table 1 and Figure S1). Their future dissemination throughout other chromosomes remains uncertain. If this is the case, amplification would precede spread, contrary to the sequence proposed by Ruiz-Ruano et al. (2016). Conversely, most of the widely disseminated satellites belonging to types A^$^ or B^$^ are related to TEs. These satellites have clustered loci of varying lengths on many chromosomes and are therefore likely older. Specifically, eleven of the fifteen satellites in these categories are TE-related. However, for the remaining four, we cannot rule out that their sequences have diverged too extensively, or are too short, for RepeatMasker to detect similarity to their ancestral TE reliably.

As discussed by Gálvez-Galván et al. (2024a), the seven repetitive sequences with C pattern do not strictly fit the classic concept of satellite DNA. Although they were identified as such using RepeatExplorer2/TAREAN—and some, like TaeSat01-584 or TaeSat06-403, were among the most abundant in the genome—by definition, we are dealing here with highly repetitive sequences that cannot be rigorously classified as satellite DNA. Nevertheless, because they are distributed individually throughout the genome and also form very short arrays at specific coordinates, they can be considered the germ of future satellites. Crucially, this hypothesis is supported by the case of TaeSat05-500 and TaeSat11-506. While these two satellites still show dispersed FISH patterns (see below) and are indeed scattered across the genome (Table S2, Figs. 4 and S1) due to their TE-derived origin, our findings in this paper confirm that they form dozens of arrays with over 100 RUs and thousands of arrays with more than 10 RUs (Table S2, Figs. 4 and S1). Therefore, they represent a further evolutionary step, demonstrating how short, TE-derived tandem repeats can progressively expand into large arrays and ultimately constitute true satDNA families.

Table 1 also presents the numbers of full-length copies for each satDNA family based on the estimates from the present study. Comparing these results with the data obtained by Gálvez-Galván et al. (2024a) reveals substantial discrepancies between some of the estimates. This variation is particularly striking for satellites such as TaeSat01-584, TaeSat03-2619, TaeSat07-343, TaeSat10-206, TaeSat14-1463, TaeSat15-620, TaeSat21-1590, TaeSat23-319, TaeSat24-338, TaeSat25-318, and TaeSat29-319, all of which are sequences related to TEs. In Gálvez-Galván et al. (2024a), the copy number was estimated by calculating the genomic proportion of each satDNA, which involved mapping 20 million randomly selected sequencing reads to the satDNA database and normalizing the aligned nucleotides against the total sequenced pool. Consequently, those estimates were likely overestimated due to the detection of numerous mobile element fragments dispersed throughout the genome. For instance, regarding TaeSat01-584 and TaeSat03-2619, in addition to the full-length copies (Sheets 3 and 5 of Table S2), RepeatMasker identified over 150,000 matches shorter than the monomer and more than 300,000 short fragments (26–1,800 bp) scattered across the genome, respectively (Sheet 2 of Table S2).

### satDNA arrays in the T2T assembly and FISH results

Five repetitive sequences of the bread wheat satellitome showed a scattered FISH pattern throughout the genome (Gálvez-Galván et al. 2024a). All of them were TE-related sequences and, depending on the locus, they were duplicated (TaeSat01-584 and TaeSat21-1590), formed short arrays of no more than 4 repeat units (TaeSat06-403), or formed arrays of different lengths, including tens or hundreds of repeats (TaeSat05-500 and TaeSat11-506) characteristic of classical satDNA arrays (Table S3; Figs. 4, S1 and S2). Based on their length and location, some TaeSat05-500 and TaeSat11-506 loci, particularly those in pericentromeric or interstitial regions, should have been visible by FISH. However, they were not, although stronger signals of TaeSat05-500 were observed in (peri)pericentromeric regions from D subgenome (Gálvez-Galván et al. 2024a). In addition, TaeSat24-338 derived from a CACTA transposon, was detected by RepeatMasker in more than 9,000 matches in the assembled genome, mostly as isolated fragments smaller than its monomeric unit (338 bp) and about 561 matches of very short arrays of 2–8 repeats. A suitable probe for FISH of this repetitive sequence was not possible to amplify by PCR. If available, it would have clearly given a scattered pattern (Gálvez-Galván et al. 2024a). TaeSat14-1463, a MuDR-derived transposon sequence, also belongs to this category. There are 37,363 loci of this sequence spread over all chromosomes, most as isolated units. Only 155 consisting of short arrays (2–53 repeat units), located only at the end of chromosome 2D. None of them occurred at the centromere, despite the discrete bands detected by FISH in each centromere of the genome, in addition to a scattered pattern (Gálvez-Galván et al. 2024a; see also below).

FISH results for another 26 satDNAs (Gálvez-Galván et al. 2024a) were broadly consistent with their distribution in the T2T assembly, although some discrepancies exist (Figs. 1, 2, 3 and Figure S1). Indeed, it is difficult to establish the minimum length required for FISH detection. Detection thresholds vary among studies (Ruiz-Ruano et al. 2016; Dos Santos et al. 2021; Camacho et al. 2022) and likely depend on factors such as chromatin condensation, three-dimensional organization, probe specificity, hybridization efficiency and array fragmentation. Many arrays undetected by FISH in wheat were several kilobases long, indicating that array continuity and homogeneity may be as important as total length. Large genomic regions, both FISH-positive and FISH-negative, often appear as fragmented loci consisting of numerous short arrays, or mixtures of long and short arrays separated by intervening sequences of variable length. Sometimes these sequences are shorter and follow a regular pattern, revealing RepeatMasker's inability to detect length variants of a satDNA (see below), but other times they reflect a pattern that may consist of either inconsistencies in the assembly in highly repetitive regions or a true scattered pattern of nearby arrays within a specific region.

Among these 26 satDNAs, with generally consistent results, the most abundant (TaeSat02-118, TaeSat04-337, TaeSat07-343, TaeSat08-663 and TaeSat09-335) are distributed across numerous long and/or short arrays that are close together and located at terminal and/or interstitial sites on various chromosomes. These arrays are visualized by FISH as a single large block, due to metaphase chromosomes compaction. Conversely, some arrays of these satellites detected in the T2T assembly were not revealed by FISH. Less frequent satDNAs with a few FISH loci in some chromosomes (TaeSat12-369, TaeSat16-567, TaeSat17-323, TaeSat19-653, TaeSat20-322, TaeSat22-320, TaeSat23-319, TaeSat28-543 or TaeSat35-1390), also show additional arrays in the assembly that were not detected at the cytogenetic level. For example, TaeSat35-1390 shows one FISH locus on chromosome 7D (Kapustová et al. 2019), but the T2T assembly reveals an additional one on 7A. TaeSat16-567, appears now (peri)centromerically in more chromosomes than those indicated in Gálvez-Galván et al. (2024a; see below). In some of these cases, there is also some discrepancy between the interpretation after FISH and the location in the genome assembly. For example, the TaeSat12-369 FISH loci previously consider distal/subtelomeric are interstitial in the T2T assembly; the TaeSat28-543, which showed strong centromeric FISH signals in seven chromosomes and weaker FISH signals in the rest of the chromosomes (Gálvez-Galván et al. 2024a), appear (peri)centromerically in eleven chromosomes in the T2T assembly. In several cases, arrays detected in the assembly seem too short to produce FISH signals. However, as occurs with TaeSat23-319, short arrays clustered very close together are separated by sequence fragments of the same length (~ 192–535 bp), especially in chromosomes 2B, 4B, 5B, and 6B (all FISH positives). Looking at the sequence of these segments, we confirmed that they consist of length variants of the TaeSat23-319 satellite irregularly interspersed between tandems of canonical sequence, so the arrays are longer than predicted by RepeatMasker. Similar patterns occur also in several other satDNAs and will be discussed below. There is a high degree of agreement between the FISH and T2T results for 8 of these 26 satDNAs (TaeSat03-2619, TaeSat13-44, TaeSat18-773, TaeSat26-210. TaeSat27-72, TaeSat30-1389, TaeSat32-528 and TaeSat36-1242), which typically are organized in one or a few short arrays, accompanied by isolated complete or incomplete monomers. Finally, four satDNAs (TaeSat25-318, TaeSat29-319, TaeSat33-54 and TaeSat34-175) showed several discrepancies between the FISH and T2T results. For example, TaeSat33-54 arrays are found in chromosomes 3A, 3D, 4A and 4D but not in chromosome 7D, contrasting with FISH signals in chromosomes 4A, 4D and 7D (Gálvez-Galván et al. 2024a), maybe due to the difficulties of distinguishing chromosomes 3D and 7D based on the pAs1 and GAA patterns (Pedersen & Langridge 1997), although T2T assembly should be also revised.

### Relevant incongruencies between satDNA arrays in the T2T assembly and FISH results

As mentioned above, TaeSat14-1463 showed a dispersed pattern across all chromosomes, as well as conspicuous bands in the centromeres of all bread wheat chromosomes (Gálvez-Galván et al. 2024a). The same result was obtained for durum wheat when the homologous sequence TtuSat13-1463 was used as a probe (Gálvez-Galván et al. 2024b). TaeSat14-1463 sequence is entirely homologous to a MuDR-45_TAe DNA transposon fragment, which explained its dispersed pattern throughout the genome. The centromeric FISH signals could be due to cross-hybridization with transposable elements in centromeres since this sequence is absent from the assembled centromeres. However, although centromeres are plenty of TEs, most of them are Gypsy elements and, in a smaller proportion, CACTA elements (Liu et al. 2025; see also below).

Three additional repetitive sequences (TaeSat10-206, TaeSat15-620 and TaeSat31-889) also showed a significant discrepancy between the T2T assembly and earlier FISH observations (Gálvez-Galván et al. 2024a). Only three dimers and more than 1150 monomers of TaeSat10-206 are present in the T2T assembly. However, FISH revealed conspicuous bands in 11 chromosome pairs, which would be compatible with 11 long arrays of TaeSat10-206 sequences, clearly incompatible with the assembly data. TaeSat10-206 belongs to superfamily 1 (SF-1) like TaeSat04-337 and TaeSat07-343. TaeSat04-337 and TaeSat07-343 share 82.2% similarity and exhibit 98% and 82.5% similarity, respectively, to the alignable regions of TaeSat10-206, given that the latter is a repetitive sequence that lacks an amplified region present in the other two (Gálvez-Galván et al. 2024a). These high levels of homology strongly suggest that the FISH pattern can only be explained by cross-hybridization with TaeSat04-337 sequences.

TaeSat15-620 repeat units are composed of four 155 bp sub-repeats (Gálvez-Galván et al. 2024a). However, in the genome assembly, only 103 scattered dimers and one trimer of the 620-bp repeat unit are found, along with about 15,000 isolated loci composed of 1–4 copies of the 155-bp repeat dispersed around the genome. FISH revealed a dispersed pattern of this satDNA (which would be compatible with the T2T assembly and with the fact that this sequence is related to Gypsy elements; Tables 1 and S1), together with several conspicuous bands in 7 chromosome pairs, which does not seem explainable according to the T2T assembly analysis.

TaeSat31-889 was visualized by FISH in all the chromosomes of the B subgenome from pericentromeric to interstitial regions (Gálvez-Galván et al. 2024a). However, the homologous durum wheat TtuSat24-889, was only detected in the pericentromere of chromosome 1B (Gálvez-Galván et al. 2024b), which coincides with our observation in the T2T genome assembly of bread wheat (Figure S1). In the assembly, most TaeSat31-889 arrays on chromosome 1B were separated by short 20–70 bp DNA fragments. A detailed analysis showed that they correspond to TaeSat31-889 length variants such as those seen in other satellites (see below) and that the whole region should be a single longer array (Table S2 and Figure S1). In this case, these fragments interspersed in the sequence of this satellite correspond to microsatellites (TC)_n_ and (TC)_n_(TA)_n_, which might be interpreted as resulting from expansions of some of the microsatellites that forms part of the monomeric sequence of TaeSat31-889 [(TC)_7_, (TC)_2_, (TA)_2_, and (TA)_3_]. It is therefore possible, although unlikely, that hybridization results observed in Gálvez-Galván et al. (2024a) could include cross-hybridization with regions rich in this type of microsatellites.

### Sequence length variations

RepeatMasker analysis of the T2T genome assembly revealed remarkable patterns for 11 satDNAs (TaeSat02-118, TaeSat09-335, TaeSat12-369, TaeSat13-44, TaeSat22-320, TaeSat23-319, TaeSat26-210, TaeSat30-1389, TaeSat31-889, TaeSat33-54 and TaeSat34-175). In all cases, Table S2 shows genomic regions where multiple long and/or short arrays are organized in close proximity, separated by a short, apparently unrelated, intervening sequence. However, a detailed analysis demonstrated that these intervening sequences are, in fact, a short fragment of the repetitive unit, revealing sequence length variants giving sites where canonical repetitive units and length variants coexist. This type of mosaic structure is consistent with established links between tandem repeat polymorphisms -including microsatellites and satDNAs- and recombination hotspots (Skowronski et al. 1984; Brandström et al. 2008; Wahls 2011; Saayman et al. 2023).

For example, TaeSat02-118 satDNA was organized in several distinct regions into multiple arrays, each separated by a short DNA segment of a specific length. Although this intervening segment length is consistent within a given region, it varies among different chromosomal regions (64 bp, 72 bp, 111 bp, 119 bp, 120 bp, etc.). These fragments appear irregularly interspersed between canonical arrays, of varying lengths (Table S2). A detailed analysis of these regions demonstrates that these arrays are longer than detected because the intervening sequence is part of the array (the intervening sequences are repeat unit length variants). In these regions, sequence length variants are interspersed with other sequence variants and canonical sequences (Figure S3). Similar patterns are found for TaeSat12-369, with inter-array fragments of 13 bp, 101 bp, etc., and in TaeSat13-44 (34 bp, 36 bp, 45 bp, 52 bp, 120 etc.), where RepeatMasker mainly detected very short arrays of this satDNA in the sites previously detected by FISH (Gálvez-Galván et al., 2024a). A detailed analysis of the regions demonstrates that these arrays are longer than detected since the intervening sequences are repeat unit length variants of TaeSat13-44 (Figure S4). Sequence length variants of TaeSat22-320 (~ 334–366 bp) and TaeSat23-319 (~ 192–535 bp) were also observed.

Several satellites (TaeSat09-335, TaeSat26-210, TaeSat30-1389, TaeSat31-889, TaeSat33-54 and TaeSat34-175) deviate from these patterns and present distinctive features. Thus, in several chromosomes (see, for example, chromosome 1A), many dimers of TaeSat09-335 are separated by a completely different sequence but of similar length. A detailed analysis shows that in these regions, the 335 bp sequence is part of another new satellite of ~ 1000 bp composed of two TaeSat09-335 monomers (~ 10% divergence between them) plus a new related sequence (~ 53% divergence) of similar length (Figure S5). Numerous combinations of TaeSat09-335 dimers, trimers, and larger multimers are interspersed with this new trimeric satellite (Table S1).

TaeSat26-210 also shows a complex organization (Figure S6). There are two variants of this satellite, a short 210 bp version and a longer, more abundant 362 bp variant. Both are randomly interspersed in chromosome 1B locus.

TaeSat30-1389 had two interstitial FISH sites (Gálvez-Galván et al. 2024a), one in chromosome 5A and one in 5D, both confirmed by this analysis. In chromosome 5D, we detected an array of 100 repeat units (139,869 bp) alongside a nearby region containing 7 isolated repeats separated by 1983–1986 bp sequences (Table S1). Detailed analysis of this region revealed longer variants of TaeSat30 satDNA of ~ 1681–1688 bp, composed of one 1389 unit (1383–1391 bp depending on indels) plus a ~ 296–298 bp fragment, forming an array of 13 repeat units (Figure S7). An array of 24 repeat units of this longer variant corresponds to the 5A FISH locus (in this case, the RepeatMasker output appears as several consecutive ~ 1389 units separated by the characteristic ~ 298 bp segment).

As mentioned above, length variations in TaeSat31-889 would be due to microsatellite expansions within the repeat unit, consistent with replication slippage (Lai and Sun 2003; Leclercq et al. 2010).

In the assembled genome, RepeatMasker detects only a few dimers and trimers of TaeSat33-54, never higher multimers. However, the regular spacing of isolated units suggests that they may form part of longer sequences of different lengths (see Table S1). A detailed analysis of these regions reveals homologous repeats of ~ 425 bp, ~ 846 bp and ~ 956 bp containing several TaeSat33-54 copies. Tandem Repeat Finder (TRF) (Benson 1999), demonstrated that these longer repeats are composed of ~ 108 bp subrepeats, each composed of two TaeSat33-54 units, the second of which is partially degenerated such that identity between the two halves have decreased (Figure S8). In other words, this satellite evolves from short 54 bp units to longer 108 bp units through duplication-divergence cycles as observed in other long satellites that derive from shorter ones (Navajas-Pérez et al. 2005; Sales‑Oliveira 2024; Garrido-Ramos et al. 2025). Indeed, 28 of the 34 satDNAs analyzed in Gálvez-Galván et al (2024a) are composed of sub-repeats, indicating that most of the current wheat satellites derive from shorter ones. Interestingly, RepeatExplorer detected for TaeSat33-54 satDNA only the basic 54-bp sequence rather than other longer derived satellites.

TaeSat34-175 forms very short arrays (< 10 repeats) immersed within highly repetitive genomic regions. Dot plots of these regions (Figure S9) reveal periodic repeated segments in which repeat lines are interrupted or shifted and vary in length and position, suggesting the coexistence of both identical and variant repeats within these segments resulting from small changes, insertions, or deletions between repetitive units, typical of dynamic genomic regions, which could indicate frequent mutations and/or recombinations in the area. The GTTCAC microsatellite is highly represented throughout the region, including five copies within the TaeSat34-175 repeat unit. A detailed analysis of these regions reveals that TaeSat34-175 is the irregularly repeating sequence. There are many sequence and sequence length variants of TaeSat34-175, interspersed with complete units or forming incomplete tandems of two or three repeat units (Table S2 and Figure S9).

### Centromeric and pericentromeric satDNAs

**Fig. 6:**
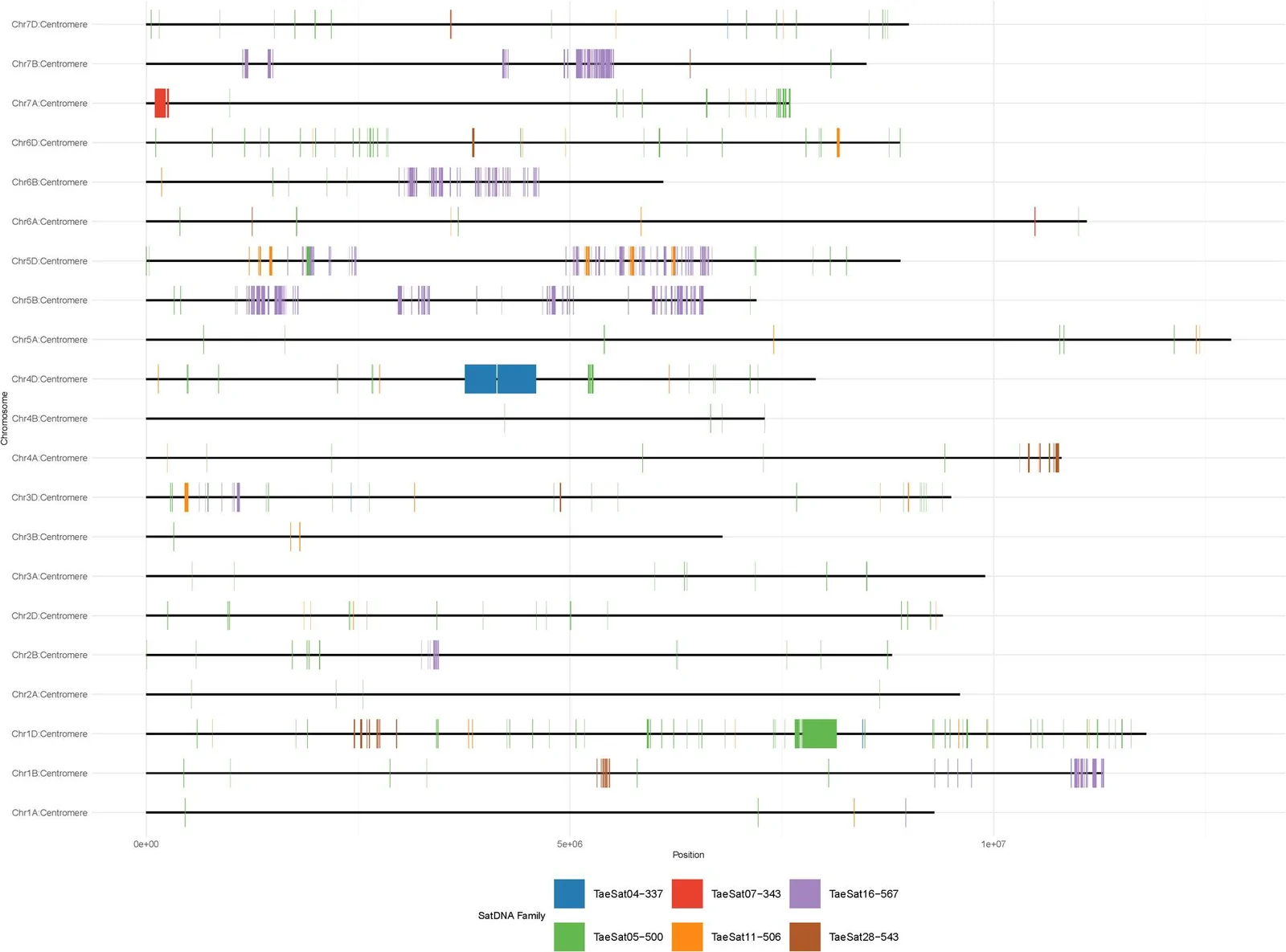
*T. aestivum* cv. Chinese Spring centromeres representation showing the distribution of the six satDNA families forming arrays of different sizes in different chromosomes as obtained through the CHRISMAPP approach

In total, TEs represent, on average, ~ 94% of the wheat centromeres, with differences among subgenomes (Liu et al. 2025): 87–98.6% depending on the subgenome. They are mainly Gypsy retrotransposons (68–87%, depending on the subgenome) while Copia retrotransposons (4.1–7.9%) and CACTA elements (3–11%) constitute smaller fractions. The remainder are tandem repeat sequences according to Liu et al. (2025). We have found an important number (18) of bread wheat satDNAs in the centromeres of all chromosomes (Table S4, Fig. 6, Figure S2). In total, they represent 3.38% (6,504,547 bp) of the centromeres, considerably higher than the percentage found by Liu et al. (2025): 1.1% (0.1–2.6%, depending on the subgenome). However, only divergent isolated and mostly incomplete repeats, with occasional dimer or tetramer, were found for 12 of these 18 satDNAs (Table S4). The remaining six satellites are organized into typical satDNA arrays, but isolated copies or very short arrays of each of them are also scattered throughout the centromeres (Table S4 and Figs. 6 and S2). Thirteen of the 18 centromeric satellites showed homology to TEs (10 were related to DNA transposons of the CACTA type and 3 to Gypsy retrotransposons. Many of these scattered copies detected by RepeatMasker may correspond to homologous parts of TEs, explaining part of the differences between our results (Tables S1 and S4) and those of Liu et al. (2025).

As mentioned above, six satDNAs form arrays of different sizes in different chromosomes (Figs. 6 and S2): TaeSat04-337, TaeSat05-500, TaeSat07-343, TaeSat11-506, TaeSat16-567 and TaeSat28-543. Only the last two were detected as centromeric satDNAs by FISH together with TaeSat14-1463 and TaeSat31-889 (Gálvez-Galván et al. 2024a). These last two represent inconsistencies already discussed.

Of the other four, TaeSat04-337 is organized in seven contiguous arrays of 22–1101 repeat units in the centromere of chromosome 4D. These arrays should have been visible using FISH. Although this satellite was correctly identified by Gálvez-Galván et al. (2024a) as a multi-locus satellite with a large number of terminal and interstitial loci, the centromeric locus on 4D was misinterpreted as interstitial, due to the lack of precise centromere coordinates at that time (now available from Liu et al. 2025).

TaeSat07-343 forms several arrays of 9–146 repeat units closely located in the centromere of chromosome 7A and an array of 21 repeats in the centromere of chromosome 6A. FISH identified multiple subtelomeric and interstitial loci distributed throughout most of the chromosomes but not these two centromeric one (Gálvez-Galván et al. 2024a), likely because they were masked by stronger non-centromeric signals.

TaeSat05-500 and TaeSat11-506 were previously identified by FISH as dispersed sequences. However, the T2T assembly also shows that both satellites were organized in short arrays throughout the genome of the bread wheat, including dozens to over two hundred repeat units in some centromeres of this species (Table S4 and Figure S2).

TaeSat16-567 FISH signals were detected in all pericentromeres of the B subgenome (Gálvez-Galván et al. 2024a). This pattern is fully supported by the T2T assembly (Table S4 and Figure S2). Consistent with this, centromeres of the B subgenome contain the highest percentage of satDNA (2.58%), while those from subgenomes A and D have only 0.09% and 0.85%, respectively (Liu et al. 2025). Multiple copies of TaeSat16-567, mostly incomplete, are also scattered throughout the other centromeres except 4D, forming short arrays in several of them (3D and 5D). RepeatMasker reveals sequence variants in these latter loci, due to divergence in the last part of the repetitive unit, where consecutive individual units of ~ 240 bp are separated by ~ 24 bp divergent sequences (Tables S2 and S4).

TaeSat28-543 showed weak FISH signals in all wheat centromeres although strongest signals could be observed in the centromeres of 1B, 1D, 2D, 3B, 4A, 4D and 6D chromosomes (Gálvez-Galván et al. 2024a). The T2T assembled bread wheat genome contains multiple TaeSat28-543 loci located outside the centromeres, close to them in the pericentromeric region, and some loci within centromeres (Figures S1 and S2). However, even though these loci are found in several chromosomes, they are not in all of them, which contradicts FISH results.

SatDNA is the main component of centromeres and pericentromeric heterochromatin in most eukaryotes, particularly animal species, featured by significant sequence diversity and a consequent lack of conservation (reviewed in Garrido-Ramos 2017, 2021). This diversity is even greater in plants, since, in addition to satDNA, the centromeres of many species contain abundant transposable elements (TEs), primarily retrotransposons such as centromeric retrotransposons (CRMs), a specific class of Gypsy elements predominantly found in centromere regions, especially in cereals (Garrido-Ramos 2021). In several cases, TEs have mostly replaced satDNA in centromeres. As in bread wheat, whose centromeres have undergone significant expansion, diversification, and reorganization during evolution and polyploidization. These processes were driven by the activity of retrotransposons from the Gypsy family (especially CRMs and Retand) and by the presence of CACTA elements specific to each subgenome, which has generated complex and differentiated centromeric structures between subgenomes, suggesting that centromeres are critical areas of subgenome differentiation during polyploidization (Li et al. 2022, 2025; Wicker et al. 2018, 2022; Liu et al. 2025) Although the role of satDNA in centromeric function is apparently minor in this species, our results showed differentiated patterns that support the idea that satDNA sequences have undergone rapid changes in wheat subgenomes (Su et al. 2019). Asymmetric centromere organization among the wheat subgenomes may play a role in proper homolog recognition and pairing during meiosis (Su et al. 2019). Interestingly, the uneven chromosomal distribution of satDNAs across eukaryotes generated homologue-specific satDNA “barcodes”, which may facilitate meiotic pairing (Gálvez-Galván et al. 2026; Skrutl et al. 2026). The research conducted in *Drosophila* supports this idea: deletion of satDNA repeats and mismatches in repeat content can compromise meiotic pairing, particularly at centromeres and pericentromeres (Skrutl et al. 2026).

### Subtelomeric satDNAs

**Fig. 7:**
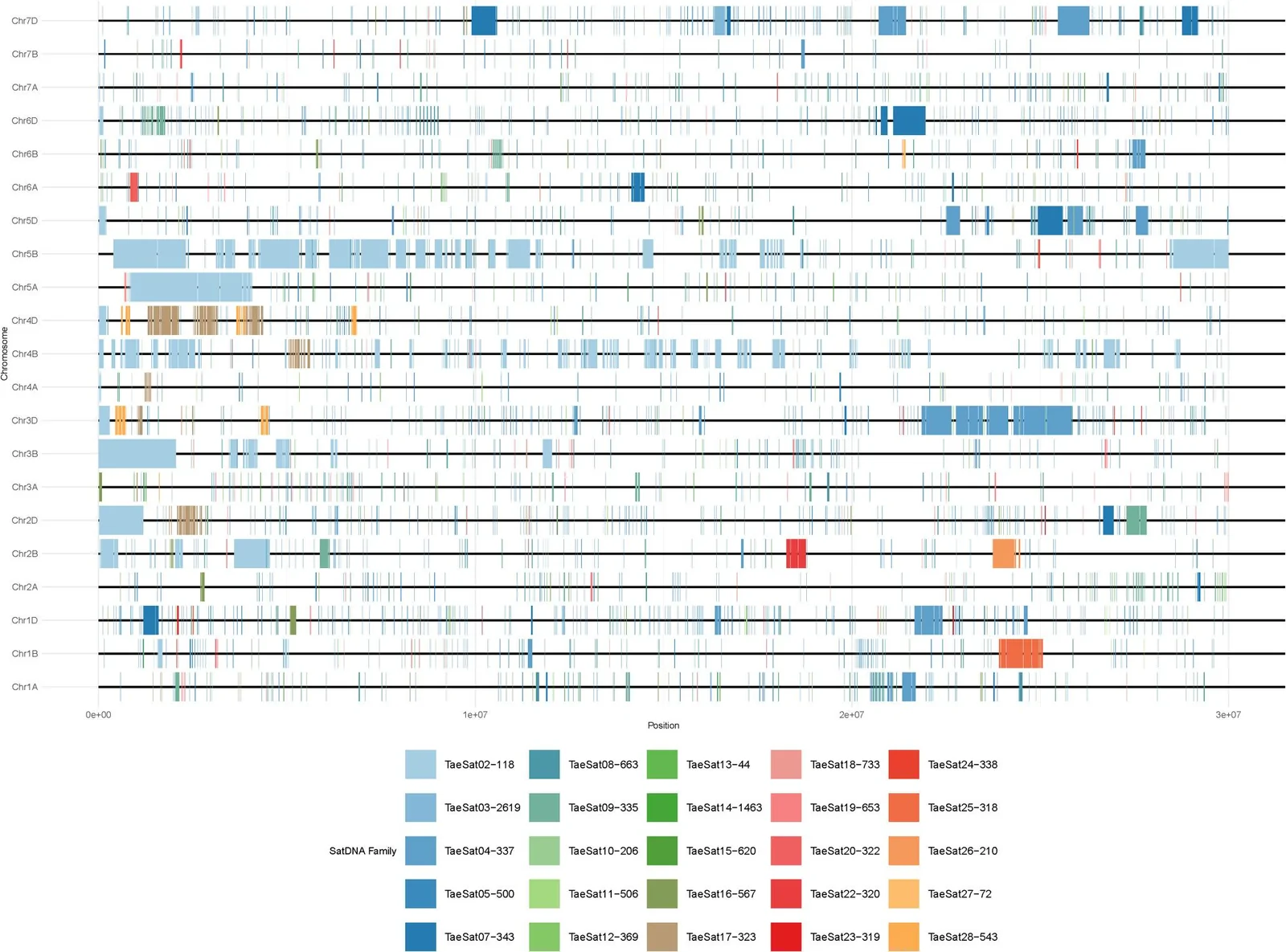
Short arm end (first 30 Mb) of each chromosome of *T. aestivum* cv. Chinese Spring showing the distribution of the different satDNA families as obtained through the CHRISMAPP approach

To date, 26 satellites with distal/subtelomeric localization have been detected in the bread wheat genome (Gálvez-Galván et al. 2024a; Kapustová et al. 2019). Of these, 14 also showed additional FISH signals in pericentromeric and/or interstitial chromosomal regions (Gálvez-Galván et al. 2024a). Bread wheat subtelomeric regions showed high polymorphism in satDNA sequences, reinforcing the idea that these dynamic regions might play a crucial role in chromosome recognition and pairing (Gálvez-Galván et al. 2024a). Indeed, graphical visualization of distribution patterns of the 36 satDNA families across the wheat genome highlights the great diversity that exists at the chromosome ends in the species (last 30 Mb of each end, ~ 5% of the average chromosome length) (Fig. 7 and Figure S10). No two subtelomeric patterns are identical. Furthermore, 15 and 13 satDNAs, respectively, clustered within the most distal region of the chromosomes (the final 1 Mb pair at each chromosome end), have a different organizational pattern (see Table 2). Thus, TaeSat02-118, which appeared in 14 chromosomes, was the most common subtelomeric satDNA. In contrast, TaeSat22-320 and TaeSat25-318 are found only in chromosome 6A. The remaining satellites are present in two to ten different chromosomes (Table 2). Only 6 out of the 15 satDNAs are organized into tandem arrays of certain length, while the remaining 9 satDNAs are found to be isolated monomers or as arrays shorter than ten repeat units. TaeSat04-337 is the most common satDNA at the end of the long arm, appearing in 11 chromosomes. The other 12 satDNAs are found in one to nine chromosomes. As with short-arm end, only 4 out of the 13 satDNAs are organized in tandem arrays of a certain length, while the remaining 9 satDNAs are found as isolated copies or short tandem repeats (dimers to hexamers). Intriguingly, one tandem repeat, homologous to the durum wheat satDNA TtuSat12-178, forms arrays of certain length at the end of the short arm in two chromosomes and at the end of the long arm in three chromosomes (see following).

**Table 2:**
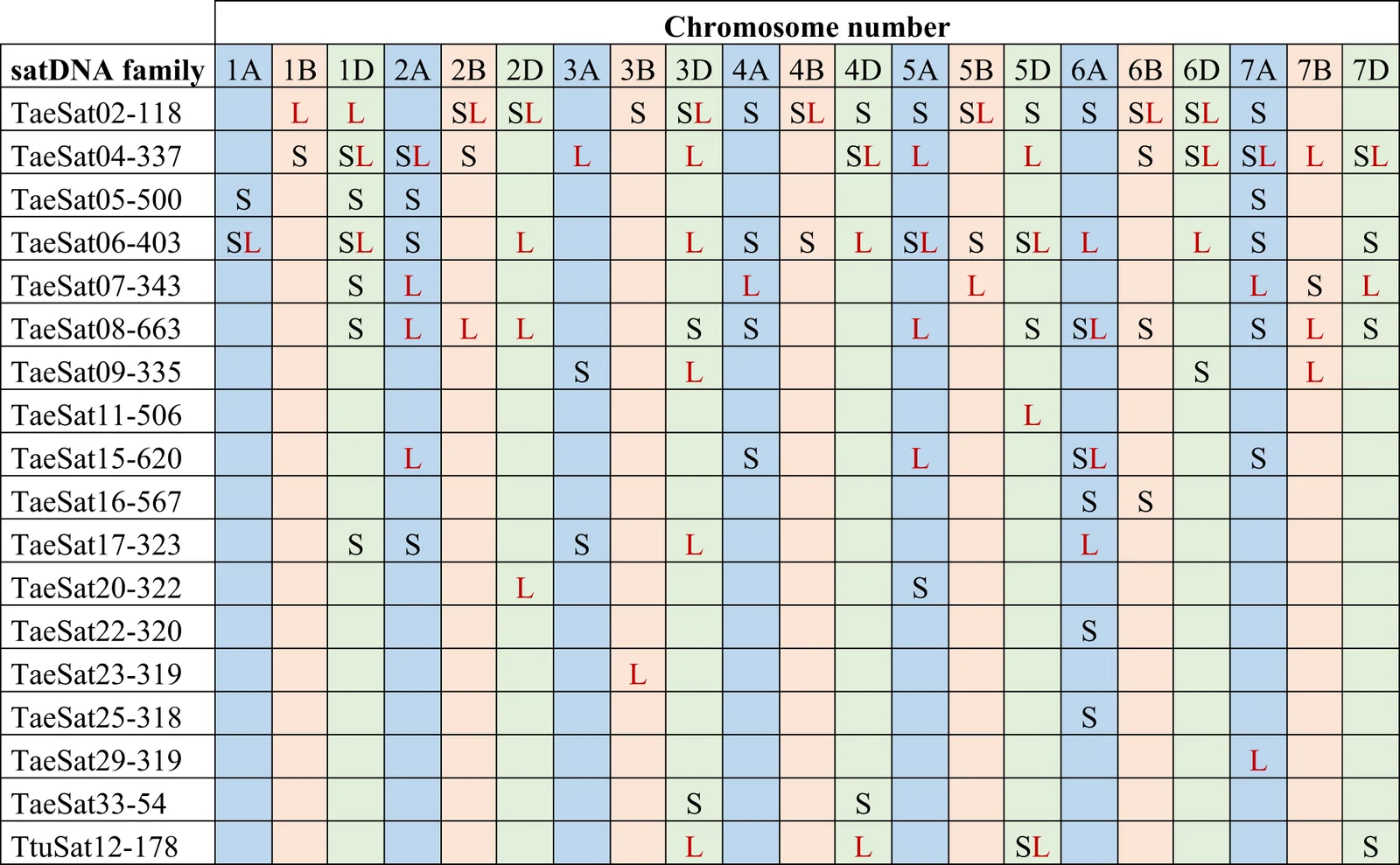
SatDNA repeats clustered in the most distal region of the chromosomes (the final Mb pair at each chromosome end; S: short arm; L: Long arm)

The different subgenomes are differentiated by colour (A = blue; B = pink; D = green)

Taking together, this satDNA organization describes a specific chromosomal pattern in the region closest to the telomeres of both chromosome arms, depending on the particular combination and relative position of the satDNAs (Tables 2 and S2). This supports the proposal that meiotic pairing may be facilitated by satDNA sequences exhibiting chromosome-specific barcode-like profiles (Gálvez-Galván et al. 2026; Skrutl et al. 2026). Specifically, chromosome-specific subtelomeric profiles likely contribute to accurate homolog recognition during meiosis, an essential prerequisite for proper synapsis and recombination (Calderón et al. 2014; Aguilar and Prieto 2020).

### The bizarre case of satellite Ttu12-178

The satDNA Ttu12-178 forms two clear FISH loci in the subtelomeric regions of the short arms of chromosomes 1B and 6B of durum wheat (*T. turgidum*; Gálvez-Galván et al. 2024b). This satellite was also detected after BLAST in the assembled genomes of 23 out of 28 bread wheat accessions, although its abundant varyied greatly depending on the accession (between three and 24,715 hits). However, neither RepeatExplorer nor PCR amplification detected this satellite in *T. aestivum cv.* CS (Gálvez-Galván et al. 2024b). In addition, BLAST search of Ttu12-178 against the CS reference assembled and other five genome assemblies did not yield any results. Therefore, this observation led to the hypothesis that this satDNA might have been lost from the B subgenome of bread wheat due to breeding and artificial selection following the hybridization between *T. turgidum* and *A. tauschii* that resulted in the origin of hexaploid wheat (Gálvez-Galván et al. 2024b).

In this new study, we have traced the presence of TtuSat12-178 in the T2T assembly of CS bread wheat genome, surprisingly with an unexpected distribution. It forms short arrays at various locations on chromosomes 3D, 4D, 5D, and 7D, and very short arrays (< 10 repeat units) also appear on these chromosomes and, additionally in chromosomes 1B (one array of 3 repeat units) and 5A (one array of 4 and another of 2 repeat units). However, the very short arrays in D chromosomes (but not the three loci of 2–4 repeat units of chromosomes 1B and 5A) are longer than shown by RepeatMasker because they are interrupted by short DNA fragments of variable length (in many cases ~ 12 bp), that correspond to length variants of the 178 bp sequence, as observed for several other satDNAs (Table S2).

These data support the idea that TtuSat12-178 has been lost from the B subgenome of CS but remains in the D subgenome. The latter is particularly relevant as the subgenome D is not present in *T. turgidum* (AABB). Consistent with this, a Primer-BLAST search using the primer pair originally designed for TtuSat12-178 satDNA aligned with various locations on different chromosomes (3, 5 and 7) of *A. tauschii* (DD) (Gálvez-Galván et al. 2024b). In this study, BLAST searches against *A. tauschii* genome assemblies in the GrainGenes database (Yao et al. 2025) confirm the presence of homologous sequences on several chromosomes (1, 4, 5, and 7), depending on the accession (Table S5). On the other hand, sequences detected by RepeatMasker in the T2T assembly of the CS bread wheat genome differ substantially (above 20%) from the TtuSat12-178 consensus (Table S2). Furthermore, analysis of these regions using TRF (Benson 1999) shows that the homolog to TtuSat12-178 in *T. aestivum* has a consensus repeat unit of ~ 177 bp and is ~ 23% divergent from the durum wheat consensus sequence (Figure S11). Thus, although the CS repeat and TtuSat12-178 share a common origin, they represent two different satellites, one belonging to D subgenome (related to that found in *A. tauschii* (DD) and present in subgenome D of *T. aestivum* cv. CS) and the other to B subgenome (present in *T. turgidum* but lost in *T. aestivum* cv. CS), which would explain in part, but not entirely, our contradictory results. Therefore, one would expect that the TtuSat12-178 satellite associated to the D subgenome should have been detected by RepeatExplorer in the CS genome. One possible explanation for its absence might be its low proportion in the genome, as it was the case for TaeSat35-1390 and TaeSat36-1242 (Gálvez-Galván et al. 2024a). Further, the divergence between the two satellites (the D- and the B-subgenome versions) could prevent amplification of TtuSat12-178 in CS when using primers designed from the durum wheat satellite.

## Conclusions

The new T2T assembly of the bread wheat genome is highly robust, as it enables the establishment of a complete map of all tandem repeat sequences, an especially complex feature in wheat, as anticipated in Gálvez-Galván et al. (2024a). We have analyzed 36 repetitive sequences that constitute the satellitome of the species and identified the relationship of more than half of them with TEs. We have also proposed a model that explains their origin and evolution based on both TEs and other random genomic sequences. This study completes the map of the location and organization of tandem repetitive sequences in bread wheat, complementing previous analysis of their distribution using FISH. Despite in situ hybridization remains essential for detecting chromosomal markers and much of the information obtained by this procedure is consistent with the distribution of wheat satellites in the genome, the satellitome cannot be fully characterized without the physical mapping provided by the T2T assembly. Factors such as condensation of the mitotic chromosome, the preparation procedures themselves, or the homology between satellites and TEs, can significantly influence some FISH-based observations. This study has also helped clarify some erroneous interpretations obtained previously. We have detected length variants and complex organizations in a significant number of satellites. On the other hand, mapping satDNA sequences in centromeric and distal chromosomal regions reveals both chromosome-specific and subgenome-specific differentiation, which might play a role in the accurate homologous pairing during meiosis, essential for proper synapsis and recombination. In conclusion, these results complete the characterization of the wheat satellitome, providing a comprehensive map of tandem repeated DNA in this species. These data will be useful for researchers interested in furthering the study of the role of these sequences in chromosome architecture, as well as in the organization and function of centromeres and subtelomeres in meiosis.

## Supplementary Information

Below is the link to the electronic supplementary material.

Supplementary Material 1.

Supplementary Material 2.

Supplementary Material 3.

Supplementary Material 4.

Supplementary Material 5.

Supplementary Material 6.

## Data Availability

All raw data underlying this article are available in the article.
