# Supplementary material for: Detailed re-analysis of satellitome mapping facilitated by the telomere-to-telomere (T2T) assembly of bread wheat genome
Source: Plant Mol Biol. 2026 Aug 28;116(5):92. doi: 10.1007/s11103-026-01745-3 (PMC13525055; doi:10.1007/s11103-026-01745-3)
Supplement: Supplementary file 1 [file 11103_2026_1745_MOESM1_ESM.pdf]

Supplementary Information

Detailed re-analysis of satellitome mapping facilitated by the telomere-to-telomere (T2T) assembly of bread wheat genome

Manuel A. Garrido-Ramos and Pilar Prieto

Contents

Supplementary Figures S3 to S11

Appendix 1: Additions to the CHRISMAPP_V2.1.R script (Rico-Porras et al. 2024)

Appendix 2: Custom script for Box plots

Figure S3. Sequence length variants of TaeSat02-118 satDNA family. **A.** The figure shows a short fragment of chromosome 1B (positions 702612108 to 702616097) in which different sequence and sequence length variants of TaeSat02-118 alternate. The successive repeat units that make up this region have been marked with different colors to distinguish each one from the next. The intervening sequence between the array at positions 702612108-702615872 and the first 118-bp repeat unit of the next array, starting at 702615983, is marked in yellow. **B.** Each of the repeat units was numbered consecutively and aligned with the TaeSat02-118 consensus sequence using ClustalX. The figure shows a screenshot of the alignment result for a total of 35 repeat units. As can be seen, there are truncated repeats with a length shorter than the standard length, as well as variants with significant changes at the end of the sequence (sequences 3, 18, and 33).

A.

```
>Chr1B:702612108-702616097

TGTTTTAGGGTCCCAAAGTGATTTCCATGATTGTTGAACCCCAAGGTGCGCTTACGTGT
CGGTCATCAACAATACCAGTTTTGGCCGATTCTGACCCGTTTCATGGACTATTGCTCAC
TATCTTGGGTCCCGGAGCGATTTTCACGATTCACGAACCCCGAGGTGCGTTTATGTGTC
GTCATCAACAATCCGAGTATTTTCGATTTTGGGCTCTTTCATGACCTATTACTCACCAT
TTTGGAGTCCTAGAGCGATTTCCACGATTGATGATCCTCGTGGTGCATTTACGTGTCCG
TCGTCAACACTCAATTTTGGCCGATTCTAGGGTCCCAGGGCGATTTCCACGATTGATGA
ACCCCGGGGTGCGTTTACGTCTCGTTCATCAACAATCCTAGTTTTGTTCTTCTGGGCTC
TTTCATGGTTTATTACTGTCCATTTTAGGGTCCCAGAGCAATTTCCACGATTAACTATC
CCCGGGGTTTGTTTGCGTGTTCGTCCTGAACAGTCATACTTTTGGCCGATTCTGGCCCG
ATTCATAAACTATTACTCACCGTTTTGGGGTCCCAATACGATTATGGTTGTTGAACCCC
TAGGTGCACTTAAGTGTCAGTCATCAATACTCATAGTTTTGACTGATTCTGGCGCGTTT
CTTGGACTATTACTCACTGTTTTGGGGTCCGAGAGAGATTTCCACGATTGAAGAACCCC
GGGGTACGTTTACGTGTCGGTCATCAGCACTCACGGTTTTTGTCGATCCTGACCTATTT
CGTGGACTATTACTCACCGTTTTGGGGTCTGAAAGCAATATCCATGGTTGTTGAACTCC
AAGGAGAGCTTACGTGTCGGTCATCAACAATCGCAGTTTTGGCCGATTATGGCCAGTTT
CTTGGACTATTACCCAGTTTTGGGGTCCCGGAGTGATTTCCACGACTGACGTTCACCGG
GGTGCGTTTTTGTGTTCGTCATCAGCACTCACAGTTTTGGCCGATTCTGGCCCGTTTCA
TGGAGTATTACTCACTGATTTGGGGTCCGTGACCAATTTGCATGATTGACAAACCCCGG
GGTGCGTTTATCTATCGTTCATCAATAATACAAGGTTTGTCCGATTGTGGGCTCTTTCA
TGGACTATTACTCACAGTTTTTGGGTCCCAAAGCGATTTCCATGGTTGTCGAACCCCAA
GGTGTGCTTACATGTTGGTCGTCAAGACTCGCAGTTTGGGCCGATTCTGGCCCGTTTCT
TGGACTATTAGTCACCGTTTTAGGGTTCCAAAGTGATTTCCATGATTGACTAACCCCGA
GGTGCGTTTACGTGTTGGTCATCAACACTCGTTGTTTTTGTCGATTCTGACCCGTTTCG
TGGACTATAACTCACTGTTTTAGGGTCCCAAAGTGATTTCCATGATTGTTGAACCCCAA
GGTGCGCTTATGTGTCGGTCATCAACAATATCAGTTTTGGCCGATTCTGACCCACTTCC
TCGAGTATTACTCAGTTTTTCCCAGAGTGATTTCCACGATTGACGAACCTCGGGGTGCA
TTTATGTGTCAGTCATCAACACTTGCCGTTTTTGTCGATGCTGGCCCGTTTCATGGACT
ATTACTCGCCGTTTTGGGGTCCCGGAGCGATTTCCACGATTGACCAACCCCGAAGTGCG
```

CTTACGTGTCGGTCATCTACAATCACAGTTTTGGCCGATTTCGGCCCATTTCCTGGAAT
ATTACTCAATTTTGGGGCCCCGGAGTGATTTCTACGATTGATGAACACCGGGGTACGTT
TATGTGTCGGTCATCAACACTCGCAGTTTTGGCCGATTCTGGCCCGTTTCATGGACTAT
TGCTCACTATCTTGGGTCCCGGAGCGATTTCCACGATTCACGAACCCCGAGGTGCGTTT
ATGTGTCGTCATCAATAATCCGAGTATTTTAGATTTTGGGCTCTTTCATGACCTATTAC
TCACCACTTTGGAGTCCTAGAGCGATTTCCACGATTGATGATCCTTGTGGTGCGTTTAC
GTGTCCGTCGTCAACACTCAATTTTGGCCGATTCTAGGGTCCCAGGGCGATTTCCACGA
TTGACGAACCCCGGGGTGCGTTTACGTCTCGTTCATCAACAATCCTAGTTTTGTTCTTC
TGGGATCTTTCATGGTTTATTACTGTCCGTTTTAGGGTCCAGGAGCAATTTCCACGATT
AACTATCCCCGGGGTGCGTTTACGTGTTCATCCTAAACAGTCATACTTTTGGCCGATTC
TGGCCCGTTTCATAAACTATTACTCACCATTTTGGGGTCCCAATACGATTGCCATGGTT
GTTGAACCCCTAGGTGCACTTAAGTGTCAGTCATCAATACTCATAGTTTTCACTAATTC
TGGCGCGTTTCTTGGACTATTACTCACTGTTTTGGGGTCCGAGAGAGATTTCCACGATT
GAAGAACCCCGGGGTACGTTTACGTGTCGGTCATCAGCACTCACGGTTTTTGTCGATCC
TGACCTATTTCGTGGACTATTACTCACCGTTTTGGGGTCTGAAAGCAATGTCCATGGTT
GTTGAACTCCAAGGAGAGCTTACGTGTCGGTCATCAACAATCGCAGTTTTGGCCGATTA
TGGCCAGTTTCTTGGACTATTACCCAGTTTTGGGGTCCCGGAGTGATTTCCACGACTGA
CGTTCACCGGGGTGCGTTTTTGAGTTGGTCATCAGCACTCACAGTTTTGGTCGATTCTG
GCCCGTTTCATGGAGTATTACTCACTGATTTGGGGTCCGTGACCAATTTGCATGATTGA
CAAACCCCGGGGTGCGTTTATCTATCGTTCATCAATAATCCAAGGTTTGTCCGATTGTG
GGCTCTTTCATGGACTATTACTCACAGTTTTTGGGTCCCAAAGCGATTTCCATGGTTGT
CGAACCCCAAGGTGTGCTTACATGTTGGTCGTCAAGACTCGCAGTTTGGGCCGATTCTG
GCCCGTTTCTTGGACTATTAGTCACCGTTTTAGGGTCCCAAAGTGATTTCCATGATTGA
CTAACCTCGAGGTGCGTTTACGTGTTGGTCATCAACACTCGTTATTTTTGTCGATTCTG
ACCCGTTTCGTGGACTATAACTCACTGTTTTAGGGTCCCAAAGTGATTTCCATGATTGT
TGAACCCCAAGGTGCGCTTACGTGTCGGTCATCAACAATATCAGTTTTGGCCGATTCTG
ACCCACTTCCTCGAGTATTACTCAGTTTTTCCCAGAGTGATTTCCACGATTGACGAACC
TCGGGGTGCATTTATGTGTCGGTCATCAACACTTGCTCTTTTTGTCTATGCTGGCCCGT
TTCATGGACTATTACTCGCCGTTTTGGGGTCCCGGAGCGATTTCCACGATTGACCAACC
CCGAAGTGCGCTTACGTGTCGGTCATCTACAATCACAGTTTTGGCCGATTTCGGCCCAT
TTCCTGGAATATTACTCAATTTTGGGGCCCCGGAGTGATTTCTACGATTGACGAACACC
GGGGTACGTTTATGTGTCGGTCATCAACACTCGCAGTTTTGGCCGATTCTGGCCCGTTT
CATGGACTATTGCTCACTATCTTGGGTCCCGGAGCGATTTCCACGATTCACGAACCCCG
AGGTGCGTTTATGTGTCGTCATCAACAATCCGAGTATTTTCGATTTTGGGCTCTTTCAT
GACCTATTACTCACCATTTTGGAGTCCTAGAGCGATTTCCACGATTGATGATCCTCGTG
GTGCGTTTACGTGTCCGTCGTCAACACTCAATTTTGGCCGATTCTAGGGTCCCAGGGCG
ATTTCCACGATTGATGAACCCCGGGGTGCGTTTACGTCTCGTTCATCAACAATCCTAGT
TTTGTTCTTCTGGGCTCTTTCATGGTTTATTACTGTCCATTTTAGGGTCCCAGAGCAAT
TTCCACGATTAACTATCCCCGGGGTGCGTTTGCGTGTTCGTCCTGAACAGTCATACTTT
TGGCCGATTCTGGCCCGATTCATAAACTATTACTCA

B.

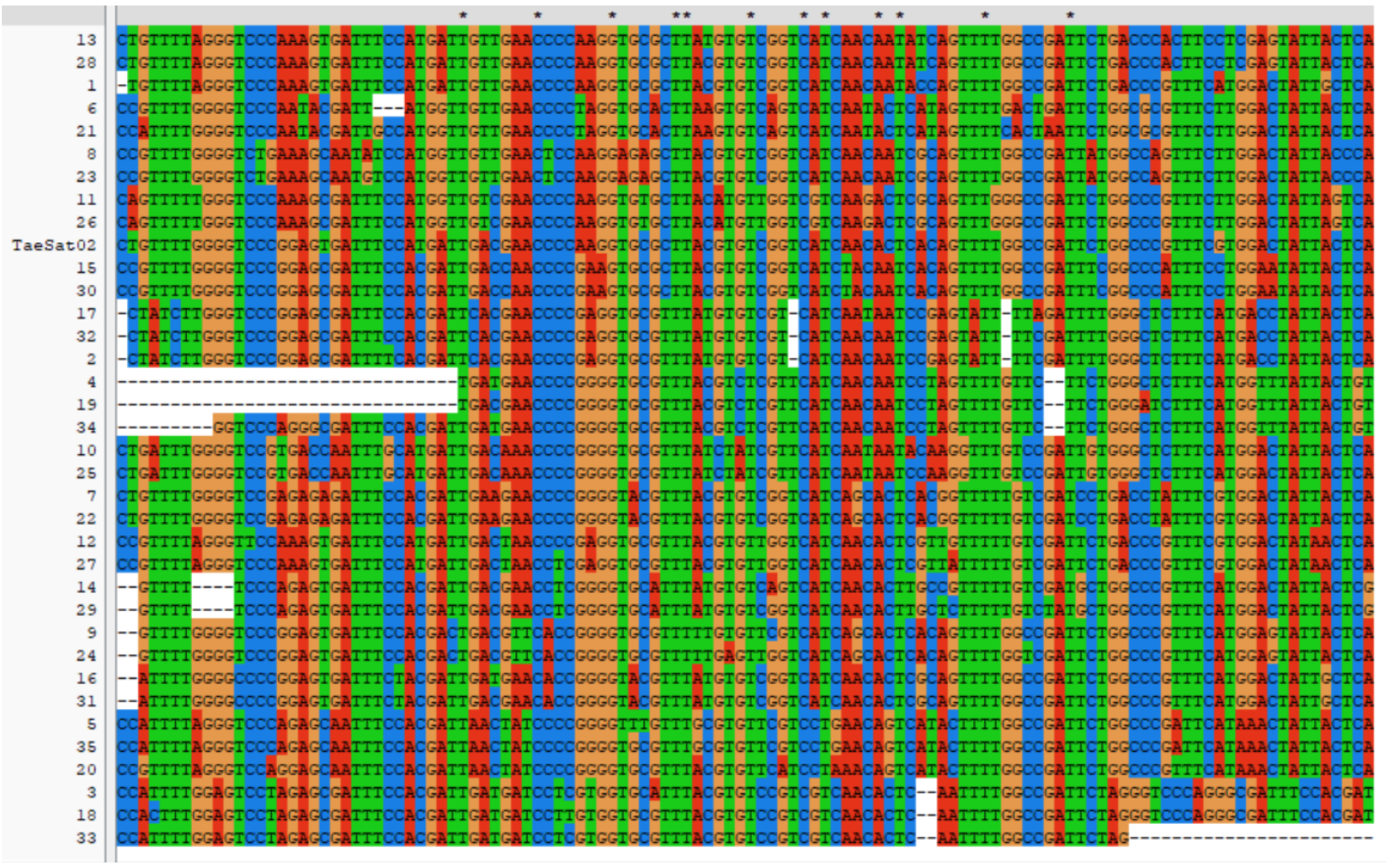
13
28
1
6
21
8
23
11
26
TaeSat02
15
30
17
32
2
4
19
34
10
25
7
22
12
27
14
29
9
24
16
31
5
35
20
3
18
33

Figure S4. Sequence length variants of TaeSat13-44 satDNA family. **A.** TaeSat13-40 and reverse complement of TaeSat13-40 sequence (in the highlighted fragment, the TaeSat13-44 consensus sequence is found in the complementary strand). **B.** Table showing the results of RepeatMasker for region 500734266-500736543 of chromosome 3A of *T. aestivum* cv. CS. **C.** Sequence comprised between 500734266 and 500736543 of chromosome 3A. The successive fragments that make up this region as shown in the table results in B have been marked with different colours to distinguish each one from the next. Furthermore, the boundaries of each repeat unit are marked by the alternation of bold and non-bold letters. Each repeat unit was named and subsequently aligned. **D.** DNA sequence alignment of monomeric units comprised within the region 500734266-500736543 of chromosome 3A.

A.

>TaeSat13-40
GAACTAGCTCTATAAGCTAGTACAAATAATTTTACACTAGAGTT

>Reverse complement of TaeSat13-44
AACTCTAGTGTAAAATTATTTGTACTAGCTTATAGAGCTAGTTC

B.

Query sequence	Position in query begin	end	Array length	RUs	Dist. between arrays
Chr3A	500734266	500734352	86	2,0	88
Chr3A	500734440	500734615	175	4,0	45
Chr3A	500734660	500734923	263	6,0	45
Chr3A	500734968	500735055	87	2,0	52
Chr3A	500735107	500735544	437	9,9	45
Chr3A	500735589	500735938	349	7,9	34
Chr3A	500735972	500736147	175	4,0	45
Chr3A	500736192	500736411	219	5,0	45
Chr3A	500736456	500736543	87	2,0	

C.

>Chr3A:500734266-500736543

ACTCTAGTGTTAAATTATTTGAACTAGCTTGTAGAGCTAGTTCAACTTAGTGTAAAATTATTTGTACT
AGGTTATAGAGGTAGTTC**ATTTCTTGTGTAAACTTATTTGTACTAGGTTATAAAGCTAGTTA**AACTCT
AGTGTAAAATTATTTAATTAGCGTGTAGAGGTAGTTCA**ACTCTTGTGTAAAATTATTTGAACTAGTTT**
CTAGAGCTAGTTCAACTCCAGTGTAAAATTATTTATACTATGTTATAGAGCTAGTTC**AACTCTAGTGT**
AAAATTATTTAAACTAGCCTATAGAGCTAGTTCAACTCTAGTGTAAAATTATTTGCACTAGCTGGTAG
AGCTACTTC**AACTCTGGTGTAAAATTAATTGTACTAGGTTATAGTTCTTGTTC**AACTCTAGTGTAAAA
CTATTTGAACTAGGTTATAGAGCAAGTTC**AACTCTAGTGTAAAATTATTTGACCTAGCCTGTAGAGCT**
AGTTCAACTCTAGTGTAAAATTATTTGTACTAGGTTATAGAGCTAGTTC**AACTCTAGTGTAAAATTAT**

TTAAACTAGCCTGTAGAGCTAGTTTAACTCTAGTGTAAAATTATTTGTTCTAGGTTATAATGCTAGTT
C**AACTCTAGTGTAAAATTATAAGTACTAGGTTATACAACTAGTTC**GAGTCTAGTGCAAAATTATTTAA
CATAGCTTGTAGAGCTAGTTC**AACTCTAGTGTAAAATTATTTGTACTAGGTTATAGAGCTAGTTT**AAC
TCTAGTGTAAAATTATTTAAACTAGCTTGTAGAGCTAGTTC**AACACTAGTGTAAAACTATTTGAACTA**
GCTTGTAGACTGTAGAGCTAGTTCAACTATAGTGCTAAATTATTTGAACTAGCTTGTAGAGCTAGTTC
AATTTAGTGTAAAATTATTTGTACTAGGTTATAGAGCTAGTTCAACTCTAGTGTAAAATTATTTGTAC
TAGGTTATAGATCTAGTTC**AACTCTAGTGTAAAACTGTTTGAACTAGCCTATAGAGCTAGTTG**AACTT
AGTGTAAAATTATTTGAACTAGCTTGTAGAGCTAGTTC**AACTATAGTGTAAAATTGTTTGTACTAGGT**
TATAGAGCTCGTTCAACTCTAGTGTAAAATGATTTGAAATAGCTTGTAGAGCTAGTGA**AACTCTAGTG**
TAAAATTAGTTGAACTAGCTTGTAGAGCTAGTTCAACTCAATTGTAAAATTATTTGTACTAGGTTATG
GAGCTAGTTC**AACTCTAGTGTTAAATTATTTGAACTAGCCTGTAGATCTAGTTC**AACTTTATTTTAAA
ATTATTTGTACTAGGTTATAGAGCTATTTC**AACTCTAGTGTAAAATTATTTGAACTAGCTTGTAGAGC**
TACTTCAACTCTAGTGTTAAATTATTTGTACTAGGTTATAGAGCTAGTTC**AACTCTAGTGTAAAATTA**
TTTGTACTAGGTTATAGAGCTAGTTCAACTCTAGTGTAAAATTATTTGTACTAGGTTATAGAGCTTGT
TC**AGCTCTAGTGTAAAATTATTTGTGCTAGGTTATAGAGCTAGTTC**AACTCTAGTGTAAAATTATTTG
TGCTAGGTTATAGAGCTAGTTC**AACTCTAGTGTAAAATTATTTGTACTAGGTTATAGATCTAGTTC**AA
CTCTAGTGTAAAATTATTTGTACTAGGTTATAGAGCTAGT**AAAATTATTTGTACTAGGTTATAGAGCT**
AGTTTAACTCTAGTGTAAAATTATTTGTACTGGGTTATATTGCTAGTTC**AACTCTAGTGTAAAATTAT**
TTGTGCTAGGTTGTAGAGCTAGTTCAACTCTAGTGTAAAATTCTTTGTACTAGGTTATAGAACTAGTT
C**AACTCTAGTGTAAAATTATTTGACCTAGCCTGTAGAGCTACTTC**AACTCTAGTGTAAAACAATTTGT
ACTAGGTTACAGATCAAGTTC**AACTCTAGTGTAAAATTATTTGTACTAGTTTATAGAGCTAGTTC**AAC
TCTAATGTAAAATTATTTGAACTAGCCTGTAAAGCTAGTTT**AACTCTAGTGTAAAATTATTTGAACTA**
GCTTGTAGAGCTAGTTCAACTCTAGTGTAAAATTATTTGTACTAGGTTATAGTGGTAGTAC**AACTCTA**
GTGTAAAATTATTAGTACTAGGTTATACAACTAGTTCGACTCTAGTTCAAAATTATTTGACATAGATT
GTAGAGCTTGTTC**AACTCTAGTGTAAAATTATTTGTACTGGGTTATAGAGCTAGTTC**AACTCTAGTGT
AAAATTATTTAAACTAGCTTGTAGAGCTAGTTC

D.

```
86_1            -ACTCTAGTGTTAAATTATTTGAACTAGC-------TTGTAGAGCTAGTTC
437_1           AACTATAGTGCTAAATTATTTGAACTAGC-------TTGTAGAGCTAGTTC
437_X           AACTCTAGTGTTAAATTATTTGAACTAGC-------CTGTAGATCTAGTTC
263_2           AACTCTAGTGTAAAATTATTTGACCTAGC-------CTGTAGAGCTAGTTC
175b_4          AACTCTAGTGTAAAATTATTTGACCTAGC-------CTGTAGAGCTACTTC
263_4           AACTCTAGTGTAAAATTATTTAAACTAGC-------CTGTAGAGCTAGTTT
219_2           AACTCTAATGTAAAATTATTTGAACTAGC-------CTGTAAAGCTAGTTT
52              AACACTAGTGTAAAACTATTTGAACTAGCTTGTAGACTGTAGAGCTAGTTC
437_4           AACTCTAGTGTAAAACTGTTTGAACTAGC-------CTATAGAGCTAGTTG
86_2            AACT-TAGTGTAAAATTATTTGTACTAGG-------TTATAGAGGTAGTTC
219_4           AACTCTAGTGTAAAATTATTTGTACTAGG-------TTATAGTGGTAGTAC
263_5           AACTCTAGTGTAAAATTATTTGTTCTAGG-------TTATAATGCTAGTTC
175b_1          AACTCTAGTGTAAAATTATTTGTACTGGG-------TTATATTGCTAGTTC
87b_1           AACTCTAGTGTAAAATTATTTGTACTGGG-------TTATAGAGCTAGTTC
45a             AACTCTGGTGTAAAATTAATTGTACTAGG-------TTATAGTTCTTGTTC
349_4           AACTCTAGTGTAAAATTATTTGTACTAGG-------TTATAGAGCTTGTTC
437_6           AACTATAGTGTAAAATTGTTTGTACTAGG-------TTATAGAGCTCGTTC
263_1           AACTCTAGTGTAAAACTATTTGAACTAGG-------TTATAGAGCAAGTTC
45d             AACTCTAGTGTAAAACAATTTGTACTAGG-------TTACAGATCAAGTTC
437_3           AACTCTAGTGTAAAATTATTTGTACTAGG-------TTATAGATCTAGTTC
349_7           AACTCTAGTGTAAAATTATTTGTACTAGG-------TTATAGATCTAGTTC
263_6           AACTCTAGTGTAAAATTATAAGTACTAGG-------TTATACAACTAGTTC
219_5           AACTCTAGTGTAAAATTATTAGTACTAGG-------TTATACAACTAGTTC
175b_3          AACTCTAGTGTAAAATTCTTTGTACTAGG-------TTATAGAACTAGTTC
88_1            ATTTCTTGTGTAAACTTATTTGTACTAGG-------TTATAAAGCTAGTTA
34              -----------AAAATTATTTGTACTAGG-------TTATAGAGCTAGTTT
437_2           -AATTTAGTGTAAAATTATTTGTACTAGG-------TTATAGAGCTAGTTC
87a_1           AACTCTAGTGTAAAATTATTTGTACTAGG-------TTATAGAGCTAGTTT
349_8           AACTCTAGTGTAAAATTATTTGTACTAGG-------TTATAGAGCTAGT--
349_3           AACTCTAGTGTAAAATTATTTGTACTAGG-------TTATAGAGCTAGTTC
263_3           AACTCTAGTGTAAAATTATTTGTACTAGG-------TTATAGAGCTAGTTC
349_2           AACTCTAGTGTTAAATTATTTGTACTAGG-------TTATAGAGCTAGTTC
349_5           AGCTCTAGTGTAAAATTATTTGTGCTAGG-------TTATAGAGCTAGTTC
349_6           AACTCTAGTGTAAAATTATTTGTGCTAGG-------TTATAGAGCTAGTTC
175b_2          AACTCTAGTGTAAAATTATTTGTGCTAGG-------TTGTAGAGCTAGTTC
437_9           AACTCAATTGTAAAATTATTTGTACTAGG-------TTATGGAGCTAGTTC
45c             AACTTTATTTTAAAATTATTTGTACTAGG-------TTATAGAGCTATTTC
175_2           AACTCCAGTGTAAAATTATTTATACTATG-------TTATAGAGCTAGTTC
219_1           AACTCTAGTGTAAAATTATTTGTACTAGT-------TTATAGAGCTAGTTC
175_1           AACTCTTGTGTAAAATTATTTGAACTAGT-------TTCTAGAGCTAGTTC
175_3           AACTCTAGTGTAAAATTATTTAAACTAGC-------CTATAGAGCTAGTTC
175_4           AACTCTAGTGTAAAATTATTTGCACTAGC-------TGGTAGAGCTACTTC
349_1           AACTCTAGTGTAAAATTATTTGAACTAGC-------TTGTAGAGCTACTTC
437_5           AACT-TAGTGTAAAATTATTTGAACTAGC-------TTGTAGAGCTAGTTC
219_3           AACTCTAGTGTAAAATTATTTGAACTAGC-------TTGTAGAGCTAGTTC
437_8           AACTCTAGTGTAAAATTAGTTGAACTAGC-------TTGTAGAGCTAGTTC
87a_2           AACTCTAGTGTAAAATTATTTAAACTAGC-------TTGTAGAGCTAGTTC
87b_2           AACTCTAGTGTAAAATTATTTAAACTAGC-------TTGTAGAGCTAGTTC
45b             GAGTCTAGTGCAAAATTATTTAACATAGC-------TTGTAGAGCTAGTTC
45e             GACTCTAGTTCAAAATTATTTGACATAGA-------TTGTAGAGCTTGTTC
437_7           AACTCTAGTGTAAAATGATTTGAAATAGC-------TTGTAGAGCTAGTGA
88_2            AACTCTAGTGTAAAATTATTTAAT-TAGC-------GTGTAGAGGTAGTTC
```

Figure S5. Sequence length variants of TaeSat09-335 satDNA family. **A.** TaeSat09-335 sequence (according to Gálvez-Galván et al. 2024a). **B.** Example of sequence variants in the region 2047786 to 2050118 of chromosome 1A [Green: one incomplete TaeSat09-335 repeat unit (330 bp); Red: one new 1000 bp repeat unit composed of two consecutive incomplete 335-bp repeat units (331 bp and 334 bp) plus a new 335-bp related repeat (different shades of red indicate the three different parts); Blue: one new 1002 bp repeat unit composed of two consecutive incomplete 335-bp repeat units (333 bp and 333 bp) plus a new 335-bp related repeat (different shades of blue indicate the three different parts)]. **C.** Alignment of two 1001-bp sequences.

A.

>TaeSat09-335
GTCTCCGGGGTGTGGCCCCCCTCACGGACGGCGCCGCCGCCGGCACCTGGAGGGGGGAAACGGACGCGTG
GTAACTACACGGAGGGGATCTTGCTGTGGGTCTGGAGCGCGTGTTGCTCCAAAGTGTTCCTATGGGCTGA
CCTAACACAACCGGGTGGAGAGGTTTTTACTGGTCAAAGCCCATCCGTGCAAAGTCAAAGGGCTAGATCC
CGTGGTCAAACGCTACAGGGTTAGGCGGGACGGGGGCCTCGGGGGGCTAGAATGCCACCGGAGCGTCGGT
AGGCGCCCTCATACATGCGGGGTGGTGTTGTGGCATGTCCGAAGAGGCGGCACGC

B.

>Chr1A:2047786-2050118

CTCCGGGGTGTGGCCCCGTCACGGACGGCGCCGCCGCCGGCACCTGGAGGGGGGGAACGGACGCGTCGGG
TCTACATGGAGTGGATGTATCTGTGGGTTTGGCCCGGATGTTGCTCCCGGGTGTCCTTATGGGCGGACCT
GACACAACCGGGTGGAGAGGTCCACTGGTCAAAGCCCGTCCGTGCAAAGTCAAAGGGCTAGATCCCGTGG
TCAAACGCTACAGGGTTAGCCGGGACGGGGGCCCTGGGGGGTAGCAATGCCACCGAAGCGTCGTACCGGG
CCCTCATACATGCGGGGTGGTGTTGTGGCATGTCCGAAGAGGCGGCACGCGTCTCCGGGGTGTGGCCCCC
GTCACGGACGGCGCCGACGCCGGCACCTGGAGGGGGAAACGGACGCGTCGGGTCTACGCGGAGAGGATCT
TGCTGTGGGTCTGGCCCGGATGTTGCTCCCCGGTGTCCCTTTGGGCTGACCTGACACAACCGGGTGGAGA
GGTCCACTGGTCAAAGCCCATCCGTGCAAAGTCAAAGGGCTAGATCCCGTGGTCAACCGCTCCAGGGTTA
GGCAGGACGGGGGCCCTGGGGGTAGCAATGCCACCGGAGCGTCGTACCGGGCCCTCATACATGCGGGGTG
GTGTTGTGCAATGTCCGAAGAGGCGACACGCATCTTCGGGGTGTGCCCCCCATCACGGATGGCGCCGCTG
CCGTCACCTGGAGGGGGGAAAACGGACGCGTCGGGTCTACACGGAGTGGATGTAGCTGTGGGTTTGGCCC
GGATGTTGCTCCCGGGTGTCCCTATGGGTAGACCTGACACAACCGGGTGGAGAGGTCCACTGGTCAGAGC
CCGTCCGTGGGAAGTCAAAGGGCTAGATCTCGTGATCAACCGCTCCTGGGTTAGGCGGGACGGGGACCCT
GGGGGGTAGCAATGCCACCGGAGCATCGCACCGGGCCCTCATACATGCGGGGTGGTGTGGTGGCATGTCC
GAAGAGGCGGCACGCATCTCCGGGGCGTGGCCCCCCCTAACGGACGGCGATGACATGGTAGTAGCCATTC
TGCGGTAACGATGCGGCGTGAATATTATTAAATACTCCGAGCGCGATAAAATCATGGGTTTTTTTGCACG
ACGTGGGCACATGTGCTATAGGAACGATAATATTTTTTCATAATTTTTGGTGATGGAGAAGTATCCCAAA
AATTCCCTCAACGTGGATTGCCCTTCGCGCGTCTATGGCTGTGGCCGGCGGCCTCCGGGGGCTAGCATGC
CGCCAAATCTTGCGTAGGCGGCCTCTCACAAGTTTGCAAGTGTTGTGGTGGTTGCAAACGTCTGGCACGC
GTGTCCGGGGTGTGGCCCCCCTCACGGACGGTTCCGCCGCCGACACTTGGAGGGGGGAAACGGACGCGTC
GGGTGTACACGGAGGGGATCTTGTTGTGGGTTTGGCCCGGATGTTGCTCCCAGGTGTCCCTCTGAGCTGA
CCTGACACAACCGGGTGGAGAGGTCCCCGGTTCAAAGCCTGTCCGTGGAAAGTCAGAGGGCAAGATCTCG
TGGTCAACCGCTCTAGGGTTAGGCAGGACAGGGGCCCGGGGGGGTAGCAATGCCACCGGAGCGTCGCACC
GGGCCCTCATACATGCGGGGTGGTGTGGTGGCATGTCCGAAGAGGCGGCACGCGTCTCCGGGGTTTGGCC
CCCCTCACGGATGGCGCCACCGCCGGCACCTGGAGGGGGGAAACGGACGCGTCGGGTCTACACGGATGGG
ATCTTGCTGTGGGTCTGGCCCGGATGTTGCTCCAAAGTGTTCCTATGGGCTGACCTAACACAACCGGGTG
GAGAGGTCCACTGGTCAAATCCCGTCCGTGTAAAGTAAAAGGGCTAGATCCCGTGGTCAAACGGTACAGG
GTTAGGCGGGACGGGGGCCCGGGGGGGTAGCAATGCCACCGGAGCATCGCACCGGGCCCTCATACATGCG
GGGTGGTGTGGTGGCTTGTCCGAAGAGGCAGCACGCATCTCCGGGGCATGGCCCCCCCTAACGGACGGCG
ATGACACGGTAGTAGCCATTCTGCGGTAACGATGCGGCGTGAATATTATTAAATACTTGGAGCGCGATAA

```
AATCATGAGTTTTTTTGCACGACGTGCGCACATGTGCTATAGGAACGATAATATTTTTTCATAATTTTTG
GTGATGGAGAAGTATCCCAAAAATTCCCTCTACGCGGATTGCCATTCGCTCGTCTACGGCTGTGGCCGGT
GGCCTCCGGGGGCTAGCATGCCGCCAAATCTTGCGTAGGCGGCGTCTCACAAGTTTGGAAGTGTTTTGGT
GGTTGCAAACGCCCGGCACGCG
```

C.

```
Trimer1         GTCTCCGGGGTGTGGCCCCCGTCACGGACGGCGCCGACGCCGGCACCTGGAGGGGG-AAA
Trimer2         GTGTCCGGGGTGTGGCCCCCCTCACGGACGGTTCCGCCGCCGACACTTGGAGGGGGGAAA
                 ** ***************** **********  *** ***** *** ********* ***

Trimer1         CGGACGCGTCGGGTCTACGCGGAGAGGATCTTGCTGTGGGTCTGGCCCGGATGTTGCTCC
Trimer2         CGGACGCGTCGGGTGTACACGGAGGGGATCTTGTTGTGGGTTTGGCCCGGATGTTGCTCC
                 ************** *** ***** ******** ******* ******************

Trimer1         CCGGTGTCCCTTTGGGCTGACCTGACACAACCGGGTGGAGAGGTCCACTGGTCAAAGCCC
Trimer2         CAGGTGTCCCTCTGAGCTGACCTGACACAACCGGGTGGAGAGGTCCCCGGTTCAAAGCCT
                 * ********* ** ******************************* * * ********

Trimer1         ATCCGTGCAAAGTCAAAGGGCTAGATCCCGTGGTCAACCGCTCCAGGGTTAGGCAGGACG
Trimer2         GTCCGTGGAAAGTCAGAGGGCAAGATCTCGTGGTCAACCGCTCTAGGGTTAGGCAGGACA
                  ****** ******* ***** ***** *************** ***************

Trimer1         GGGGCCCTGGGGG-TAGCAATGCCACCGGAGCGTCGTACCGGGCCCTCATACATGCGGGG
Trimer2         GGGGCCCGGGGGGGTAGCAATGCCACCGGAGCGTCGCACCGGGCCCTCATACATGCGGGG
                 ******* ***** ********************** ***********************

Trimer1         TGGTGTTGTGCAATGTCCGAAGAGGCGACACGCATCTTCGGGGTGTGCCCCCCATCACGG
Trimer2         TGGTGTGGTGGCATGTCCGAAGAGGCGGCACGCGTCTCCGGGGTTTGGCCCCCCTCACGG
                 ****** ***  *************** ***** *** ****** ** ***** ******

Trimer1         ATGGCGCCGCTGCCGTCACCTGGAGGGGGGAAAACGGACGCGTCGGGTCTACACGGAGTG
Trimer2         ATGGCGCCACCGCCGGCACCTGGAGGGGGGAAA-CGGACGCGTCGGGTCTACACGGATGG
                 ******** * **** ***************** ***********************  *

Trimer1         GATGTAGCTGTGGGTTTGGCCCGGATGTTGCTCCCGGGTGTCCCTATGGGTAGACCTGAC
Trimer2         GATCTTGCTGTGGGTCTGGCCCGGATGTTGCTCCAAAGTGTTCCTATGGGCTGACCTAAC
                 *** * ********* ******************   **** ********  ***** **

Trimer1         ACAACCGGGTGGAGAGGTCCACTGGTCAGAGCCCGTCCGTGGGAAGTCAAAGGGCTAGAT
Trimer2         ACAACCGGGTGGAGAGGTCCACTGGTCAAATCCCGTCCGTGTAAAGTAAAAGGGCTAGAT
                 **************************** * **********  **** ************

Trimer1         CTCGTGATCAACCGCTCCTGGGTTAGGCGGGACGGGGACCCTGGGGGGTAGCAATGCCAC
Trimer2         CCCGTGGTCAAACGGTACAGGGTTAGGCGGGACGGGGGCCCGGGGGGGTAGCAATGCCAC
                 * **** **** ** * * ****************** *** ******************

Trimer1         CGGAGCATCGCACCGGGCCCTCATACATGCGGGGTGGTGTGGTGGCATGTCCGAAGAGGC
Trimer2         CGGAGCATCGCACCGGGCCCTCATACATGCGGGGTGGTGTGGTGGCTTGTCCGAAGAGGC
                 ********************************************** *************

Trimer1         GGCACGCATCTCCGGGGCGTGGCCCCCCCTAACGGACGGCGATGACATGGTAGTAGCCAT
Trimer2         AGCACGCATCTCCGGGGCATGGCCCCCCCTAACGGACGGCGATGACACGGTAGTAGCCAT
                  ***************** **************************** ************

Trimer1         TCTGCGGTAACGATGCGGCGTGAATATTATTAAATACTCCGAGCGCGATAAAATCATGGG
Trimer2         TCTGCGGTAACGATGCGGCGTGAATATTATTAAATACTTGGAGCGCGATAAAATCATGAG
                 **************************************  ****************** *

Trimer1         TTTTTTTGCACGACGTGGGCACATGTGCTATAGGAACGATAATATTTTTTCATAATTTTT
Trimer2         TTTTTTTGCACGACGTGCGCACATGTGCTATAGGAACGATAATATTTTTTCATAATTTTT
                 ***************** ******************************************

Trimer1         GGTGATGGAGAAGTATCCCAAAAATTCCCTCAACGTGGATTGCCCTTCGCGCGTCTATGG
Trimer2         GGTGATGGAGAAGTATCCCAAAAATTCCCTCTACGCGGATTGCCATTCGCTCGTCTACGG
                 ******************************* *** ******** ***** ****** **
```

```
Trimer1         CTGTGGCCGGCGGCCTCCGGGGGCTAGCATGCCGCCAAATCTTGCGTAGGCGGCCTCTCA
Trimer2         CTGTGGCCGGTGGCCTCCGGGGGCTAGCATGCCGCCAAATCTTGCGTAGGCGGCGTCTCA
                ********** ******************************************* *****

Trimer1         CAAGTTTGCAAGTGTTGTGGTGGTTGCAAACGTCTGGCACGC-
Trimer2         CAAGTTTGGAAGTGTTTTGGTGGTTGCAAACGCCCGGCACGCG
                ******** ******* *************** * ******* 
```

Figure S6. Sequence length variants of TaeSat26-210 satDNA family. **A.** TaeSat26-210 sequence (according to Gálvez-Galván et al. 2024a). **B.** Sequence length variant (362 bp). Inserted sequence in boldface. **C.** Alignment of TaeSat26-210 length variants between positions in chromosome 1B. **D.** Organization of those sequence variants in the indicated region. See the sequential organization of 210bp-362bp-362bp-210bp-362bp-362bp-362bp repeat units. The end of each repeat unit is marked yellow.

A.

>TaeSat26-210
TTCCTTTAGTTTGCTCATTCGCTTAATTAGTTACATCGAAGATCTAGACAGGAGGTCCTGTGTTCAATTCCCACAATGTTGATTTATTTTGACCCAATTA
TTTTTCGCGGTCTCTATGAAAGCCCATAAAAGGCCCATCAACATAGAAGTACCTGGCCCAGATTGACCAAACGAAAAGGACTAAACCAAACCAAGAAAAG
GACGCAATCC

B.

> TaeSat26-210 length variant (362 bp)
TTCCTTTAGTTTGCTCATTCGCTTAATTAGTTACATCGAAGATCTAGACAGGAGGTCCTGTGTTCAATTCCCACAATGTTGATTTATTTTGACCCAATTA
TTTTTCGCGGTCTCTATGAAAGCCCATAAAAGGCCCATCAACATAGAAGTACCTGGCCCAGATTGACCAAACGAAAAGGACTAAACCAAACCAAGAATA**C**
CTATTCCCTTTAATAGTAGGATAATTCCTTTTTTAATTTAGTAGCCACAACACATTGAAGCCTATGGATCCTTCCTTTCAATATAGAAATCAACATAGAA
GTACCTGGCCCAGATTGACCAAACGAAAAGGACTAAACCAAACCAAGAAAAGGACGCAATCC

C.

TTCCTTTAGTTTGCTCATTCGCTTAATTAGTTACGTCGAAGATCTAGACAGGAGGTCCTGTGTTCAATTCCCACAATGTTGATTTATTTTGACCCAA
TTCCTTTAGTTTGCTCATTCGCTTAATTAGTTACATCGAAGATCTAGACAGGAGGTCCTGTGTTCAATTCCCACAATGTTGATTTATTTTGACCCAA
TTCCTTTAGTTTGCTCATTCTCTTAATTAGTTACATCGAAGATCTAGACAGGAGGTCCTGTGTGCAATTCCCACAATGTTGATTTATTTTGACCCAA
TTCCTTTAGTTTGCTCATTCGCTTAATTAGTTACATCGAAGATCTAGACAGGAGGTCCTGTGTTCAATTCCCACAATGTTGATTTATTTTGACCCAA
TTCCTTTAGTTTGCTCATTCGCTTAATTAGTTACGTCGAAGATCTAGACAGGAGGTCCTGTGTTCAATTCCCACAATGTTGATTTATTTTGACCCAA
TTCCTTTAGTTTGCTCATTCGCTTAATTAGTTACATCGAAGATCTAGACAGGAGGTCCTGTGTTCAATTCCCACAATGTTGATTTATTTTGACCCAA
TTCCTTTAGTTTGCTCATTCGCTTAATTAGTTACATCGAAGATCTTGACAGGAGGTCTTGTGTTCAATTCCCAAAATGTTGATTTATATTGACCCAA

TTATTTTTCGCGGTCTCTATGAAAGCCCATAAAAGGCCCATCAACATAGAAGTACCTGGCCCAGATTGACCAAACGAAAAGGACTAAACCAAACCAA
TTATTTTTCGCGGTCTCTATGAAAGCCCATAAAAGGCCCATCAACATAGAAGTACCTGGCCCAGATTGACCAAACGAAAAGGACTAAACCAAACCAA
CTATTTTTCGCGGTCTCTATGAAAGCCCATAAAAGGCCCATCAACATAGAAGTACCTGGCCCAGATTGACCAAACGAAAAGGACTAAACCAAACCAA
TTATTTTTCACGGTCTCTATGAAAGCCCATAAAAGGCCCATCAACGTAGAAGTACCTGGCCCAGATTGACCAAACGAAAAGGACTAAACCAAACCAA
TTATTTTTTGCGGTCTCTATGAAAGCCCATAAAAGGCCCATCAACATAGAAGTACCTGGCCCAGATTGACCAAACGAAAAGGACTAAACCAAACCAA
TTATTTTTCGCGGTCTCTATGAAAGCCCATAAAAGGCCCATCAACATAGAAGTACCTGGCCCAGATTGACCAAACGAAAAGGACTAAACCAAACCAA
TTATTTTTCGCGGTCTCTATGAAAGCCCATAAAAGGCCCATCAACATAGAAGTACCTGGCCCAGATTGACCAAACGAAAAGGACTAAACGAAACCAA

GAAAA * GGACGCAATCC
GAATA * GGACGCAATCC
GAATA * GGACGCAATCC
GAATA * GGACGCAATCC
GAAAA * GGACGCAATCC
GAATA * GGATGCAATCC
GAATA * GGACGCAATCC

***Nothing**
***CCTATTCCCTTTAATAGTAGGATAATTCCTTTTTTAATTTAGTAGCCACAACACATTGAAGCCTATGGATCCTTCCTTTCAATATAGAAATCAACAT**
AGAAGTACCTGGCCCAGATTGACCAAACGAAAAGGACTAAACCAAACCAAGAAAA
***CCTATTCCCTTTAATAGTAGGATAATTCCTTTTATAATTAAGTAGCCCCAACACATTGAAGCCTATGGATCCTTCCTTCCAATATAGAAATCAACAT**
AGAAGTACCTAGCCCAGATTGACCAAATGAAAATGACTAAACAAACCAAGAAAA
***CCTATTCCCTTCAATAGTAGGATAATTCCTTTTTTAATTTAGTAGCCCCAACACATTGAAGCCTATGGATCCTTCCTTTCAATATAGAAATCAACAT**
AGAAGTACCTGGCCCAGATTGACCAAACGAAAAGGACTAAACCAAACCAAGAAAA
***Nothing**
***CCTATTCCCTTTAATAGTAGGATAATTCCTTTTTTAATTTAGTAGCCACAACACATTGAAGCCTATGGATCCTTCCTTTCAATATAGAAATCAACAT**
AGAAGTACATGGCCCAGATTGACCAAACGAAAAGGACTAAACCAAACCAAGAAAAA
***CCTATTCCCTTTAATAGTAGGATAATTCCTTTTTTAATTTAGTAGCCCCAACACATTGAAGCCTATGGATCCTTCCTTTCAATATAGAAATCAACAT**
AGAAGTACCTGGCCCAGATTGACCAAACGAAAAGGACTAAACCAAACCAAGAAAA

D.

>Chr1B:23920612-23923566

TTCCTTTAGTTTGCTCATTCGCTTAATTAGTTACGTCGAAGATCTAGACAGGAGGTCCTGTGTTCAATTCCCACAATGTTGATTTATTTTGACCCAATTA
TTTTTCGCGGTCTCTATGAAAGCCCATAAAAGGCCCATCAACATAGAAGTACCTGGCCCAGATTGACCAAACGAAAAGGACTAAACCAAACCAAGAAAAG
GACGCAATCCTTCCTTTAGTTTGCTCATTCGCTTAATTAGTTACATCGAAGATCTAGACAGGAGGTCCTGTGTTCAATTCCCACAATGTTGATTTATTTT
GACCCAATTATTTTTCGCGGTCTCTATGAAAGCCCATAAAAGGCCCATCAACATAGAAGTACCTGGCCCAGATTGACCAAACGAAAAGGACTAAACCAAA
CCAAGAATA**CCTATTCCCTTTAATAGTAGGATAATTCCTTTTTTAATTTAGTAGCCACAACACATTGAAGCCTATGGATCCTTCCTTTCAATATAGAAAT**
CAACATAGAAGTACCTGGCCCAGATTGACCAAACGAAAAGGACTAAACCAAACCAAGAAAAGGACGCAATCCTTCCTTTAGTTTGCTCATTCTCTTAATT
AGTTACATCGAAGATCTAGACAGGAGGTCCTGTGTGCAATTCCCACAATGTTGATTTATTTTGACCCAACTATTTTTCGCGGTCTCTATGAAAGCCCATA
AAAGGCCCATCAACATAGAAGTACCTGGCCCAGATTGACCAAACGAAAAGGACTAAACCAAACCAAGAATA**CCTATTCCCTTTAATAGTAGGATAATTCC**
TTTTATAATTAAGTAGCCCCAACACATTGAAGCCTATGGATCCTTCCTTCCAATATAGAAATCAACATAGAAGTACCTAGCCCAGATTGACCAAATGAAA
ATGACTAAACAAACCAAGAAAAGGACGCAATCCTTCCTTTAGTTTGCTCATTCGCTTAATTAGTTACATCGAAGATCTAGACAGGAGGTCCTGTGTTCAA
TTCCCACAATGTTGATTTATTTTGACCCAATTATTTTTCACGGTCTCTATGAAAGCCCATAAAAGGCCCATCAACGTAGAAGTACCTGGCCCAGATTGAC
CAAACGAAAAGGACTAAACCAAACCAAGAATA**CCTATTCCCTTCAATAGTAGGATAATTCCTTTTTTAATTTAGTAGCCCCAACACATTGAAGCCTATGG**
ATCCTTCCTTTCAATATAGAAATCAACATAGAAGTACCTGGCCCAGATTGACCAAACGAAAAGGACTAAACCAAACCAAGAAAAGGACGCAATCC**TTCCT**
TTAGTTTGCTCATTCGCTTAATTAGTTACGTCGAAGATCTAGACAGGAGGTCCTGTGTTCAATTCCCACAATGTTGATTTATTTTGACCCAATTATTTTT
TGCGGTCTCTATGAAAGCCCATAAAAGGCCCATCAACATAGAAGTACCTGGCCCAGATTGACCAAACGAAAAGGACTAAACCAAACCAAGAAAAGGACGC
AATCCTTCCTTTAGTTTGCTCATTCGCTTAATTAGTTACATCGAAGATCTAGACAGGAGGTCCTGTGTTCAATTCCCACAATGTTGATTTATTTTGACCC
AATTATTTTTCGCGGTCTCTATGAAAGCCCATAAAAGGCCCATCAACATAGAAGTACCTGGCCCAGATTGACCAAACGAAAAGGACTAAACCAAACCAAG
AATA**CCTATTCCCTTTAATAGTAGGATAATTCCTTTTTTAATTTAGTAGCCACAACACATTGAAGCCTATGGATCCTTCCTTTCAATATAGAAATCAACA**
TAGAAGTACATGGCCCAGATTGACCAAACGAAAAGGACTAAACCAAACCAAGAAAAAGGATGCAATCCTTCCTTTAGTTTGCTCATTCGCTTAATTAGTT
ACATCGAAGATCTAGACAGGAGGTCCTGTGTGCAATTCCCACAATGTTGATTTATTTTGACCCAACTATTTTTCGCGGTCTCTATGAAAGCCCATAAAAG
GCCCATCAACATAGAAGTACCTGGCCCAGATTGACCAAACGAAAAGGACTAAACCAAACCAAGAATA**CCTATTCCCTTTAATAGTAGGATAATTCCTTTT**
ATAATTAAGTAGCCCCAACACATTGAAGCCTATGGATCCTTCCTTCCAATATAGAAATCAACATAGAAGTACCTAGCCCAGATTGACCAAATGAAAAGGA
CTAAACCAAACCAAGAAAAGGATGCAATCCTTCCTTTAGTTTGCTCATTCGCTTAATTAGTTACATCGAAGATCTAGACAGGAGGTCCTGTGTTCAATTC
CCACAATGTTGATTTATTTTGACCCAATTATTTTTCACGGTCTCTATGAAAGCCCATAAAAGGCCCATCAACATAGAAGTACCTGGCCCAGATTGACCAA
ACGAAAAGGACTAAACCAAACCAAGAATA**CCTATTCCCTTTAATAGTAGGATAATTCCTTTTTTAATTTAGTAGCCCCAACACATTGAAGCCTATGGATC**
CTTCCTTTGAATGTAGAAATCAACATAGAAGTACCTGGCCCAGATTGAGCAAACCAAAAGGACTAAACCAATCCAAGAAAAGGACGCAATCCTTCCTTTA
GTTTGCTCATTCGCTTAATTAGTTACATCGAAGATCTTGACAGGAGGTCTTGTGTTCAATTCCCAAAATGTTGATTTATATTGACCCAATTATTTTTCGC
GGTCTCTATGAAAGCCCATAAAAGGCCCATCAACATAGAAGTACCTGGCCCAGATTGACCAAACGAAAAGGACTAAACGAAACCAAGAATA**CCTATTCCC**
TTTAATAGTAGGATAATTCCTTTTTTAATTTAGTAGCCCCAACACATTGAAGCCTATGGATCCTTCCTTTCAATATAGAAATCAACATAGAAGTACCTGG
CCCAGATTGACCAAACGAAAAGGACTAAACCAAACCAAGAAAAGGACGCAATCC

Figure S7. Sequence length variants of TaeSat30-1389 satDNA family. **A.** Segment comprised between 513963894 and 513970617 sites in chromosome 5A. We show four consecutive repeat units of ~1,681 bp, composed of one 1,389 unit (1,383 bp because of a variable number of indels) (red) plus a sequence segment of ~298 bp (blue). **B.** Segment comprised between 414364174 and 414372598 sites in chromosome 5D. We show five consecutive repeat units of ~1,682-1,688 bp, composed of one 1,389 unit (1,386-1,391 bp because of a variable number of indels) (red) plus a sequence segment of ~296-298 bp (blue).

A.

>Chr5A: 513963894-513970617

TTGGAGATGAGCATCACTGGCAATCCTCGTCCGATCCCACATGATGTGAGGTGCTCTGGCGTTG
CCAATATGATACTGGAGCTTGGCAAGAAGGCGGAGGAAAAAGGACAAAATCATAACTGATGCAA
AGTTGCTAGCAGGGAACTAGTAGGCGTTGGGGCCAATATTCGTAAATAGTGAAATAAAGCCACT
TAGCTATTTGTTGTTCTGATGTATGAATGTTATAATGATTTGTGATGTATGCGTTTGTATTGTA
CTGCATTGTGCACGTGTGTGGTAACCATGTGCTCAAACAAATTATTTTTACCATTATTTTATTG
AGCTTTCCTTTTCTTCGGGAACTTGTTCAAACTTGCTTTTGGGTGGAATTACACTTATGTGCAT
ATGAGTGGGTTACATTTTCCCAAAATTCTAACAATTTTTGGAAGGTTGCTTATTATTGCTAATT
TAGCAATTGCATTCCGGTTTATCTTGATAGGTTTTGCGAGTGGGGAATTTTGGAGCTTGAATTT
CAAAATCAAAAACCATAGTTCAAAGTTTGAAAAAAATCATACGTTTTTATACATATGTTCATAT
GGATGCATTCCACAACTGTATTATGTTTCACGATGAAATATGTTAACATGTGAGCTACACAAAA
ATGAAAATTTTGTGAATTTTTTAATAATTAACAATGCATGTACTGTTCACCATTATCAGACTTA
TGATTTTTTTTGTAGTTCACTAGAAGTGTGGCAAATTTTGGAAAGTTATTTTTATATGAGTTCG
ACATTTCCAATACTCATCATGTTTAAACCTTTTGCAGAAGAGAAGATGACAACACAAGTGTTGT
CAATAATGGAGAAATGTGAGGAACCTAGCTCAAATATAATGAAAGTTGCCAATCTTACTTTCTC
AGGCGATAATGGCAAAAAGATGAGCACTAACATGATGGGATTGAACTTGAAGGAGGCACTTTCC
CAACGCGATAAATGCATGGTAAGAAACCTTGTGCTGAACGGATGCATTAAATTTTTAAATGCAA
TATGTATCTTTGACACCTGTCAATGGAGGAGCCAGATGAGATCTTCTGTGATTGTGATTGGATG
GCCCCTAGATACACAGTAGTAGCTTCACTAGCACACGGTATGTGTAAATACATTTATCTTAGCT
CTTTAAATTTCCAAATGGACAAGCATTGGGTTCATGGACCATATAGGCACAATAATAAAGAATT
CCATACAAATGGGTCTTGTGCCACGTACTTTCTGAATAGCGTTTATTGAATCCAATCCCTATTG
TGTTCCCTTAGTATCACGCATTGAACTGTTAGCATACATGGTGTACTATGCTTAGCATGGTTGG
GCCAAAGAATTTTCATGTGGTAAGATCTATAAACCACTTGTCTATGCCCTGTTCAGGTGTTAGC
TTCAATCAACGTCACGGTTCGATCCATGGTTACCAAAGACATGTTTTGCGCCATTGTACGATCT
ATAAGAGATAACCTCTACTCACTTCATTGCATTCCACTTGTTAAGAATAGAATAGGTCTCCACC
TAGCAATCACCAGGAACTGTTGTTTGATTTAGTGCCATCTTCTCTCATTCATAGTTACAGATTG
TTTTTCGAACACGGGTTGACTAGTCAATCTCCTATCTTTTGAGGAATGGACGTCGGTGAAGTAC
GTTTTAAATTCCCCGATTTGGAGATGAGCATCACTGGCAATCCTCGTCCGATCCCACATGATGT
GAGGTGCTCTGGCGCTGCCAATATGATACTGGAGCTTGGCAAGAAGGCGGAGGAAAAAGGACAA
AATGATAACTGATGCAAAGTTGCTAGCAGGGAACTAGTAGGCGTCGGGGCCAATATTCGTAAAT
AGTGAAATAAAGCCACTTAGCTATTGGTTGTTCTGATGTATGAATGTTATAATGATTTGTGATG
TATGCGTTTGTATTGTACAACATTGTGCACGTGTGTGCTAACCATGTGCTCAAACAAATTATTT
TTACCATTATTTTATTGAGTTTTCCTTTTCTTCGGGAACTTGTTCAAACTTGCTTTTGGGTGGA
ACTACACTTATGTGCATATGAGTGGGTTACATTTTCCCAAAATTCTAACACTTTTTGGAAGGTT
GCTTATTATTGCTAATTTAGCAATTGCATTCCTGTTTATCTTGATAGGTTTTGCGAGTGGGGAA
TTTTGGAGCTTGCATTTCAAAATCAAAAACCATAGTTCAAAGTTTGAAAAAAATCATACGTTTT
TATACATATGTTCATATGGATGCATTCCACAACTGTATTATGTTTCACGATGAAATATGTTAAC
ATGTGAGCTACACAAAAATGAAAATTTTGTGAATTTTTTAATAATTACCAATGCATGTACTGTT

CACCATTATCAGACTTATGATTTTTTTTGTAGTTCACTAGAAGTGTGGCAAATTTTGGAAAGTT
ATTTTTATATGAGTTCGACATTTCCAATACACATCATGTTTAAACCTTTTGCAGAAGAGAAGAT
GACAACACTAGTGTTGTCAATAATGGAGAAATGTGTGGAACCTAGCTCAAATATAATGAAAGTT
GCCAATCTTACTTTCTCAGGCGATAATGGCAAAAACATGAGCACTAACATGATGGGATTGAACT
TGAAGGAGGCACTTTCCCAACGTGATAAATGCATGGTAAGAAACCTTGTGCTGAACGGATGCAT
TAAATTTTTAAATGCAATATGTATCTTTGACACCTGTCAATGGAGGAGCCAGATGAGATCTTCT
GTGATTGTGATTGGATGGCCCCTAGATACATAGTAGTACCTTCACTAGCACCCGGTATGTGTAA
ATACATTTATCTTAGCTCTTTAAATTTCCAAATGGACAAGCATTGGGTTCATGGACCATATAGG
CACAATAATAAAGAATTCCATACAAATGGGTCTTGTGCCACGTACTTTCTGATTAGCGTTTATT
GAATCCAATCCCTATAGTGTTCCCTTAGTATCAAGCATTGAACTGTTAGCATACATGGTGTACT
ATGCTTAGCATGCTTGGGACAATGAATTTTCATGTGGTAAGATCTATAAACCACTTGTGTATGC
CCTGTTCAGGTGTTAGCTTTAATCAACGTCACGGTTCGATCCATGGTTACCAAAGACATGTTTT
GCGCCATTGTACGATCTATAAGAGATAACCTCTACTCACTTCATTGCATTCCACTTGTTAAGAA
TTGAATAGGTCTCCACCTAGCAATCACCAGGAACTGTTGTTTGATTTAGTGTCATCTTCTCTCA
TTCATAGTTACAGATTGTTTTTCGAACACGGGTTGACTAGTCAATCTCCTATCTTTTGATGAAT
GGACGTCGGTGAAGTACATTTTAAATTCCCCGATTTGGAGATGAGCATCACTGGCAATCCTCGT
CCGATCCCACATGATGCGAGGTGCTCTGGCGTTGCCAATATGATACTAGAGCTTGGCAAGAAGG
CGGAGGAAAAAGGACAAAATGATAACTGATGCAAAGTTGCTAGCAGGTAACTAGTAGGCGTTGG
GGCCAATATTCGTAAATAGTGAATTAAAGCCACTTAGCTATTTGTTGTTCTGATGTATGAATGT
TATAATGATTTGTGATGTATACGTTTGTATTGTACTGCATTGTGCACGTGTGTGCTAACCATGT
GCTCAAACAAATTATTTTTACCATTATTTTATTGAGCTTTCCTTTTCTTCGGGAACTTGTTCAA
ACTTGCTTTTGGGTGGAATTACACTTATGTGCATATGAGTGGGTTACATTTTCCCAAAATTCTA
ACAATTTTTGGAAGGTTGCTTATTATTGCTAATTTAGCAATTGCATTCCGGTTTATCTTGATAG
GTTTTGCGAGTGGGGAATTTTGGAGCTTGAATTTCAAAATCAAAAACCATAGTTCAAAGTTTGA
AAAAAATCATACGTTTTTATACATATGTTCATATGGATGCATTCCACAACTGTATTATGTTTCA
CGATGAAATATGTTAACATGTGAGCTACACAAAAATGAAAATTTTGTGAATTTTTTAATAATTA
ACAATGCATGTACTGTTCACCATTATCAGACTTATGATTTTTTTTGTAGTTCACTAGAAGTGTG
GCAAATTTTGGAAAGTTATTTTTATATGAGTTCGACATTTCCAATACTCATCATGTTTAAACCT
TTTGCAGAAGAGAATATGACAACACAAGTGTTGTCAATAATGGAGAAATGTGAGGAACCTAGCT
CAAATATAATGAAAGTTGCCAATCTTACTTTCTCAGGCGATAATGGGAAAAAGATGAGCACTAA
CATGATGGGATTGAACTTGAAGGAGGCACTTTCCCAACGCGATAAATGCATGGTAAGAAACCTT
GTGCTGAACGGATGCATTAAATTTTTAAATGCAATATGTATCTTTGACACCTGTCAATGGAGGA
GCCAGATGAGATCTTCTGTGATTGTGATTGGATGGCCCCTAGATACACAGTAGTACCTTCACTA
GCACACGGTATGTGTAAATACATTTATCTTAGCTCTTTAAATTTCCAAATGGACAAGCATTGGG
TTCATGGACCATATAGGCACAATAATAAAGAATTCCATATAAATGGGTCTTGTGCCACGTACTT
TCTGATTAGCGTTTATTGAATCCAATCCCTATAGTGTTCCCTTAGTATCACGCATTGAACTATT
AGCATACATGGTGTACTATGCTTAGCATGGTTGGGCCAAAGAATTTTCATGTGGTAAGATCTAT
AAACCACTTGTCTATGCCCTGTTCAGGTGTTAGCTTCAATCAACGTCACGGTTCGATCCATGGT
TACCAAAGACATGTTTTGCGCCATTGTACGATCTATAAGAGATAACCTCTACTCACTTCATTGC
ATTCCACTTGTTAAGAATAGAATAGGTCTCCACCTAGCAATCACCAGGAACTGTTGTTTGATTT
AGTGCCATCTTCTCTCATTCATAGTTACAGATTGTTTTTCGAACACGGGTTGACTAGTCAATCT
CCTATCTTTTGAGGAATGGACGTCGGTGAAGTACGTTTTAAATTCCCCGATTTGGAGATGAGCA
TCACTGGCAATCCTCGTCCGATCCCACATGATGTGAGGTGCTCTGGCGCTGCCAATATGATACT
GGAGCTTGGCAAGAAGGCGGAGGAAAAAGGACAAAATGATAACTGATGCAAAGTTGCTAGCAGG
GAACTAGTAGGCGTTGGGGCCAATATTCGTAAATAGTGAAATAAAGCCACTTAGCTATTTGTTG
TTCTGATGTATGAATGTTATAATGATTTGTGATGTATGCGTTTGTATTGTACTGCATTGTGCAC
GTGTGTGCTAACCATGTGCTCAAACGAATTATTTTTACCATTATTTTATTGAGTTTTCCTTTTC
TTTGGGAACTTGTTCAAACTTGCTTTTGGGTGGAACTACACTTATGTGCATATGAGTGGGTTAC
ATTTTCCCAAAATTCTAACACTTTTTGGAAGGTTGCTTATTATTGCTAATTTAGCAATTGCATT
CCTGTTTATCTTGATAGGTTTTGCGAGTGGGGAATTTTGGAGCTTGCATTTCAAAATCAAAAAC
CATAGTTCAAAGTTTGAAAAAAATCATACGTTTTTATACATATGTTCATATGGATGCATTCCAC
AACTGTATTATGTTTCACGATGAAATATGTTAACATGTGAGCTACACAAAAATGAAAATTTTGT

GAATTTTTTAATAACTACCAATGCATGTACTGTTCACCATTATCAGACTTATGATTTTTTTTGT
AGTTCACTAGAAGTGTGGCAAATTTTGGAAAGTTATTTTTATATGAGTTCGACATTTCCAATAC
TCATCATGTTTAAACCTTTTGCAGAAGAGAAGATGACAACACTAGTGTTGTCAATAATGGAGAA
ATGTGAGGAACCTAGCTCAAATATAATGAAAGTTGCCAATCTTACTTTCTCAGGCGATAATGGC
AAAAACATGAGCACTAACATGATGGGGTTGAACTTGAAGGAGGCACTTTCCCAACGTGATAAAT
GCATGGTAAGAAACCTTGTGCTGAACGGATGCATTAAATTTTTAAATGCAATATGTATCTTTGA
CACCTGTCAATGGAGGAGCCAAATGAGATCTTCTGTGATTCTGATTGGATGGCCCCTAGATACA
CAGTAGTACCTTCACTAGCACATGGTATGTGTAAATACATTTATCTTAGCTCTTTAAATTTCCA
AATGGACAAGCATTGGGTTCATGGACCATATAGGCACAATAATAAAGAATTCCATACAAATGGG
TCTTGTGCCACGTACTTTCGGATTAGCGTTTATTGAATCCAATCCCTATAGTGTTCCCTTAGTA
TCAAGCATTGAGCTGTTAGCATACATGGTGTACTATGCTTAGCATGCTTGGGACAATGAATTTT
CATGTGGTAAGATCTATAAACCACTTGTCTATGCCCTGTTCAGGTGTTAGCTTTAATCAACGTC
ATGGTTCGATCCATGGTTACCAAAGACATGTTTTGCGCCATTGTACGATCTATAAGAGATAACC
TCTACTCACTTCATTGCATTCCACTTGTTAAGAATTGAATAGGTCTCCACCTAGCAATCACCAG
GAACTGTTGTTTGATTTAGTGTCATCTTCTCTCATTCATAGTTACAGATTGTTTTTCGAACACG
GGTTGACTAGTCAATCTCCTATCTTTTGATGAATGGACGTCGGCGAAGTACATTTTAAATTCCC
CGAT

B.

>Chr5D:414364174-414372598

TTTGAGATGAGCATCACTGGCGATCCTCATCTGACCCCACATGATGCGAGATGCTCTACAGCTG
CCAATATGATATTGGAGTTTGGGAAGAAGGCGGAGGGAAAAGGACGAAATGAGAACTGATGCAA
AGTTGGTAGCTGGGAACTAGTAGGCGCTGGGGCCAATATTCGTAAAGAGTGAGATAAAGCCACT
TAGCTATTTGTAGTTCTAATGTATGAATGTTATAATGATTTGTGTTGTATGAGTTTGTATTGTA
TTGCGTTGTGCACGTGCGTGCTAACCATGTGCTCAGATGAATTATTTTTACCATTATTTTATTG
AGTTTTCCTTTTTGGGGGAACTTGTTCAAACTTGCTTTTGGGTGGAATTTCACTTATGTGCATA
TGAGTGGGTTACATTTTCCCAAAATTCTAGCAATTTTTGGAAGGTTGCTTATTATTGCTAATTT
AGCAATTGCATTCCTGTTTATCTTGATAGGTTTTGCGAGTGAGGCATTTTGGAGCTTGAATATT
AAAAATATCAAAACCCATAGTTTGAATTTTGAAAAAATCATAGGGTCTTATACATATGTTCATA
TGGATGCATTCCACAAGTGTATAATGTTTCACGATGAAATATGTTAACATGTGAGCTAGACAAA
AATGAAAATTTTGTGAATTTTTAATAGTGAACAATGCATGTACAGTTCACCATTATCAAAGTTA
TGAATTTGTTTTTCTTTGTAGTTCACTAGAAGTGTGGCAAATTTTGGCAAGTTATTTCGATATG
AGTTCCACATTTCCATTACTCATCATGTTTAATTCTTATGAAGAAGGGGACAGCAGAAGTGTTG
TCAATAATGGAGAAGTATAAGGAACCTAGCTCAAATATAATGGAAGCTGCCAATCTAACTTTCT
CAGGCGACGATGGCAAAACCAACAGCACTAACAGGATGGGATTGAACATGAAGGAGGCACTTTC
CCAACGCCATAAATGCATGGTAAGAAACCTTGTGCTGAACAGATGCATTGAATTTTTAAATGCA
ATCTATATCTTTGACACTTATCAATGCTGGAGCTTGATGAGATATGCTGTGATTGTGATTGGAT
GGCCCCTACATACATAGTAGTACATTCACTAGTAGATTGTATATGTAAGTACATTTACTCTTAT
CTCTTTAATTTTCCAAATGGACAAGCATTGGGTTCATGGACCATACAGGCACAATAATAAAGAA
TTCCATACAAATGGGTCTTCTGCCACATAATTCCAGACTAGCGTTTATTGAATCGAATCTCTAT
AATGTCCCCTTAGTATCATGCATTGAACTGTTAGCTTACATGGTGTACTATGGTTAGCATGCCT
AGGAAAAAGAATTTCCTAGTGGTCAGATATATAAACTACTTTTCTATGCCCTGTTCAGGTGTTA
GGTTTATTCAACATCACGGTTTGGTCCATGGTTACCAAAGACATGCGTCGCGCAATTGTATGGT
CTATAAGAAATCACCTCGACTAGCTTCATTGCATTCCACTTGTCAAGAACTGAGCAGGTCTCCA
CCTTGCAATAACCAGGAACTGTTTTTGGTTTTCATGGCATCTTCTCACATTCATAGTCACATAT
TGTTTTTTGAGAACTTATTCATCAGTCAATATCCTATCTTTTGAGGAATGCACGTCGGCGAAGT
ATGTTTGAAATGCCCTGATTTATTGATGAGCATCACTGGCGATCCTCGTCCGACCCCACATGAT
ACAAGGAGCTCTAGAGCTGCCAATATGATACCGGAGCTTAGCAAGAAGGCGCATGAAAAAGGAC

AAAATTATAACTGATGCATAGTTGCTAGCTGGGAACTAGTAGGCGCTCGGGCGAATATTCATAA
AGAGTGAAATAAAGCGACTTAGCTATTTGTTGTTCTGATGTATGAGTGTTATAATGATTTGTGT
TGTATGAGTTTGTATTATATTGCGTTGTGGACGTCTGTGCTAACCATGTGCTCAAACGAATTAT
TTTTACTATTATTTTATTGAGTTTTCCTGTTTTGGCGGAACATGTTCGAACTTGCTTTTTGGTG
GAATTTCACTTATGTGCATACGAGTGGGTTACATTTTCCCAAAATAGTAGCACTTTTCGGAAGG
TTGCTTATTATTGCTAATTTAGCAATTGCATTCTTGTTTATCTTGATAGGTTTTGCGAGTGAGG
GATTTTGGAGCTTGAATTTTAAAAATATCAAAAACCATAGTTTGAATTTCAAAAAATCATCGGT
TTTTACACATATGTTCATATAGATGCATTCCACAAGTGTATAATGTTTCACGATGAGATATGTT
AACATGTGAGCTGCACAAAAATGAAAATTTTTGTGAATTTTTAATAGTGAACAATGCATGTACT
GTTCACCATTATTAAAGCGTGAATTCTTTTTTCTTTGTAGTTCACTAGAAGTGTGGCAAATTTT
GGCAAGTTATTTCTATATGAGTTCGACATTTCCATTACTCATCATGTTTAAGCATAATGCAGAA
GAGAAGATGACAGCAGAAGTGTTGTCAATAATGGAGAAATATAAGGAACCTAGCTCAAATATAA
TGAAAGCTGCCAATCTAACTTTCTCCGGCGATGATGGCAAAACCAAGAGCACTAGCAGGATGGG
AATTAACTTGAAGGAGGCACTTTCCCAACGCGATAAATGCATGGTAAGAAACCTTGTACTAAAC
GGATGCATTGAAATTTTAAATGCAATCTATATCTTTGACACTTGTCAATGCAGGAGCTTGATGA
CATATGGTGTTATTGTGATTGGATGGCCCCTAGATACACAGTAGTACCTTCACTAGCATATGGT
ATGTGTAAGTACATTCGCTCTTATCTCTTTAATTTTCCAAATGGACAAGCATTGGGTTCATGGA
CCATGTAGGCACAATAATAAAGAATTCCATACAAATGGGTCTTGGGCCACGTAATTCATGAGTT
GCGTTTATTGAATGCAATTCCTATAGTGTCCCCTTAGTATCATGCACTGAAGTATTAGCATACA
TGGTGTACTATGCTTAGCATGCTTGGGAAAAAGAATTTCCATGTGATCAGATCTATAAACCACA
TGTCTATTCCCTTTTCAGGTGTTAGGTTTATTCAACGTCACAGTTCGGTCCATGGTTACCAAAG
ACATGTGTTGCGCCATTGTACGTTCTATAAGAGATCACCTCTACTCACTTCATTGCATTCAACT
TGTCAAGAACTTAATAGGTCTCCACCGAGCAATAGCTAGGAAGTGATGTTTGTTTTAGTGCCAT
CTTCTCTCATTCATAGTCATAGAATGTTTTTCGAGCACTTATTCACTAGTCAATCCCCTATCTT
TTCAGGAATGCACATCGACGAAGTATGTTCGAAATGCGCTGATTTTGAGATGAGCATCACTGGC
GATCCTCATCTGACCCCACATGATGCGAGGTGCTCTACAGCTGCCAATATGATATTGGAGCTTG
GGAAGAAGGCGGAGGGAAAAGGACGAAATGATAACTGATGCAAAGTTGGTAGCTGGGAACTAGT
AAGCGCTGGGGCCAATATTCGTAAAGAGTGAGATAAAGCCACTTAGCTATTTGTTGTTCTAATG
TATGAATGTTATAATGATTTGTGTTGTATGAGTTTGTATTGTATTGCGTTGTGCACGTGCGTGC
TAACCATGTGCTCAAATGAATTATTTTTACCATTATTTTATTGAGTTTTCCTTTTTGGGGGAAC
TTGTTCAAACGTGCTTTTGGGTGGAATTTCACTTATGTGCATATGAGTGGGTTACATTTTCCCA
AAATTCTAGCAATTTTTGGAAGGTTGCTTATTATTGCTAATTTAGCAATTGCATTCCTGTTTAT
CTTGATAGGTTTTGCGAGTGAGGCATTTTGGAGCTTGAATATTAAAAATATCAAAACCCATAGT
TTGAATTTTGAAAAAATCATAGGTTCTCATACATATGTTCATATGGATGCATTCCACATGTGTA
TAATGTTTCACGATGAAATATGTTAACATGTGAGCTAGACAAAAATGAAAATTTTGTGAATTTT
TAATAGTGAACAATGCATGTACAGTTCACCATTATCAAAGTTATGAATTTGTTTTTCTTTGTAG
TTCACTAGAAGTGTGGCAAATTTTGGCAAGTTATTTCGATATGAGTTCCACATTTCCATTACTC
ATCATGTTTAATTCTTATGAAGAAGGGGACAGCAGAAGTGTTGTCAATAATGGAGAAGTATAAG
GAACCTAGCTCAAATATAATGGAAGCTGCCAATCTAACTTTCTCAGGCGACGATGGCAAAACCA
AGAGCACTAACAGGATGGGATTGAACTTGAAGGAGGCACTTTCCCAACGCCATAAATGCATGGT
AAGAAACCTTGTGCTGAATAGATGCATTGAATTTTTAAATGCAATCTATATCTTTGACACTTAT
CAATGCTGGAGCTTGATGAGATATGCTGTGATTGTGATTGGATGGCCCCTACATACATAGTAGT
ACATTCACTAGTAGATTGTATATGTAAGTACATTTACTCTTATCTCTTTAATTTTCCAAATGGA
CAAGCATTGGGTTCATGGACCATACAGGCACAATAATAAAGAATTCCATACAAATGGGTCTTCT
GCCACATAATTCCAGACTAGCGTTTATTGAATCGAATCTCTATAATGTCCCCTTAGTATCATGC
ATTGAACTGTTAGCTTACATGGTGTACTATGGTTAGCATGCCCAGGAAAAATAATTTCCTAGTG
GTCAGATATATAAACTACTTTTCTATGCCCTGTTCAGGTGTTAGGTTTATTCAACATCACGGTT
CGGTCCATGGTTACCAAAGACATGCGTCGCGCCATTGTATGGTCTATAAGAGATCTCCTCGACT
AACTTCATTGCATTCCACTTGTCAAGAACTGAGCAGGTCTCCACCTTGCAATAACCAGGAACTG
TTTTAGGTTTTCATGGCATCTTCTCACATTCATAGTCACATATTGTTTTTGAGACCTTATTCAT
CAGTCAATATCCTATCTTTTGAGGAATGCACGTCGGCGAAGTATGTTTGAAATGCCCTGATTTA
TTGATGAGCATCACTGGCGATCCTCGTCCGACCCCACATGATGCAAGGTGCTCTAGAGCTGCCA

ATATGATACTGGAGCTTAGCAAGAAGGCGCATGAAAAAGGACAAAATTATAACTGATGCATAGT
TGCTAGCTGGGAACTAGTAGGCGCTCGGGCGAATATTCATAAAGAGTGAAATAAAGCGACTTAG
CTATTTGTTGTTCTGATGTATGAGTGTTATAATGATTTGTGTTGTATGAGTTTGTATTATATTG
CGTTGTGGACGTCTGTGCTAACCATGTGCTCAAACGAATTATTTTTACTATTATTTTATTGAGT
TTTCCTGTTTTGGCGGAACATGTTCAAACTTGCTTTTTGGTGGAATTTCACTTATGTGCATACG
AGTGGGTTACATTTTCCCAAAATAGTAGCACTTTTCGGAAGGTTGCTTATTATTGCTAATTTAG
CAATTGCATTCTTGTTTATCTTGATAGGTTTTGCGAGTGAGGGATTTTGGAGCTTGAATTTTAA
AAATATCAAAAACCATAGTTTGAATTTTAAAAAATCATCGGTTTTTACACATATGTTCATATAG
ATGCATTCCACAAGTGTATAATTTTTCACGATGAGATATGTTAACATGTGAGCTGCACAAAAAT
GAAAATTTTTGTGAATTTTTAATAGTGAACAATGCATGTACTGTTCACCATTATTAAAGCGTGA
ATTCTATTTTCTTTGTAGTTCACTAGAAGTGTGGCAAATTTTGGCAAGTTATTTCTATATGAGT
TCGACATTTCCATTACTCATCATGTTTAAGCATAATGCAGAAAAGAAGATGACAGCAGAAGTGT
TGTCAATAATGGAGAAATATAAGGAACCTAGCTCAAATATAATGAAAGCTGCCAATCTAACTTT
CTCCGGCGATGATGGCAAAACCAAGAGCACTAACAGGATGGGAATTAACTTGAAGGAGGCACTT
TCCCAACGCGATAAATGCATGGTAAGAAACCTTGTACTAAACGGATGCATTGAAATTTTAAATG
CAATCTATATCTTTGACACTTGTCAATGCAGGAGCTTGATGACATATGGTGTTATTGTGATTGG
ATGGCCCCTAGATACACAGTAGTACCTTCACTAGCATATGGTATGTGTAAGTACATTCGCTCTT
ATCTCTTTAATTTTCCAAATGGACAAGCATTGGGTTCATGGACCATGTAGGCACAATAATAAAG
AATTCCATACAAATGGGTCTTGGGCCACGTAATTCATGAGTTGCGTTTATTGAATGCAATTCCT
ATAGTGTCCCCTTAGTATCATGCACTGAAGTATTAGCATACATGGTGTACTATGCTTAGCATGC
TTGGGAAAAAGAATTTCCAAGTGATCAGATCTATAAACCACATGTCTATTCCCTGTTCAGGTGT
TAGGTTTATTCAACGTCACAGTTCGGTCCATGGTTACCAAAGACATGTGTTGCGCCATTGTACG
TTCTATAAGAGATCACCTCTACTCACTTCATTGCATTCCACTTGTCAAGAACTGAATAGGTCTC
CACCGAGCAATAGCGAGGAAGTGATGTTTGTTTTAGTGCCATCTTCTCTCATTCATAGTCATAG
AATGTTTTTCGAGCACTTATTCACTAGTCAATCCCCTATCTTTTCAGGAATGCACATCGACGAA
GTACGTTCGAAATGCGCTGATTTTCAGATGAGCATCACTGGCGATCCTCATCTGACCCCACATG
ATGCGAGGTGCTCTACAGCTGCCAATATGATATTGGAGCTTGGGAAGAAGGCGGAGGGAAAAGG
ACGAAATGATAACTGATGCAAAGTTGGTAGCTGGGAACTAGTAAGCGCTGGGGCCAATATTCGT
AAAGAGTGAGATAAAGCCACTTAGCTATTTGTTGTTCTAATGTATGAATGTTATAATGATTTGT
GTTGTATGAGTTTGTATTGTATTGCGTTGTGCACGTGCGTGCTAACCATGTGCTCAAATGAATT
ATTTTTACCATTATTTTATTGAGTTTTCCTTTTTGGGGGAACTTGTTCAAACTTGCTTTTGGGT
GGAATTTCACTTATGTGCATATGAGTGGGTTACATTTTCCCAAAATTCTAGCAATTTTTGGAAG
GTTGCTTATTATTGCTAATTTAGCAATTGCATTCCTGTTTATCTTGATAGGTTTTGCGAGTGAG
GCATTTTGGAGCTTGAATATTAAAAATATCAAAACCCATAGTTTGAATTTTGAAAAAATCATAG
GTTCTTATACATATGTTCATATGGATGCATTCCACATGTGTATAATGTTTCACGATGAAATATG
TTAACATGTGAGCTAGACAAAAATGAAAATTTTGTGAATTTTTAATAGTGAACAATGCATGTAC
AGTTCACCATTATCAAAGTTATGAATTTGTTTTTCTTTGTAGTTCACTAGAAGTGTGGCAAATT
TTGGCAAGTTATTTCGATATGAGTTCCACATTTCCATTACTCATCATGTTTAATTCTTATGAAG
AAGGGGACAGCAGAAGTGTTGTCAATAATGGAGAAGTATAAGGAACCTAGCTCAAATATAATGA
AAGCTGCCAATCTAACTTTCTCAGGCGACGATGGCAAAACCAAGAGCACTAACAGGATGGGATT
GAACTTGAAGGAGGCACTTTCCCAATGCCATAAATGCATGGTAAGAAACCTTGTGCTGAACAGA
TGCATTGAATTTTTAAATGCAATCTATATCTTTGACACTTATCAATGCTGGAGCTTGATGAGAT
ATGCTGTGATTGTGATTGGATGGCCCCTACATACATAGTAGTACATTCACTAGTAGATTGTATA
TGTAAGTACATTTACTCTTATCTCTTTAATTTTCCAAATGGACAAGCATTGGGTTCATGGACCA
TACAGGCACAATAATAAAGAATTCCATACAAATGGGTCTTCTGCCACATAATTCCAGACTAGCG
TTTATTGAATCGAATCTCTATAATGTCCCCTTAGTATCATGCATTGAACTGTTAGCTTACATGG
TGTACTATGGTTAGCATGCCCAGGAAAAAGAATTTCCTAGTGGTCAGATATATAAACTACTTTT
CTATGCCCTGTTCAGGTGTTAGGTTTATTCAACATCACGGTTCGGTCCATGGTTACCAAAGACA
TGCGTCGCGCCATTGTATGGTCTATAAGAGATCACCTCGACTAACTTCATTGCATTCCACTTGT
CAAGAACTGAGCAGGTCTCCACCTTGCAATAACCAGGAACTGTTTTTGGTTTTCATGGCATCTT
CTCACATTCATAGTCACATATTGTTTTTTGAGAACTTATTCATCAGTCAATATCTTATCTTTTG
AGGAATGCACGTCGGCGAAGTATGTTTGAAATGCCCTGAT

Figure S8. Sequence length variants of TaeSat33-54 satDNA family. **A.** TaeSat33-54 sequence as in Gálvez-Galván et al. (2024a). **B.** Sequence variant of 108 bp. **C.** As an example, we show the DNA sequence of two regions (928 bp and 956 bp, respectively) of chromosome 3D from which sequence length variants were extracted.

A.

```
>TaeSat33-54
TAAAATACAGTATTGCGATTATCCACCTCAATTTTATATCACTAATTTCAGTAC
```

B.

```
> Sequence variant of 108 bp
TAAATTCAGTATTGCGATTATTCACGTTAATATTTCAATACTGATTTCAATACTGAAAA
ATAGCAATAGACATCACCAGACTCAGTTGTATATCACTAATTTTGGTAC
```

C.

```
>Chr3D: 4306212- 4307139

TGAAATTCAGTATTGCGATTATTCGCCTTAATATTTCAATACTGATTTCAGTACTGAAA
TACAAAACGAGATCACCCGACTCACTTGTATATCACTAATTTTGGTACTAGAATTCAGT
ATTGTGATTCTTCACGTTAATTTTTCAATACTGATTTCAATACTAAAAATAGCACTAGA
ATGACCTGACTCAGTTTTATATCACCAATTTCGGTATAAAATTCAGTATTGCGATTATC
AAAGTTAATATGTGAGTATTGATTTGAGTACTAAAAAATAGCATGACTAACACCTCACT
CAGTTCTATATCTCTAATTTTGGTATTAAAACTCAGTATTATGATTATACATGTTAAAA
TTTCCGTACTGACTTCAATATTGAAATACAGCACCACGATGACCAGACTCGGTCGTGTA
TCACTAATTTCAGTACTAATATTTAGTATTGTGATTGTCCACATTAATATATCAATATT
GGTTTCAATACTGAAATATAAAATGACGATCATCACACTCAGTTGTATATCACTAATTT
TGGTATTAAGATTCAGTATTGCGATTATCCACTAATATTTCAATACTGATTTCAAGACT
TAAAAACAAAACGATGATCATGAGACTTAGCTGTATATCAAAAATTTGTTACTAATATT
CGGTATTGTGATTATCCATGTAAATATTGCAGTATTGATTTCAGTAGTAAAAAACAGCT
GCATGAACGCATGACTCAATTCTATATCACTAATTTAGTATTGCGATTATCCACTTTAA
TACTTTAATACTGAAATTCTATATCACCAATTCTATATCACTAATTTCGGTACTAAAAT
TCAGTGTTCTCATTATCCACGTTACTATTTCATTACTGATTTCAATACTGAAGTAGATC
ACCAAGATGACCCGACTATGTTCTATATCACCCATTTCGGTAC
```

>Chr3D: 4337987-4338942

GAAATTCAGTATTGCGATTATCCGCCTTAATATTTCAATACTGATTTCAGTACCGAAGT
ACAAAACGAGATCACCCGACTCACTTGTATATCACTAATTTTGGTACTAGAATTCAGTA
TTGTGATTCTTCACGTTAATTTTTCAATACTGATTTCAATACTAAAAATAACACTAGGA
TGACCTGACTCAGTTTTATATCACTAATTTCGATATAAAATTCAGTAGTGTGATTATCA
ATGTTAATATGTCAGTATTGATTTGAGTACTAAAAATAGCATGACTAACACCTCACTCA
GTTCTATATCACTAATTTTGGTATTAAAACTAAGTATTATTATTATGCATGTTAACATT
CCCGTACTGACTTCAATACTAAAATACAGCACCACGATGACCCGACTCGGTCGTGTATC
ACTAATTTCAGTACTAAAATTTAGTATTGTGATTATCCACATTAATATATCAATATTTG
TTTCAATACTGAAATATAAAATGACGATCATCACATTCAGTTGTATATCACTAATTTTG
GTATTAAAATTCAGTATTGCGATTATCCACTAATATTTCAATACTGATTTCAAGACTTA
AAAACAAAACGATGATCATGAGACTTAGTTGTATATCACTAATTTGTTACTAATATTCG
GTACTGCGATTATCCATGTAAATATTGCAGTGTTGATTTCCGTACTGAAAAACAGCTGG
ATGAACGCATGACTCAATTCTATATCACTAATTTTGGTACTAAAATGTAGCATTGAGAT
TAACCACGTTAATATTTCAATACTGAAAAAACAGCACCACGATGACCCGACTGAGTTGT
ATATTTCTAATTTCGGTACTAAAATTCAGTATTGTGATTATCCACCTTAATATTTCAAT
ATTGATTTAAGTACTGAAATATAGCACCAAGATGACCCGACTATGTTTTACATCACTGA
TTTTGGTACTAA

Figure S9. Sequence length variants of TaeSat34-175 satDNA family. **A-B.** Dot-plots of two regions of the *T. aestivum* CS genome.

A. Chr1A:69968735-69974249

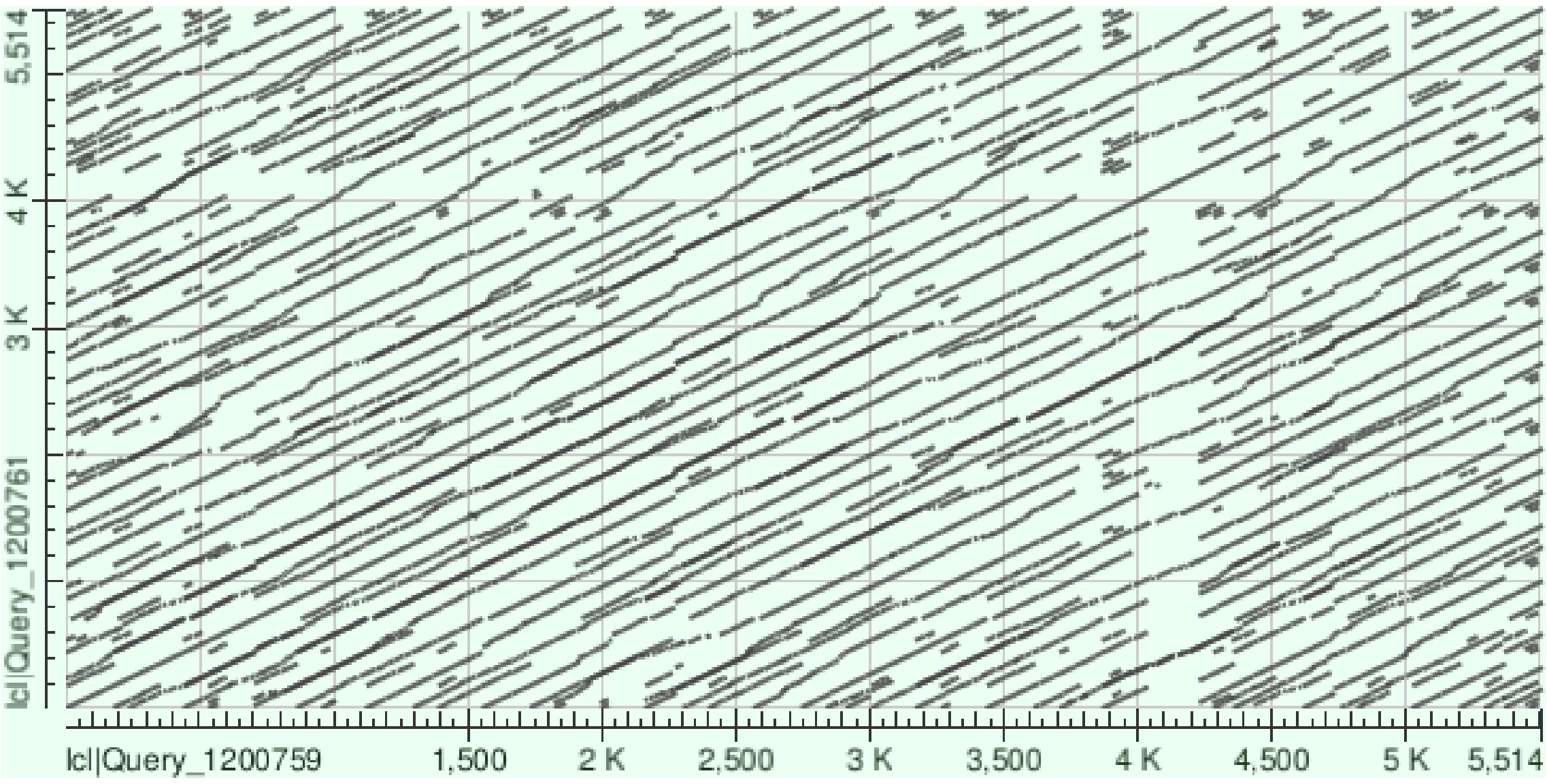

B. Chr4A:500636232-500644966

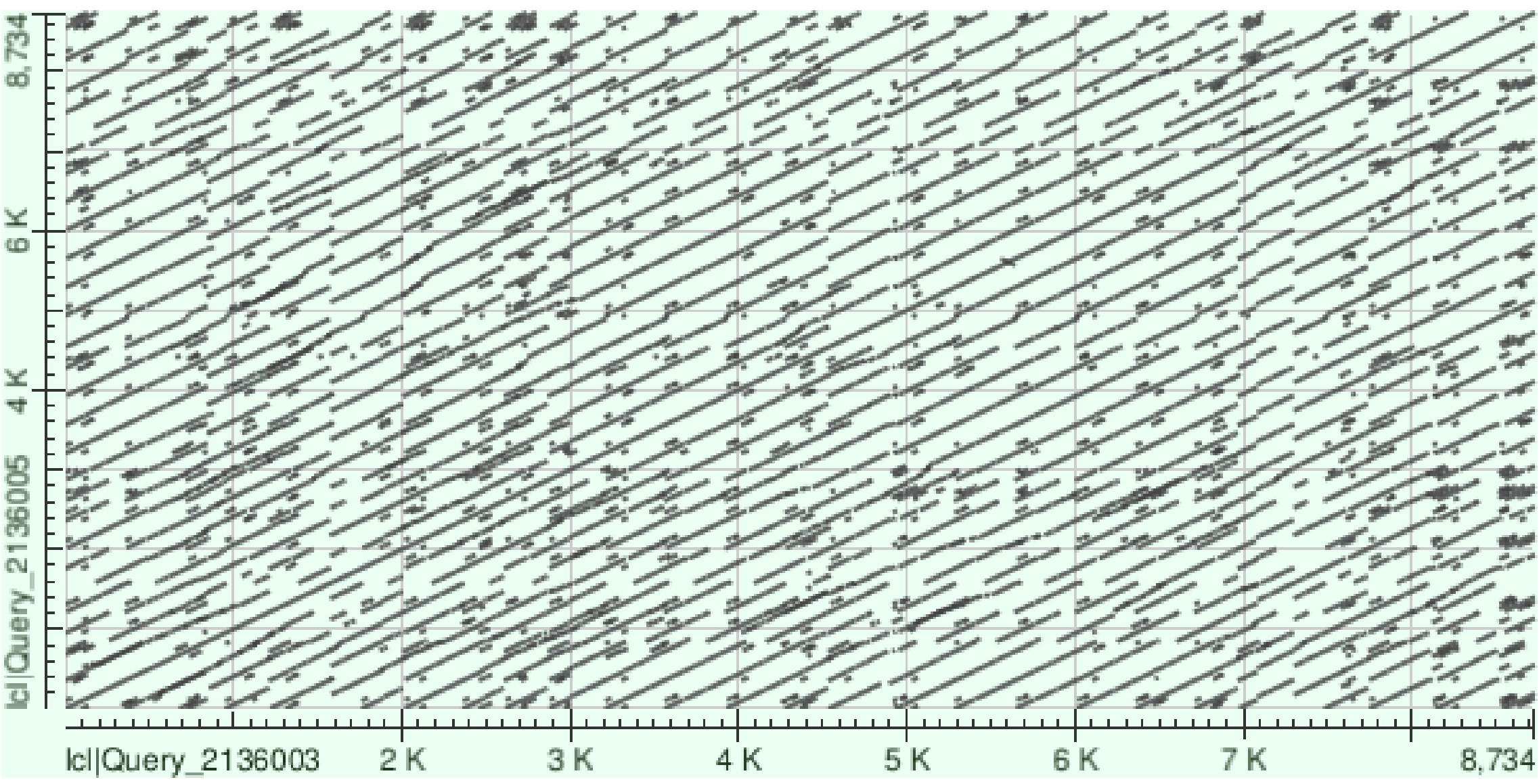

Figure S10. Long arm end (last 30 Mb) of each chromosome of *T. aestivum* cv. Chinese Spring showing the distribution of different satDNA families as obtained through the CHRISMAPP approach.

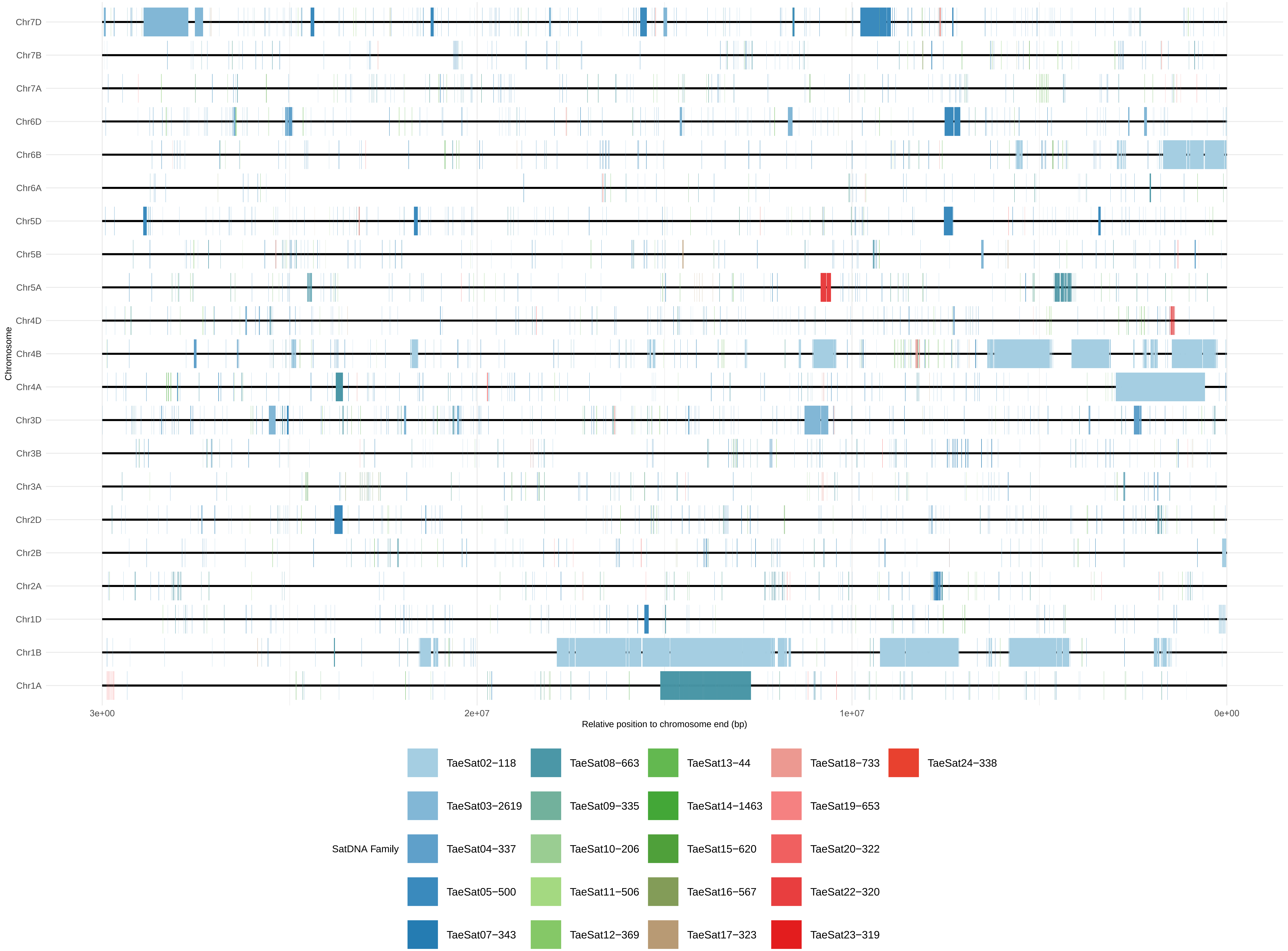

Chr7D
Chr7B
Chr7A
Chr6D
Chr6B
Chr6A
Chr5D
Chr5B
Chr5A
Chr4D
Chr4B
Chr4A
Chr3D
Chr3B
Chr3A
Chr2D
Chr2B
Chr2A
Chr1D
Chr1B
Chr1A
Chromosome
3e+00
2e+07
1e+07
0e+00
Relative position to chromosome end (bp)
SatDNA Family
TaeSat02−118
TaeSat03−2619
TaeSat04−337
TaeSat05−500
TaeSat07−343
TaeSat08−663
TaeSat09−335
TaeSat10−206
TaeSat11−506
TaeSat12−369
TaeSat13−44
TaeSat14−1463
TaeSat15−620
TaeSat16−567
TaeSat17−323
TaeSat18−733
TaeSat19−653
TaeSat20−322
TaeSat22−320
TaeSat23−319
TaeSat24−338

Figure S11. DNA sequence alignment between TtuSat12-178 sequence of *T. turgidum* (AABB) and the consensus of the homologous sequence found within the D subgenome of *T. aestivum* cv. CS.

```
Consensus_CS          AGTCGCGTTTGGTCGGTTTTAGGGCAAATTCTGTACAAGATGTCATATATGGGTACCTAA
TtuSat12-178          ACTCACATATGGCCGGTTTTAGGCCAAATTCACAACATGAGGTCATATATCGGTACCTAA
                      * ** * * *** ********** *******   *** ** ********* *********

Consensus_CS          CTTGGAAAAATTCTGTGAACGACATTTTTTAAGCATGGATTGGGTAGCAAAAAGTTATTT
TtuSat12-178          TTTGGCAAAATGTTGTGAACGGCGCTTGTCGAGGATGGTTTGGATAGCAAAAAGTTCTTC
                       **** *****  ******** *  ** *  ** **** **** ************ **

Consensus_CS          TGGACGAGCCACGAAAGAGGTAGTTATCGAGGAGTTTTTGCGTGTCCTTTGTTTT-CG
TtuSat12-178          TGGCCGTGCCATAGATGAGCAAGTTATCGAGGTGTTTTTGGCCGACCTTTGTTTTTCG
                      *** ** ****   * ***  *********** *******   * ********** **
```

Supplementary Information: Appendix 1

Detailed re-analysis of satellitome mapping facilitated by the telomere-to-telomere (T2T) assembly of bread wheat genome

Manuel A. Garrido-Ramos and Pilar Prieto

This Appendix includes the modifications and additions we have made to the CHRISMAPP_V2.1.R script (Rico-Porras et al. 2024). The development of the code has been assisted by Perplexity AI.

a) so that the script can recognize the .gff files generated by RepeatMasker:

```
# Extract the length information of each chromosome
lengths <- lapply(lines_length, function(line) {
  fields <- unlist(strsplit(line, " "))
  chromosome <- fields[2]
  length <- as.numeric(fields[4])
  data.frame(Chr = chromosome, Length = length)
})
```

b) so that the script identifies and draws the centromeres:

```
#Read the file that delimits centromeres in R
highlight_df <- read.table("Centromeres.txt", header = FALSE, col.names = c("Chr", "Start", "End"))
#Once all the data has been obtained, we can begin plotting.
graphic1 <- ggplot() +
  geom_segment(data=lengths, aes(x=0, xend=Length, y=Chr, yend=Chr), color="black", linewidth = 1) +
  geom_segment(data = ordered_data, aes(x = Start, y = Chr, xend = End, yend = Chr, color = Feature), linewidth = 15) +
  geom_rect(
    data = highlight_df,
    aes(
      xmin = Start,
      xmax = End,
      ymin = as.numeric(factor(Chr)) - 0.50,
      ymax = as.numeric(factor(Chr)) + 0.50
    ),
    fill = NA,
    color = "black",
    linewidth = 0.75
  ) +
theme_minimal() +
  labs(x="Position", y="Chromosome") + theme(axis.title = element_text(size = 10), axis.text = element_text(size = 10)) +
  scale_color_manual(name = "SatDNA Family", values = unique(ordered_data$Color), labels = unique(ordered_data$Feature)) +
  theme(legend.text = element_text(size = 12), legend.position = 'bottom')
```

“Centromeres.txt” is a file with 21 lines (one for each chromosome) and three columns: a) chromosome number and subgenome to which it belongs; b) centromere start site on each chromosome; and c) centromere end site on each chromosome, according to data provided by Liu et al. (2025).

c) We created a loop in the R script that generates chromosome graphs by windows of a specific length, 30 Mb in this example (about 5% of the average length of the species' chromosomes), so that we can analyze each chromosomal region in detail from one end to the other, window by window:

```
# Set window size
window_size <- 30000000
max_length <- max(lengths$Length)
windows <- seq(0, max_length, by = window_size)


# Plot all genome windows in PDF format by sections
pdf("all_the_windows.pdf", width = 20, height = 15)
for (i in seq_along(windows)[-length(windows)]) {
  window_start <- windows[i]
  window_end   <- windows[i + 1]
  ordered_data_window <- subset(ordered_data, Start < window_end & End > window_start)
  highlight_df_window <- subset(highlight_df, Start < window_end & End > window_start)
  ordered_data_window$Chr  <- factor(ordered_data_window$Chr,  levels = chr_levels)
  highlight_df_window$Chr  <- factor(highlight_df_window$Chr,  levels = chr_levels)

  # Adjust relative positions to the window
  ordered_data_window$Start <- pmax(ordered_data_window$Start, window_start)
  ordered_data_window$End   <- pmin(ordered_data_window$End, window_end)
  highlight_df_window$Start <- pmax(highlight_df_window$Start, window_start)
  highlight_df_window$End   <- pmin(highlight_df_window$End, window_end)
  lengths_window <- lengths
  lengths_window$Length <- pmax(lengths_window$Length, window_end)
  graphic1 <- ggplot() +
    geom_segment(data = lengths, aes(x = 0, xend = Length, y = Chr, yend = Chr), color = "black", linewidth = 1) +
    geom_segment(data = ordered_data_window, aes(x = Start, y = Chr, xend = End, yend = Chr, color = Feature), linewidth = 15) +
    theme_minimal() +
    geom_rect(
      data = highlight_df_window,
      aes(
        xmin = Start, xmax = End,
        ymin = as.numeric(factor(Chr)) - 0.50,
        ymax = as.numeric(factor(Chr)) + 0.50
      ),
      fill = NA, color = "black", linewidth = 0.75
    ) +
    coord_cartesian(xlim = c(window_start, window_end)) +
    labs(x = "Position", y = "Chromosome") +
    theme(axis.title = element_text(size = 10), axis.text = element_text(size = 10)) +
    scale_color_manual(
      name = "SatDNA Family",
      values = unique(ordered_data$Color),
      labels = unique(ordered_data$Feature)
    ) +
    theme(legend.text = element_text(size = 12), legend.position = 'bottom')
  print(graphic1)
}
dev.off()
```

d) since each chromosome of the species has a different length, to generate a graph that aligns the last 30 Mb of each chromosome (the end of the long arm of each chromosome), the following instructions have also been added:

```
# Calculate end window for each chromosome
final_windows <- lengths %>%
  mutate(WindowStart = pmax(1, Length - window_size + 1), WindowEnd = Length)

# Filter and adjust annotations to the final window
ends_data <- ordered_data %>%
  inner_join(final_windows, by = "Chr") %>%
  filter(End >= WindowStart) %>%
  mutate(Start_adj = pmax(Start, WindowStart) - WindowStart + 1,
         End_adj   = pmin(End, WindowEnd)     - WindowStart + 1,
         Chr = factor(Chr, levels = chr_levels))

# Plot extremes in a new PDF
pdf("ends_chromosomes.pdf", width = 20, height = 15)
ggplot() +
  geom_segment(
    data = lengths,
    aes(x = 1, xend = window_size, y = Chr, yend = Chr),
    color = "black", linewidth = 1
  ) +
  geom_segment(
    data = ends_data,
    aes(x = Start_adj, xend = End_adj, y = Chr, yend = Chr, color = Feature),
    linewidth = 15
  ) +
  geom_rect(
    data = ends_highlights,
    aes(
      xmin = Start_adj, xmax = End_adj,
      ymin = as.numeric(factor(Chr)) - 0.5,
      ymax = as.numeric(factor(Chr)) + 0.5
    ),
    fill = NA, color = "black", linewidth = 0.75
  ) +
  scale_x_continuous(
    limits = c(1, window_size),
    name = "Relative position to chromosome end (bp)"
  ) +
  labs(y = "Chromosome", color = "SatDNA Family") +
  scale_color_manual(
    name = "SatDNA Family",
    values = unique(ordered_data$Color),
    labels = unique(ordered_data$Feature)
  ) +
  theme_minimal() +
```

```
  theme(
    axis.title = element_text(size = 10),
    axis.text  = element_text(size = 10),
    legend.text = element_text(size = 12),
    legend.position = 'bottom'
  )
dev.off()
```

Supplementary Information: Appendix 2

Detailed re-analysis of satellitome mapping facilitated by the telomere-to-telomere (T2T) assembly of bread wheat genome

Manuel A. Garrido-Ramos and Pilar Prieto

This custom R script was used to organize the data and generate statistical summaries and comparative figures by patterns and satDNA families.

The development of the code has been assisted by Gemini AI.

```
# 1. LOAD REQUIRED LIBRARIES
# If you don't have them installed, install the libraries first
library(tidyverse)
library(readxl)
library(patchwork)

# 2. CONFIGURE PATH AND READ EXCEL FILE
# Replace "MySatelliteFile.xlsx" with the actual name of your Excel file
file_name <- "MySatelliteFile.xlsx"

# Read the names of all the sheets contained in the file
sheet_names <- excel_sheets(file_name)

# Read each sheet and combine them all into a single master data frame
complete_data <- sheet_names %>%
 map_df(function(sheet) {
  read_excel(file_name, sheet = sheet) %>%
   # Create the 'Family' column using the current sheet name
   mutate(Family = sheet)
 })

# 3. DATA PROCESSING AND PATTERN ASSIGNMENT
processed_data <- complete_data %>%
 mutate(
  # Calculate the exact number of repeat units (RU), forcing numeric data type
  RU = round(as.numeric(Array_length) / as.numeric(Monomer_length))
 ) %>%
 filter(!is.na(RU) & !is.na(Family)) %>%

 # Group by Family to analyze the characteristics of each satellite
 group_by(Family) %>%
 mutate(
  # Chained if_else to safeguard against Backtrace errors (LHS)
  Pattern = if_else(max(RU) >= 1000, "Pattern A",
       if_else(max(RU) < 10,    "Pattern C",
                  "Pattern B"))
 ) %>%
 ungroup()

# Save the consolidated master table as a backup CSV file
write.csv(processed_data, "Consolidated_Data_SatDNA.csv", row.names = FALSE)


# ====================================================================
# 4. PLOT GENERATION (BOX PLOTS)
# ====================================================================

# Define a fixed color palette for your structural patterns
colors_pattern <- c("Pattern A" = "#1F497D", "Pattern B" = "#31859C",
          "Pattern C" = "#E26B0A")
```

```
# -------------------------------------------------------------------------------------------
# FIGURE 1: GENERAL COMPARISON ACROSS THE 3 PROPOSED PATTERNS
# -------------------------------------------------------------------------------------------

p1 <- ggplot(processed_data, aes(x = Pattern, y = RU, fill = Pattern)) +
  geom_boxplot(outlier.alpha = 0.4, width = 0.6) +
  scale_y_log10() +
  scale_fill_manual(values = colors_pattern) +
  theme_bw() +
  labs(x = "Structural Pattern", y = "Number of repeat units per array (RU, log10
scale)") +
  theme(legend.position = "none", axis.text.x = element_text(angle = 45, hjust = 1))

p2 <- ggplot(processed_data, aes(x = Pattern, y = Monomer_length, fill = Pattern)) +
  geom_boxplot(outlier.alpha = 0.4, width = 0.6) +
  scale_fill_manual(values = colors_pattern) +
  theme_bw() +
  labs(x = "Structural Pattern", y = "Monomer length (bp)") +
  theme(legend.position = "none", axis.text.x = element_text(angle = 45, hjust = 1))

p3 <- ggplot(processed_data, aes(x = Pattern, y = Array_length, fill = Pattern)) +
  geom_boxplot(outlier.alpha = 0.4, width = 0.6) +
  scale_y_log10(labels = scales::comma) +
  scale_fill_manual(values = colors_pattern) +
  theme_bw() +
  labs(x = "Structural Pattern", y = "Array length (bp, log10 scale)") +
  theme(legend.position = "none", axis.text.x = element_text(angle = 45, hjust = 1))

pattern_figure <- p1 + p2 + p3 +
  plot_annotation(title = "Distribution of Global Parameters by satDNA Pattern",
          theme = theme(plot.title = element_text(face = "bold", size = 14, hjust =
0.5)))

ggsave("Figure1_Pattern_Comparison.png", plot = pattern_figure, width = 12,
height = 5, dpi = 300)

ggsave(filename = "Figure1_Pattern_Comparison.tiff", plot = pattern_figure, width =
12, height = 5, dpi = 300, compression = "lzw")


# -------------------------------------------------------------------------------------------
# FIGURE 2: DETAILED BREAKDOWN FOR EACH OF THE FAMILIES (WITH
DIVIDERS)
# -------------------------------------------------------------------------------------------

# 1. SET THE ORDER OF THE BLOCKS FROM LEFT TO RIGHT
actual_pattern_order <- c("Pattern A", "Pattern B", "Pattern C")

# 2. AUTOMATIC PRE-CALCULATION OF AXIS POSITIONS
ordered_counts <- processed_data %>%
  distinct(Family, Pattern) %>%
  mutate(Pattern = factor(Pattern, levels = actual_pattern_order)) %>%
  arrange(Pattern, Family) %>%
```

```
 count(Pattern)

# Safely extract the number of families for each actual block
n_A <- ordered_counts$n[ordered_counts$Pattern == "Pattern A"]
n_B <- ordered_counts$n[ordered_counts$Pattern == "Pattern B"]
n_C <- ordered_counts$n[ordered_counts$Pattern == "Pattern C"]

# Exact mathematical cutoff points on the X-axis for the A -> B -> C layout order
cutoff1 <- n_A + 0.5          # Exact divider line between A and B
cutoff2 <- n_A + n_B + 0.5     # Exact divider line between B and C

# Perfect midpoints on the X-axis to center the floating upper text labels
center_A <- (1 + cutoff1) / 2
center_B <- (cutoff1 + cutoff2) / 2
center_C <- (cutoff2 + (n_A + n_B + n_C)) / 2

# 3. PREPARE THE DATAFRAME FORCING THE FACTORED ORDER WITHOUT
USING REORDER IN GGPLOT
ordered_families <- processed_data %>%
 distinct(Family, Pattern) %>%
 mutate(Pattern = factor(Pattern, levels = actual_pattern_order)) %>%
 arrange(Pattern, Family) %>%
 pull(Family)

family_plot_data <- processed_data %>%
 mutate(Pattern = factor(Pattern, levels = actual_pattern_order),
     Family = factor(Family, levels = ordered_families)) # <--- ABSOLUTE X-AXIS
ORDER LOCKIN

# 4. SECURE PLOT GENERATION
families_figure <- ggplot(family_plot_data, aes(x = Family, y = RU, fill = Pattern)) +
 geom_boxplot(outlier.size = 1, outlier.alpha = 0.5) +
 scale_y_log10() +
 scale_fill_manual(values = colors_pattern) +
 theme_minimal() +

 # Vertical divider lines at the exact pre-calculated boundaries
 geom_vline(xintercept = c(cutoff1, cutoff2), linetype = "dashed", color = "grey40",
linewidth = 0.5) +

 # Top text labels raised to y = 12000 (floating cleanly above any potential outliers)
 annotate("text", x = center_A, y = 12000, label = "Pattern A", color =
colors_pattern["Pattern A"], fontface = "bold", size = 3.8) +
 annotate("text", x = center_B, y = 12000, label = "Pattern B", color =
colors_pattern["Pattern B"], fontface = "bold", size = 3.8) +
 annotate("text", x = center_C, y = 12000, label = "Pattern C", color =
colors_pattern["Pattern C"], fontface = "bold", size = 3.8) +

 labs(title = "Distribution of the Number of repeat units per array (RU) across the
satellite DNA families",
    subtitle = "Families grouped and color-coded according to their assigned
structural pattern",
    x = "satDNA families (Sorted by Pattern)",
```

```
    y = "Number of repeat units per array (RU, log10 scale)",
    fill = "Assigned Pattern") +
  theme(
    plot.title = element_text(face = "bold", size = 13),
    axis.text.x = element_text(angle = 90, vjust = 0.5, hjust = 1, size = 9),
    panel.grid.major.x = element_blank(),
    legend.position = "bottom"
  )

# Save final and corrected Figure 2 files
ggsave("Figure2_Families_Breakdown.png", plot = families_figure, width = 14,
height = 6, dpi = 300)
ggsave("Figure2_Families_Breakdown.tiff", plot = families_figure, width = 14, height
= 6, dpi = 300, compression = "lzw")
```
