# Supplementary material for: Detailed re-analysis of satellitome mapping facilitated by the telomere-to-telomere (T2T) assembly of bread wheat genome
Source: Plant Mol Biol. 2026 Aug 28;116(5):92. doi: 10.1007/s11103-026-01745-3 (PMC13525055; doi:10.1007/s11103-026-01745-3)
Supplement: Supplementary file 2 [file 11103_2026_1745_MOESM2_ESM.pdf]

Supplementary Figure 1

Detailed re-analysis of satellitome mapping facilitated by the telomere-to-telomere (T2T) assembly of bread wheat genome

Manuel A. Garrido-Ramos and Pilar Prieto

Figure S1. *T. aestivum* cv. Chinese Spring chromosomes representation showing the distribution of the different satDNA families individually represented (a page per satDNA) as obtained through the CHRISMAPP approach. The centromeres of each chromosome are indicated as boxes. Asterisks indicate loci detected in Gálvez-Galván et al. (2024a) using FISH (satellites 1, 5, 6, 11 and 21 gave a dispersed pattern while satellite 24 gave no FISH signals). The graphic only includes loci that comprise at least one full repeat unit.

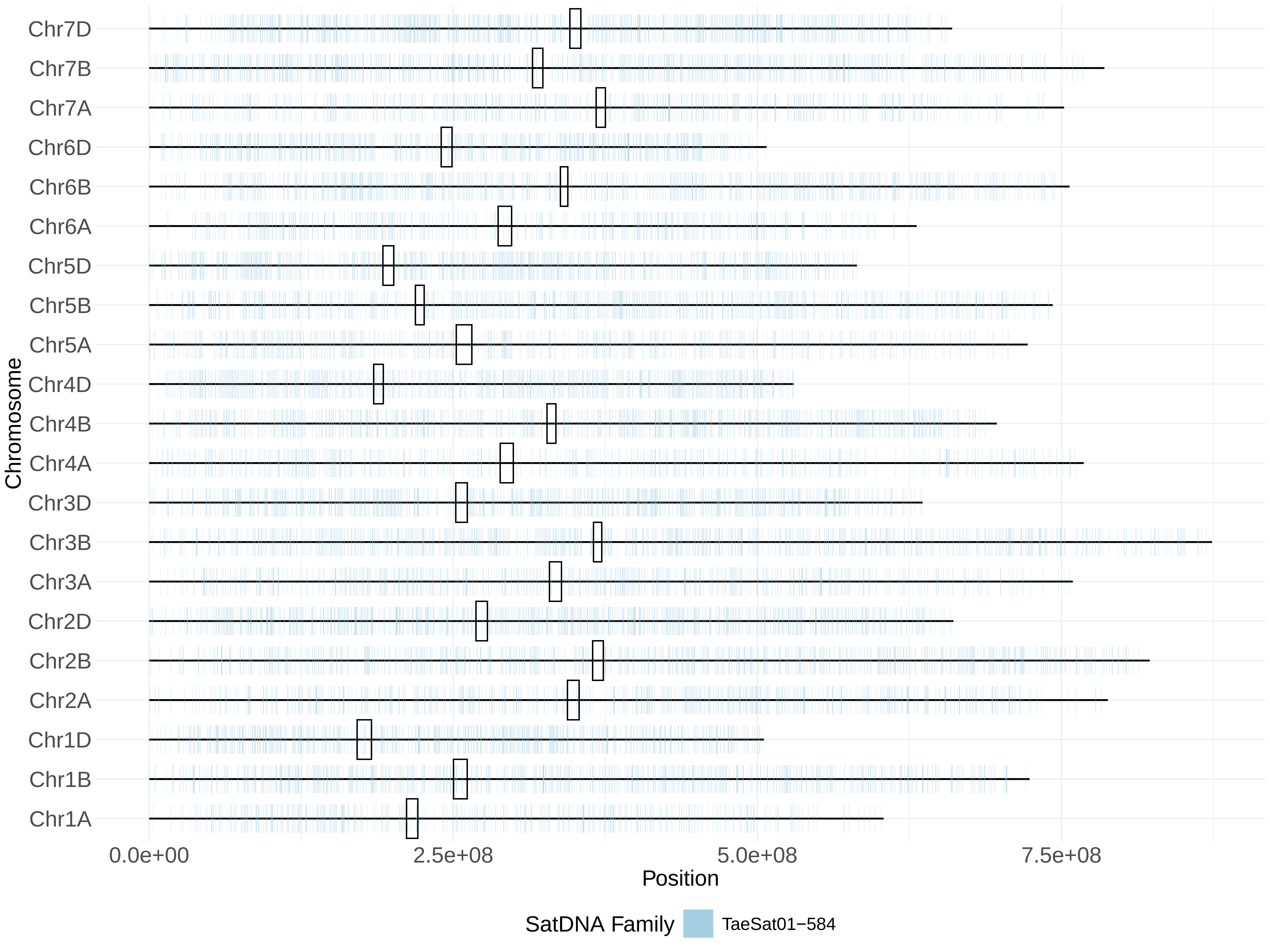

Chr7D
Chr7B
Chr7A
Chr6D
Chr6B
Chr6A
Chr5D
Chr5B
Chr5A
Chr4D
Chr4B
Chr4A
Chr3D
Chr3B
Chr3A
Chr2D
Chr2B
Chr2A
Chr1D
Chr1B
Chr1A
Chromosome
0.0e+00
2.5e+08
5.0e+08
7.5e+08
Position
SatDNA Family
TaeSat01−584

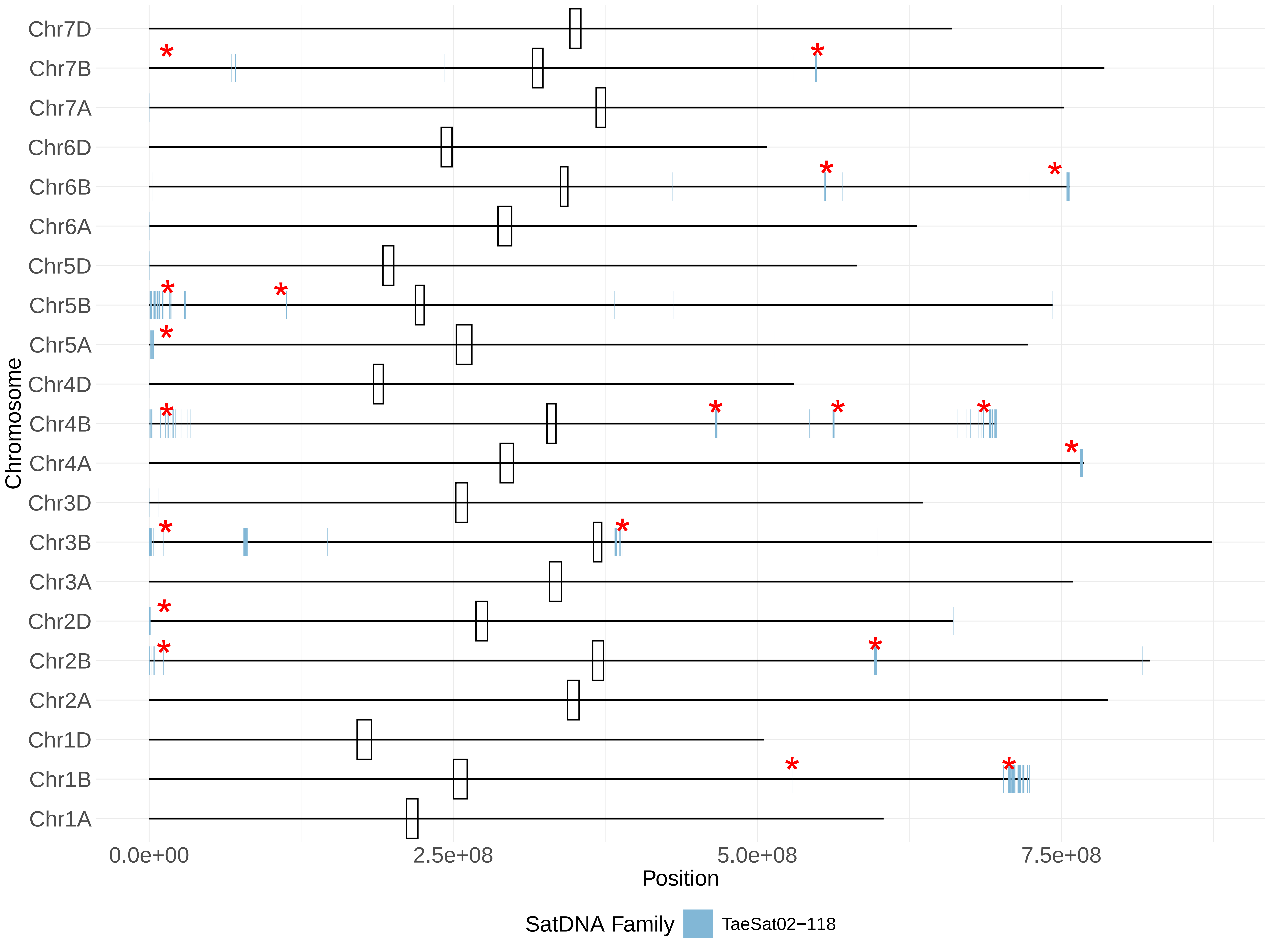
Chr7D
Chr7B
Chr7A
Chr6D
Chr6B
Chr6A
Chr5D
Chr5B
Chr5A
Chr4D
Chr4B
Chr4A
Chr3D
Chr3B
Chr3A
Chr2D
Chr2B
Chr2A
Chr1D
Chr1B
Chr1A
Chromosome
0.0e+00
2.5e+08
5.0e+08
7.5e+08
Position
SatDNA Family
TaeSat02−118

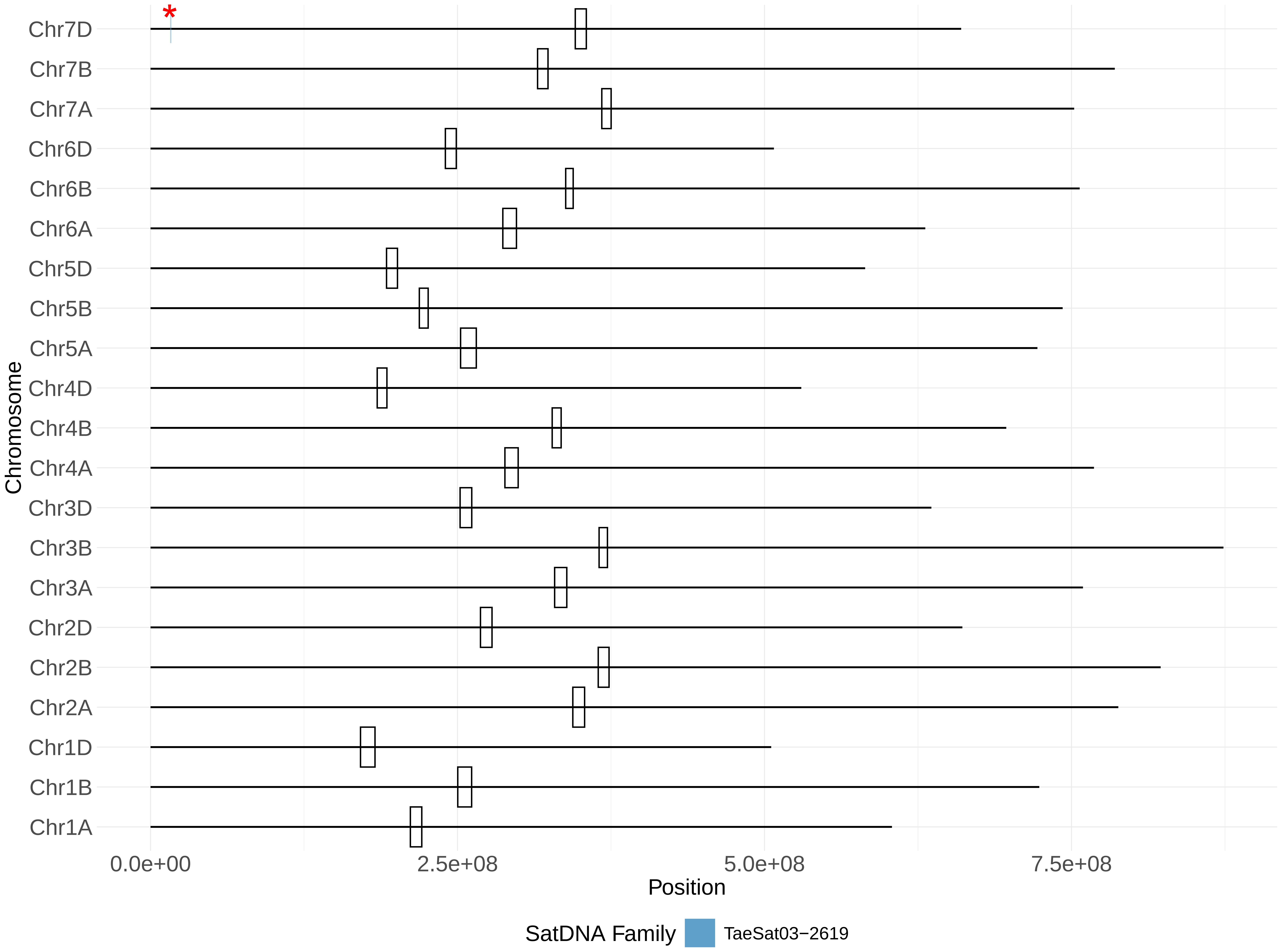
Chr7D
Chr7B
Chr7A
Chr6D
Chr6B
Chr6A
Chr5D
Chr5B
Chr5A
Chr4D
Chr4B
Chr4A
Chr3D
Chr3B
Chr3A
Chr2D
Chr2B
Chr2A
Chr1D
Chr1B
Chr1A
Chromosome
0.0e+00
2.5e+08
5.0e+08
7.5e+08
Position
SatDNA Family
TaeSat03−2619

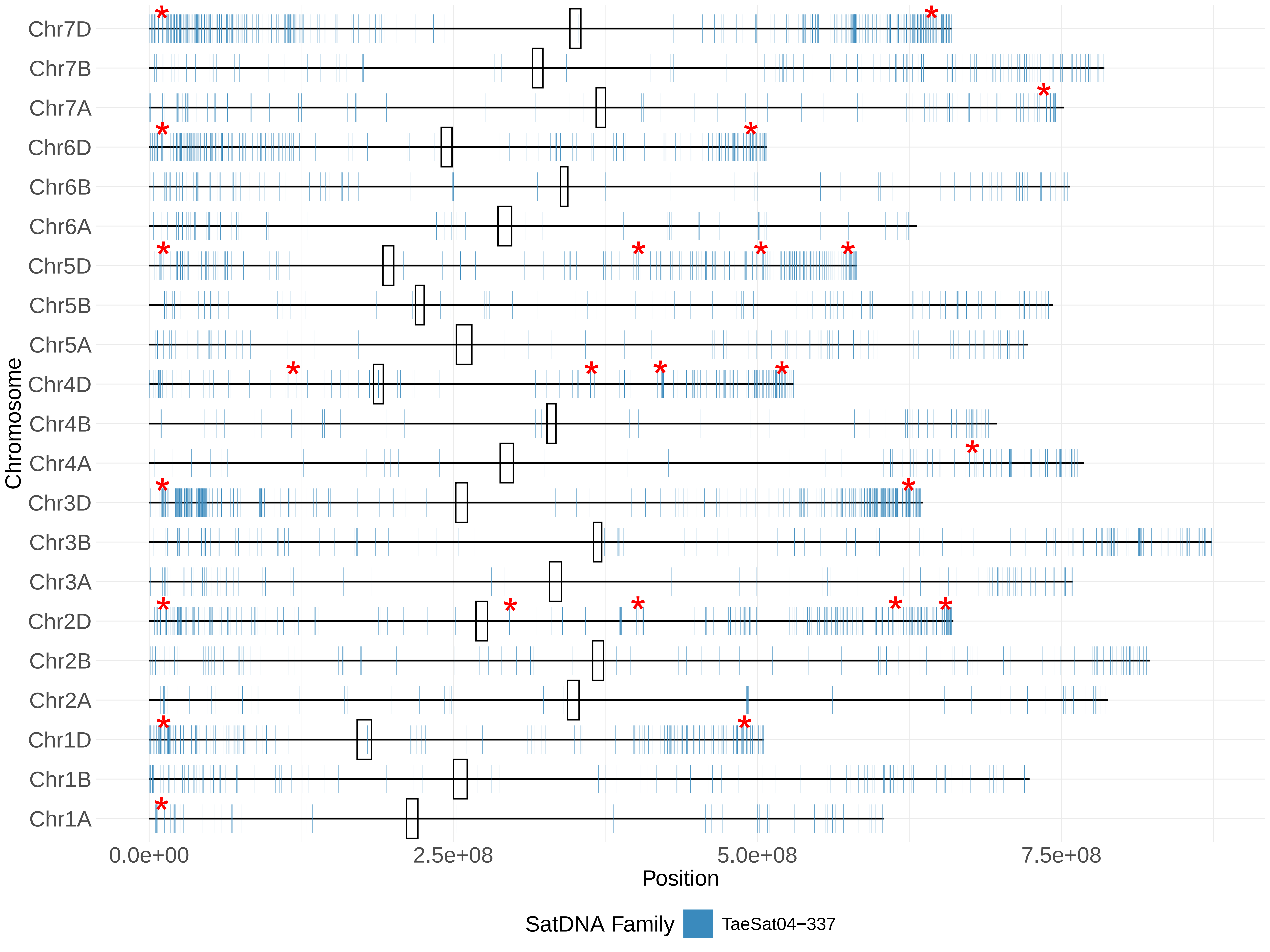

Chr7D
Chr7B
Chr7A
Chr6D
Chr6B
Chr6A
Chr5D
Chr5B
Chr5A
Chr4D
Chr4B
Chr4A
Chr3D
Chr3B
Chr3A
Chr2D
Chr2B
Chr2A
Chr1D
Chr1B
Chr1A
Chromosome
0.0e+00
2.5e+08
5.0e+08
7.5e+08
Position
SatDNA Family
TaeSat04−337

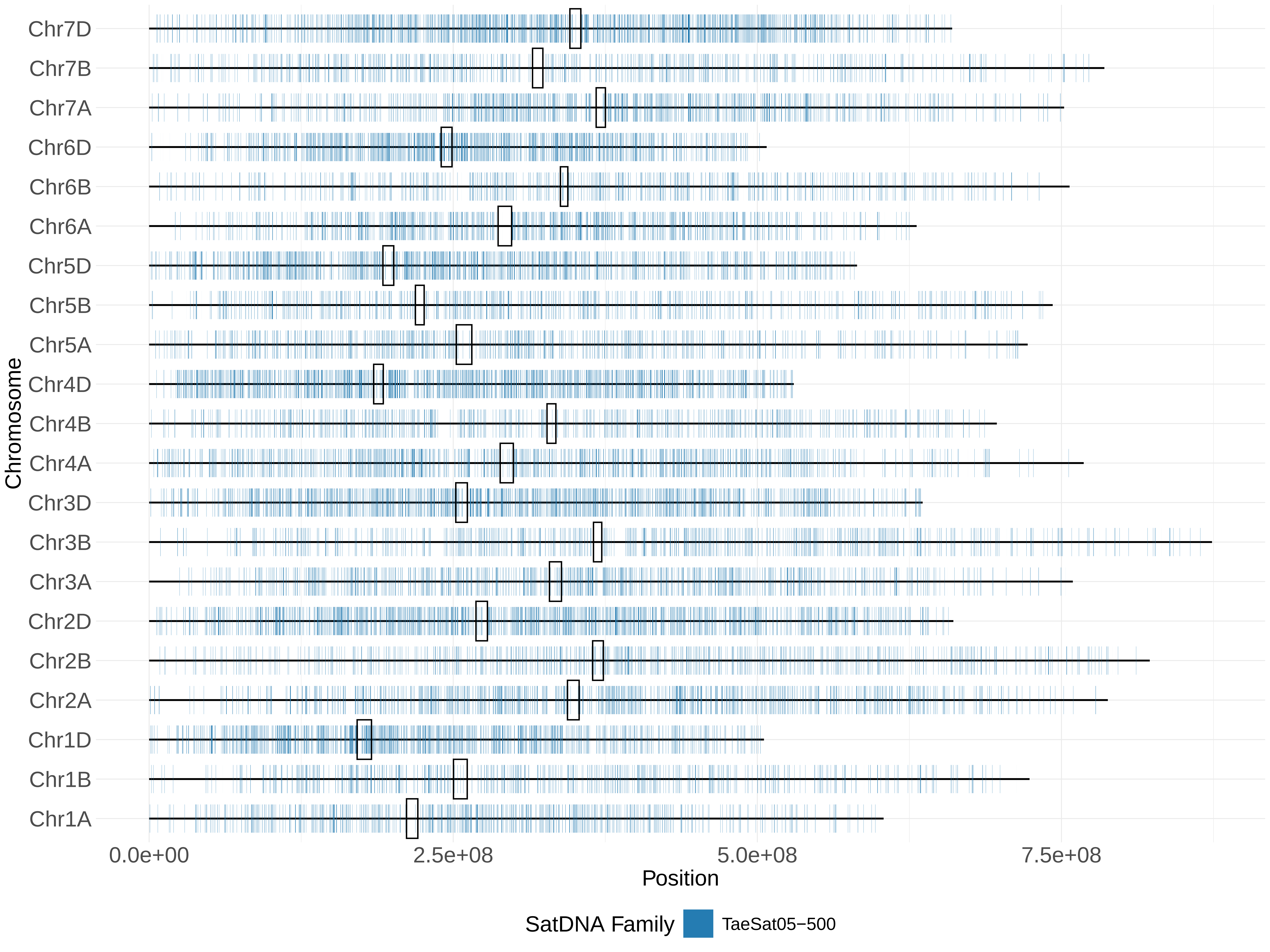
Chr7D
Chr7B
Chr7A
Chr6D
Chr6B
Chr6A
Chr5D
Chr5B
Chr5A
Chr4D
Chr4B
Chr4A
Chr3D
Chr3B
Chr3A
Chr2D
Chr2B
Chr2A
Chr1D
Chr1B
Chr1A
Chromosome
0.0e+00
2.5e+08
5.0e+08
7.5e+08
Position
SatDNA Family
TaeSat05−500

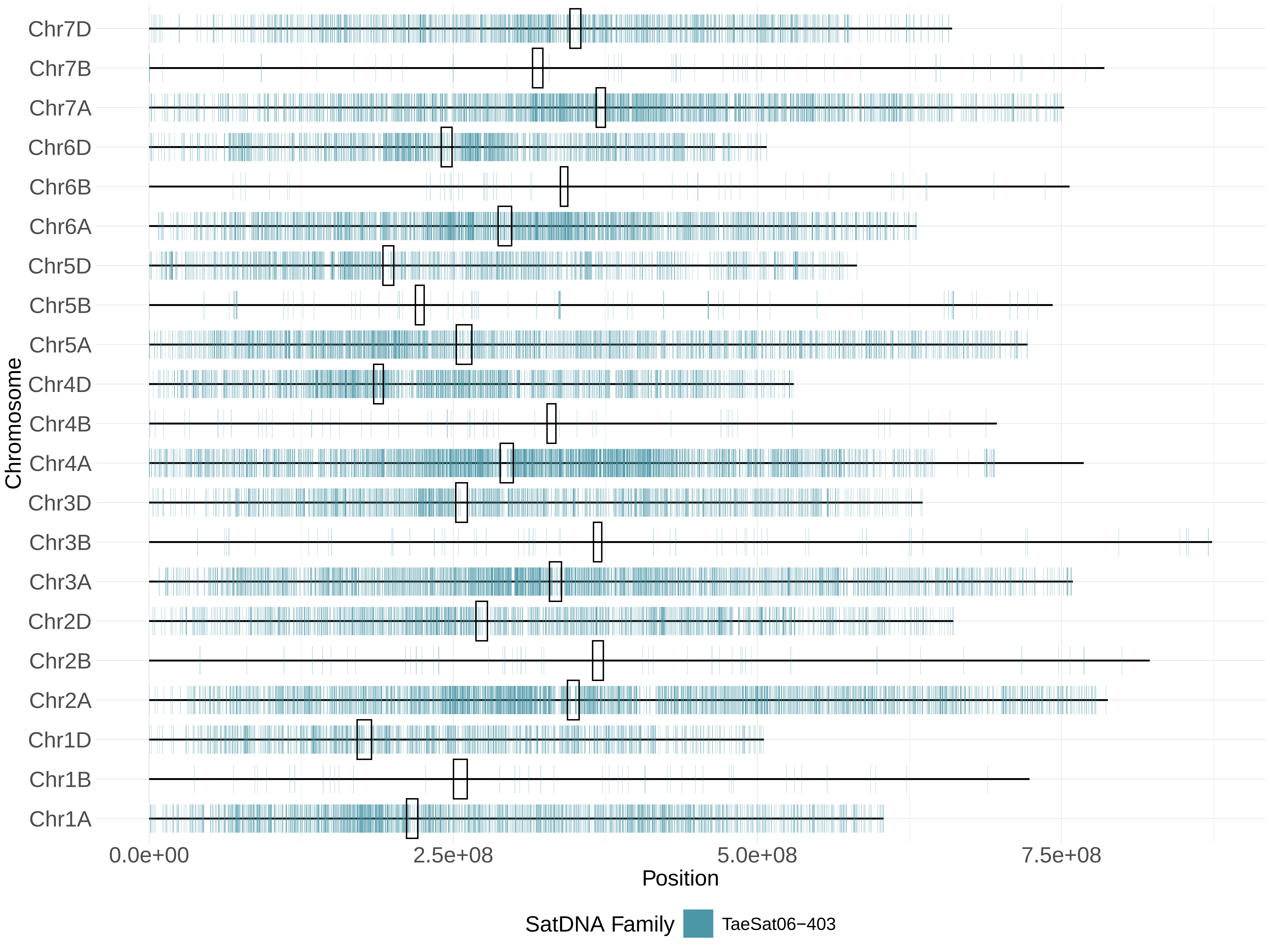

Chr7D
Chr7B
Chr7A
Chr6D
Chr6B
Chr6A
Chr5D
Chr5B
Chr5A
Chr4D
Chr4B
Chr4A
Chr3D
Chr3B
Chr3A
Chr2D
Chr2B
Chr2A
Chr1D
Chr1B
Chr1A
Chromosome
0.0e+00
2.5e+08
5.0e+08
7.5e+08
Position
SatDNA Family
TaeSat06−403

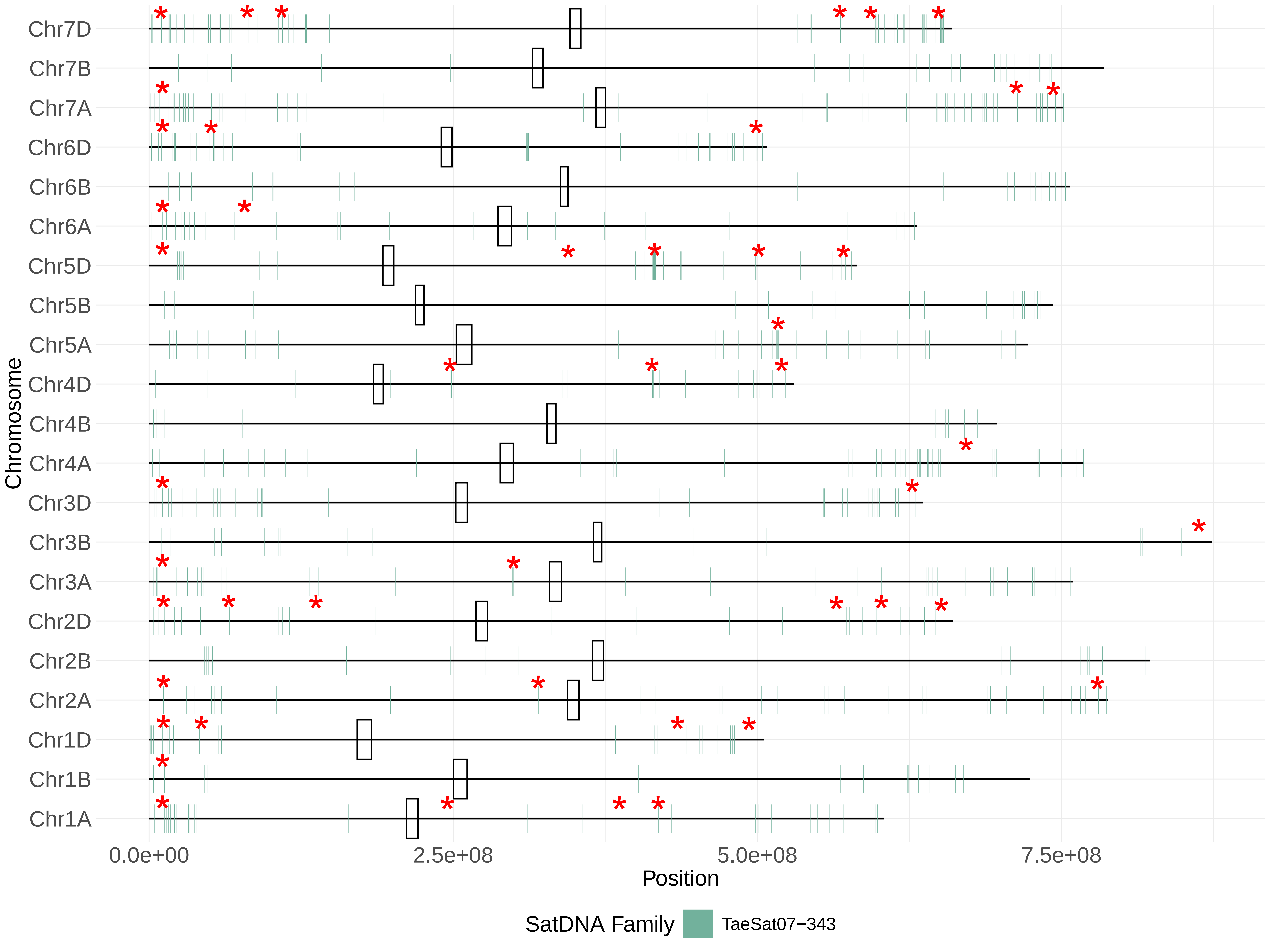

Chr7D
Chr7B
Chr7A
Chr6D
Chr6B
Chr6A
Chr5D
Chr5B
Chr5A
Chr4D
Chr4B
Chr4A
Chr3D
Chr3B
Chr3A
Chr2D
Chr2B
Chr2A
Chr1D
Chr1B
Chr1A
Chromosome
0.0e+00
2.5e+08
5.0e+08
7.5e+08
Position
SatDNA Family
TaeSat07−343

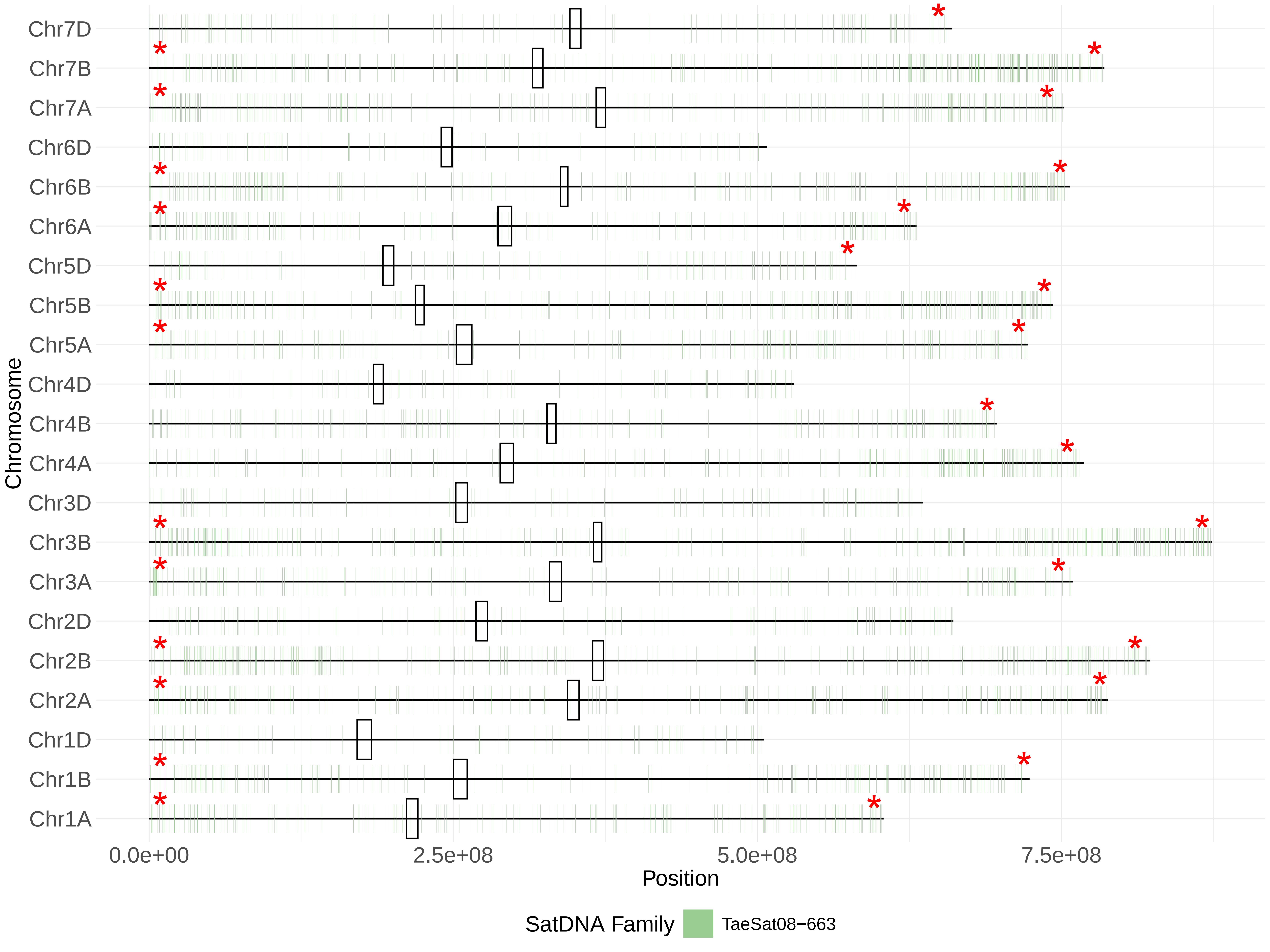

Chr7D
Chr7B
Chr7A
Chr6D
Chr6B
Chr6A
Chr5D
Chr5B
Chr5A
Chr4D
Chr4B
Chr4A
Chr3D
Chr3B
Chr3A
Chr2D
Chr2B
Chr2A
Chr1D
Chr1B
Chr1A
Chromosome
0.0e+00
2.5e+08
5.0e+08
7.5e+08
Position
SatDNA Family
TaeSat08−663

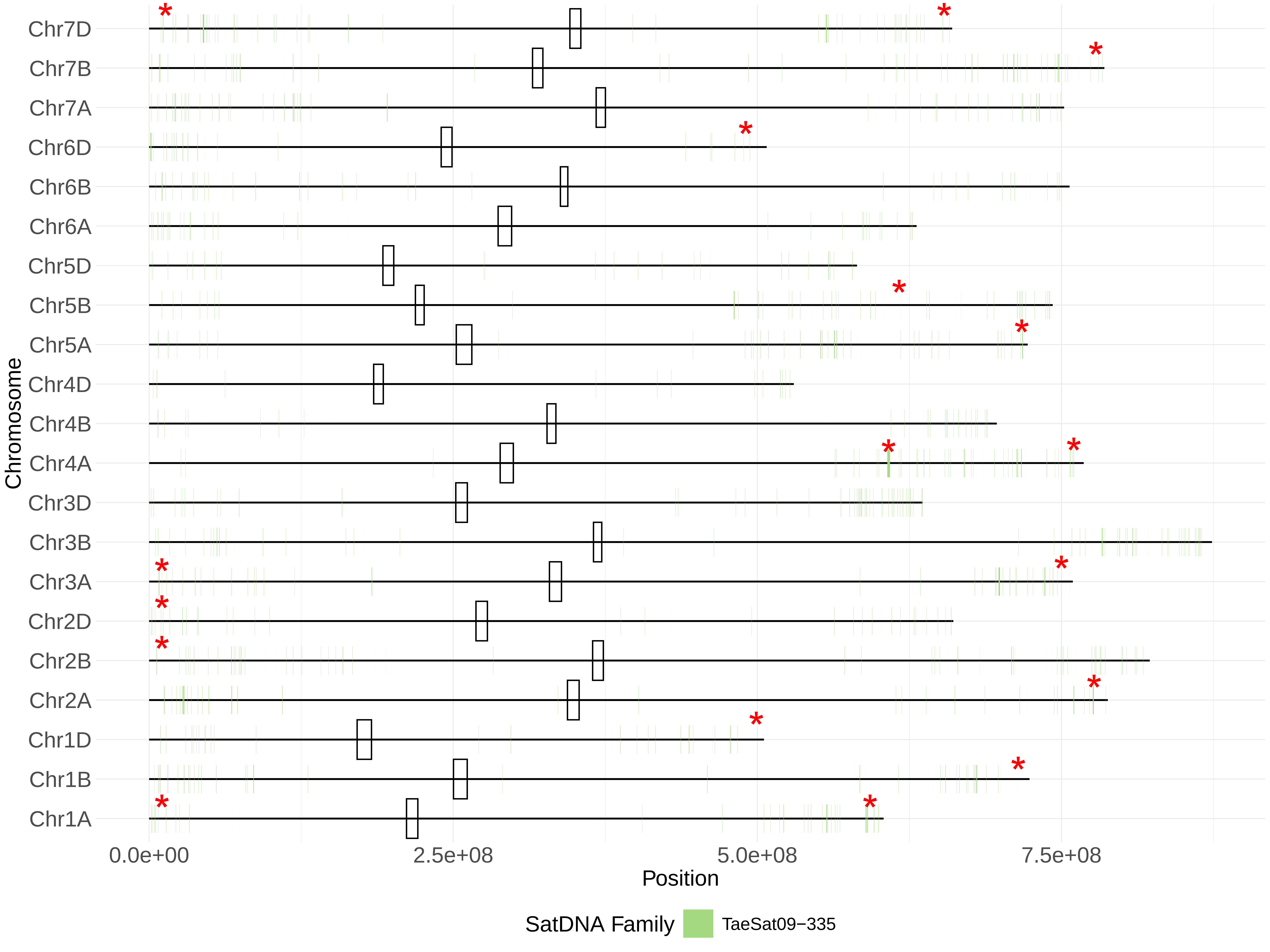

Chr7D
Chr7B
Chr7A
Chr6D
Chr6B
Chr6A
Chr5D
Chr5B
Chr5A
Chr4D
Chr4B
Chr4A
Chr3D
Chr3B
Chr3A
Chr2D
Chr2B
Chr2A
Chr1D
Chr1B
Chr1A
Chromosome
0.0e+00
2.5e+08
5.0e+08
7.5e+08
Position
SatDNA Family
TaeSat09−335

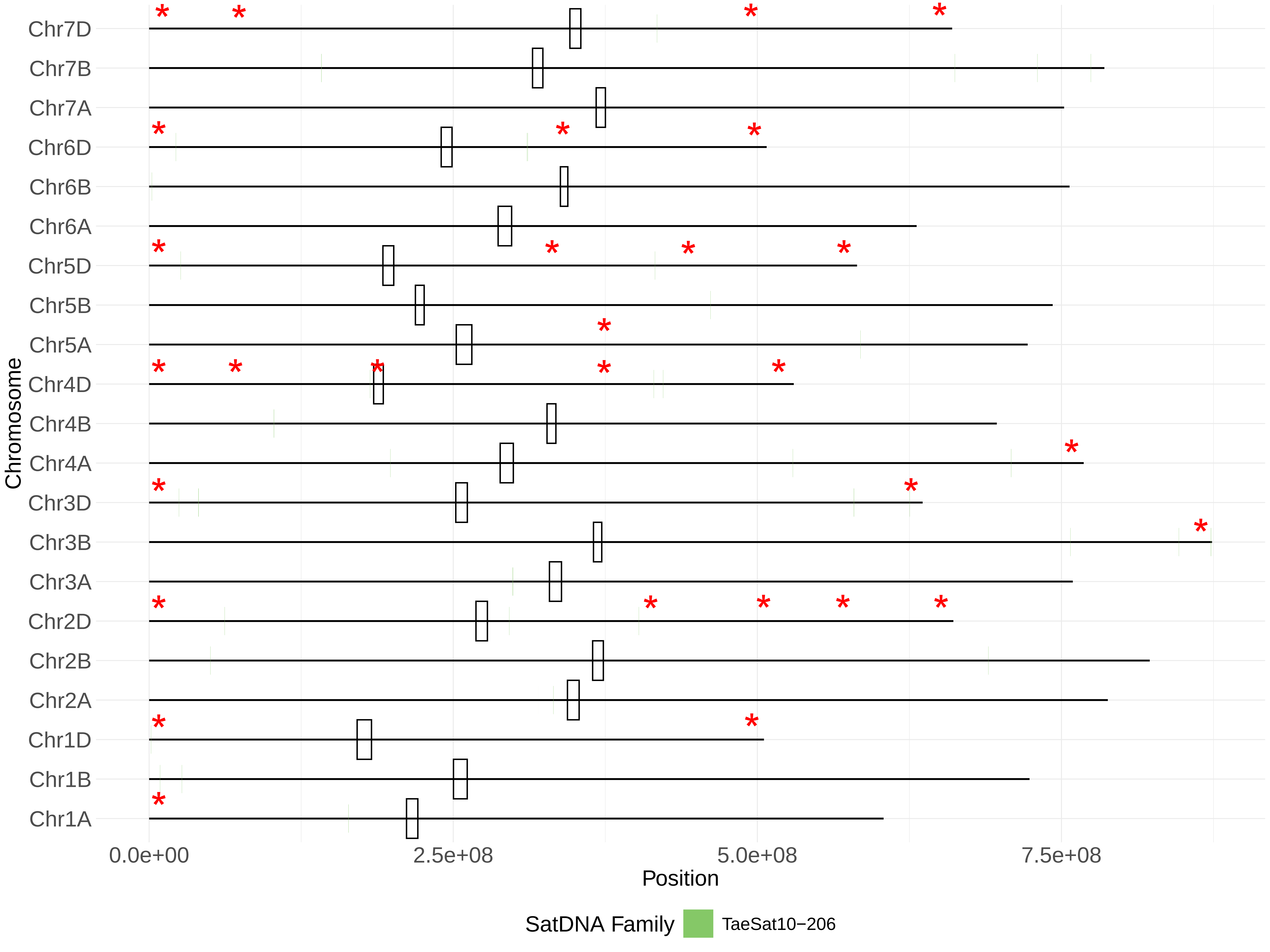
Chr7D
Chr7B
Chr7A
Chr6D
Chr6B
Chr6A
Chr5D
Chr5B
Chr5A
Chr4D
Chr4B
Chr4A
Chr3D
Chr3B
Chr3A
Chr2D
Chr2B
Chr2A
Chr1D
Chr1B
Chr1A
Chromosome
0.0e+00
2.5e+08
5.0e+08
7.5e+08
Position
SatDNA Family
TaeSat10−206

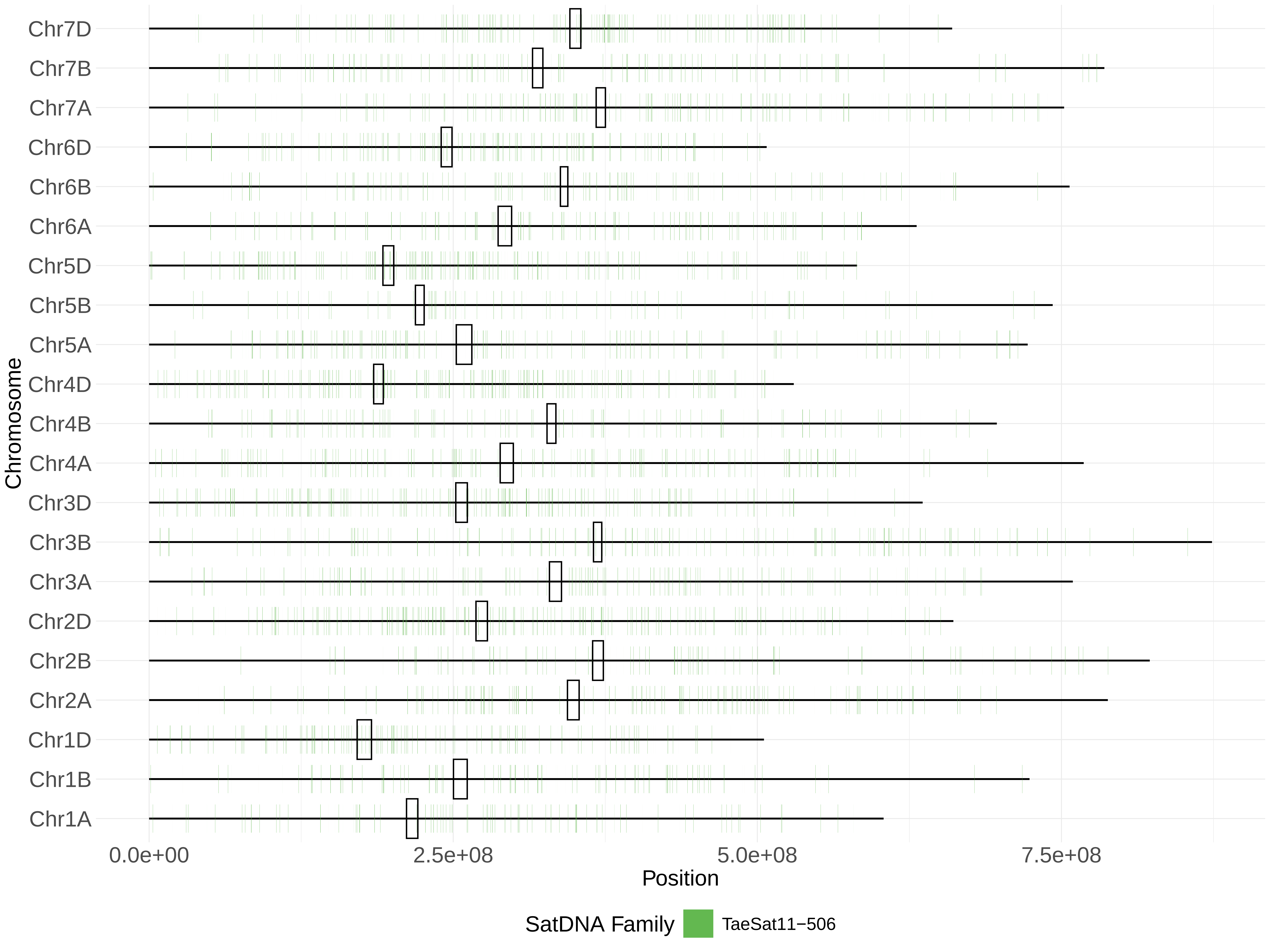
Chr7D
Chr7B
Chr7A
Chr6D
Chr6B
Chr6A
Chr5D
Chr5B
Chr5A
Chr4D
Chr4B
Chr4A
Chr3D
Chr3B
Chr3A
Chr2D
Chr2B
Chr2A
Chr1D
Chr1B
Chr1A
Chromosome
0.0e+00
2.5e+08
5.0e+08
7.5e+08
Position
SatDNA Family
TaeSat11−506

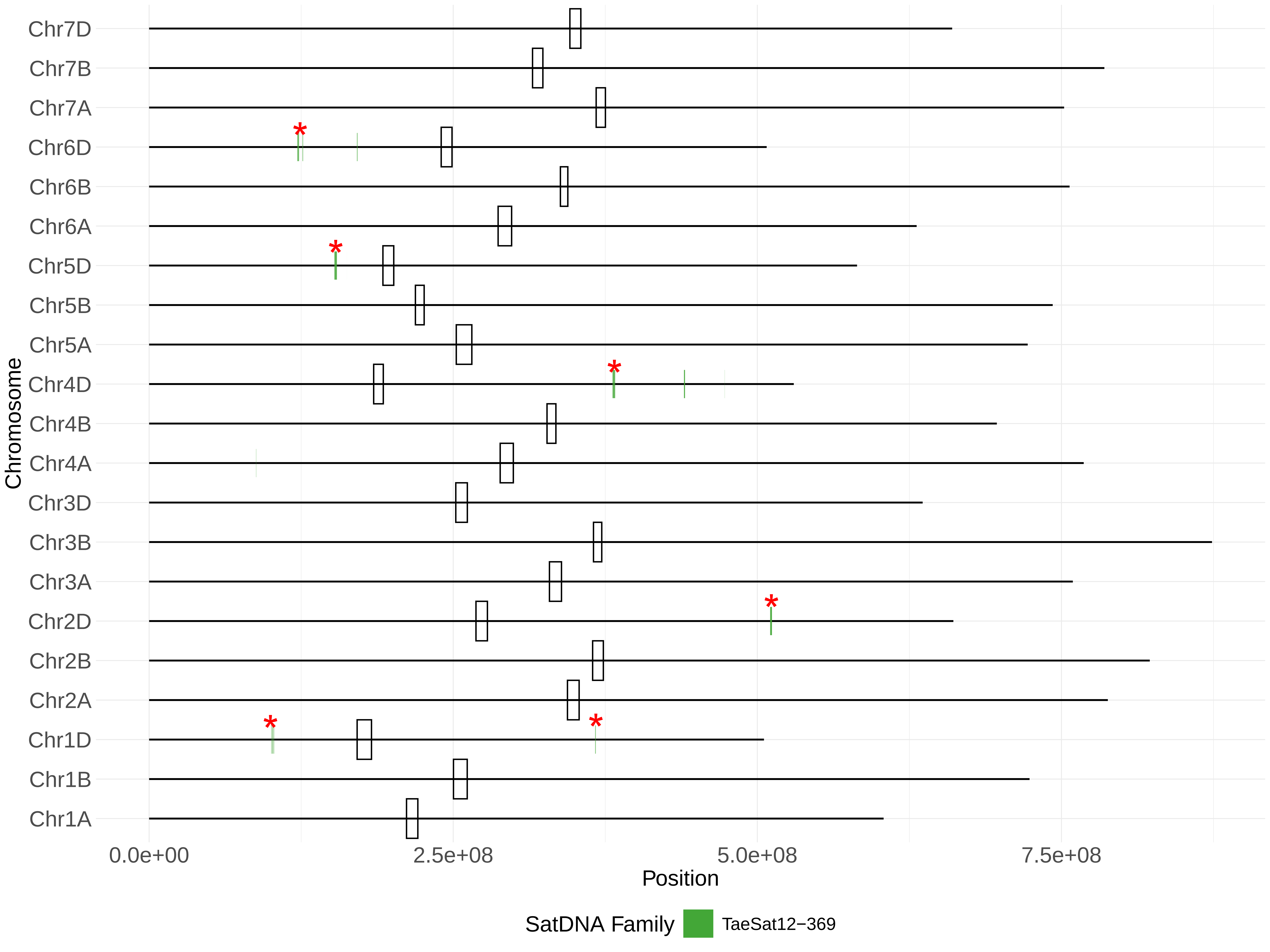
Chr7D
Chr7B
Chr7A
Chr6D
Chr6B
Chr6A
Chr5D
Chr5B
Chr5A
Chr4D
Chr4B
Chr4A
Chr3D
Chr3B
Chr3A
Chr2D
Chr2B
Chr2A
Chr1D
Chr1B
Chr1A
Chromosome
0.0e+00
2.5e+08
5.0e+08
7.5e+08
Position
SatDNA Family
TaeSat12−369

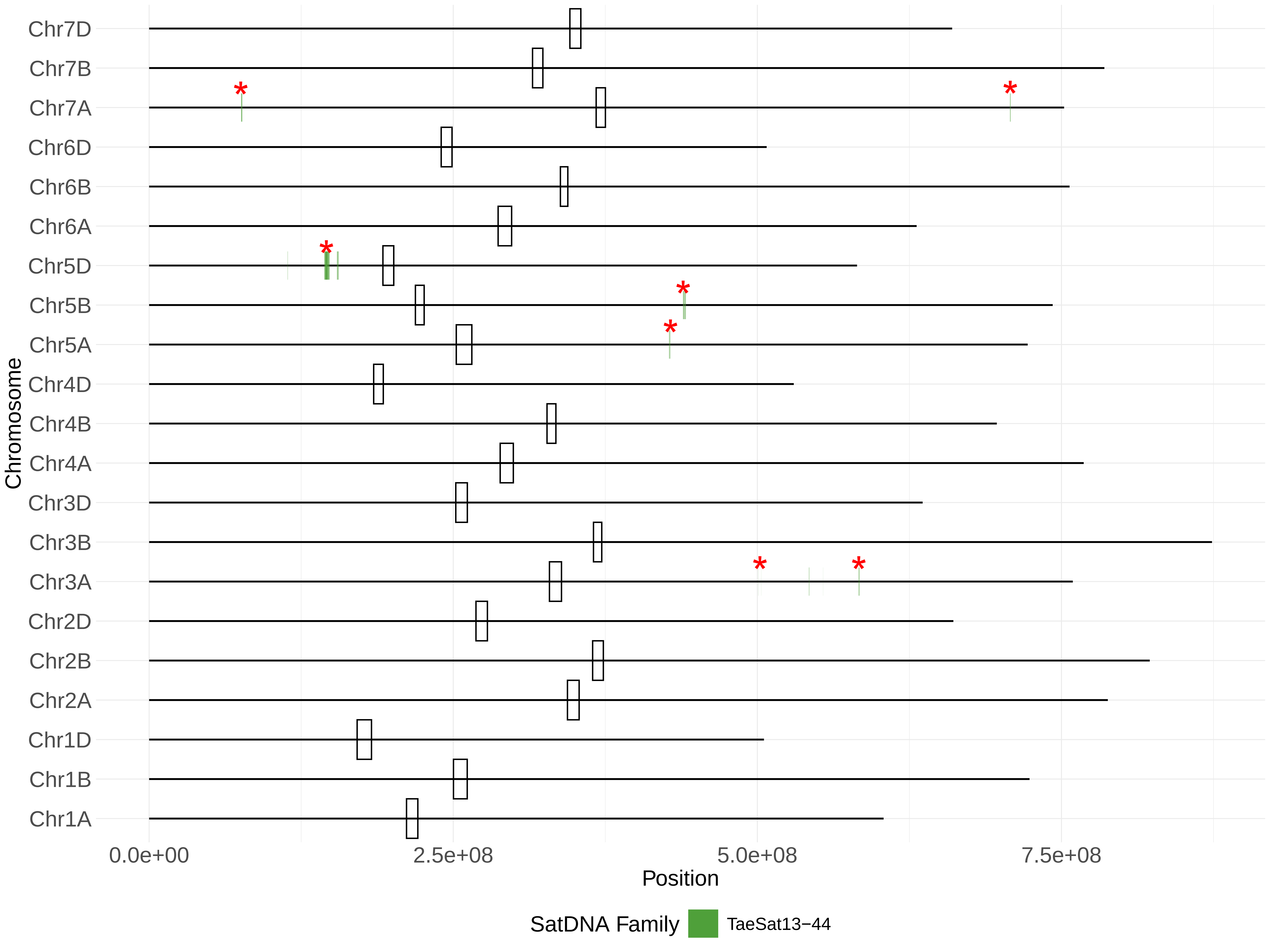
Chr7D
Chr7B
Chr7A
Chr6D
Chr6B
Chr6A
Chr5D
Chr5B
Chr5A
Chr4D
Chr4B
Chr4A
Chr3D
Chr3B
Chr3A
Chr2D
Chr2B
Chr2A
Chr1D
Chr1B
Chr1A
Chromosome
0.0e+00
2.5e+08
5.0e+08
7.5e+08
Position
SatDNA Family
TaeSat13−44

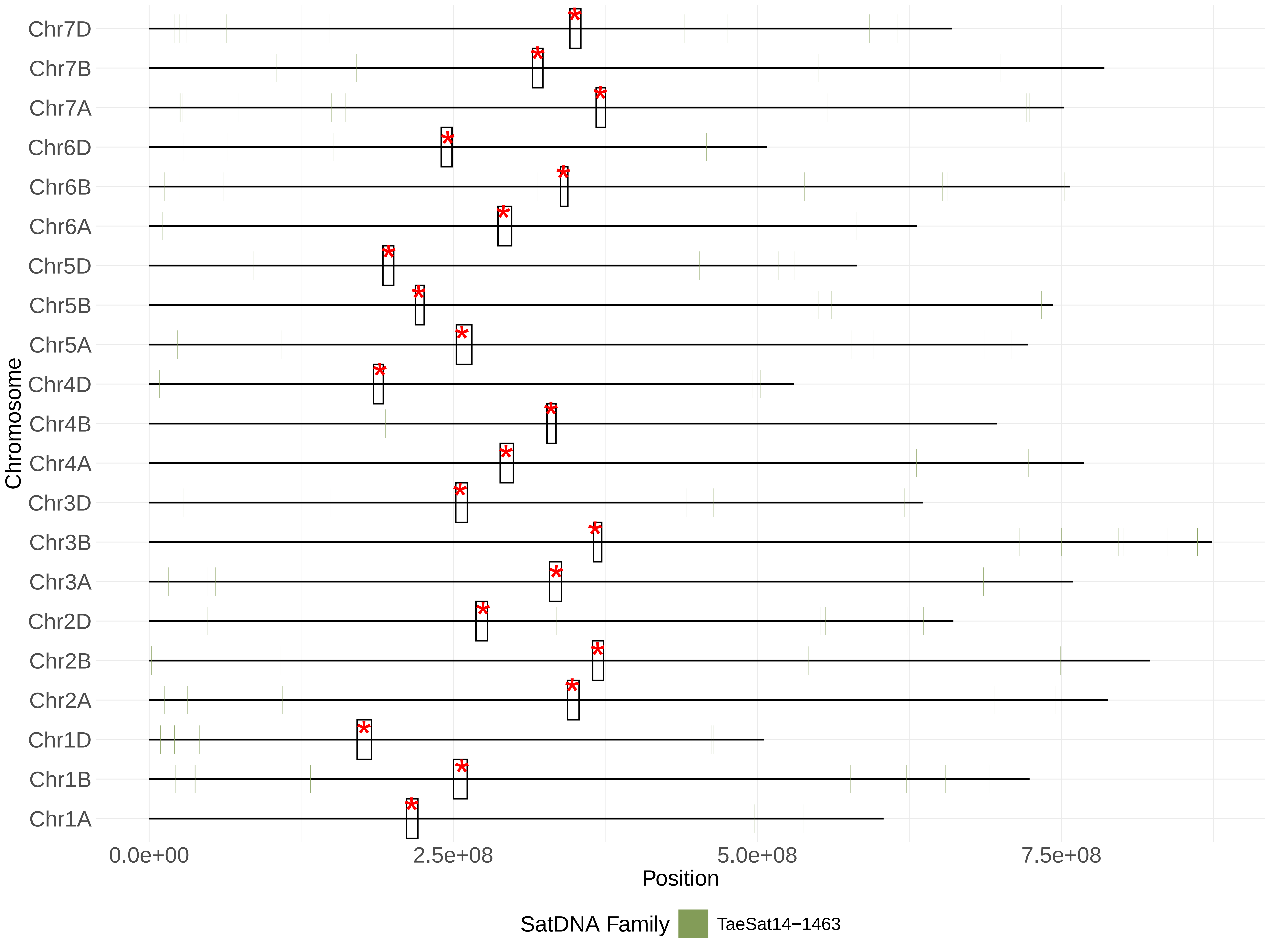
Chr7D
Chr7B
Chr7A
Chr6D
Chr6B
Chr6A
Chr5D
Chr5B
Chr5A
Chr4D
Chr4B
Chr4A
Chr3D
Chr3B
Chr3A
Chr2D
Chr2B
Chr2A
Chr1D
Chr1B
Chr1A
Chromosome
0.0e+00
2.5e+08
5.0e+08
7.5e+08
Position
SatDNA Family
TaeSat14−1463

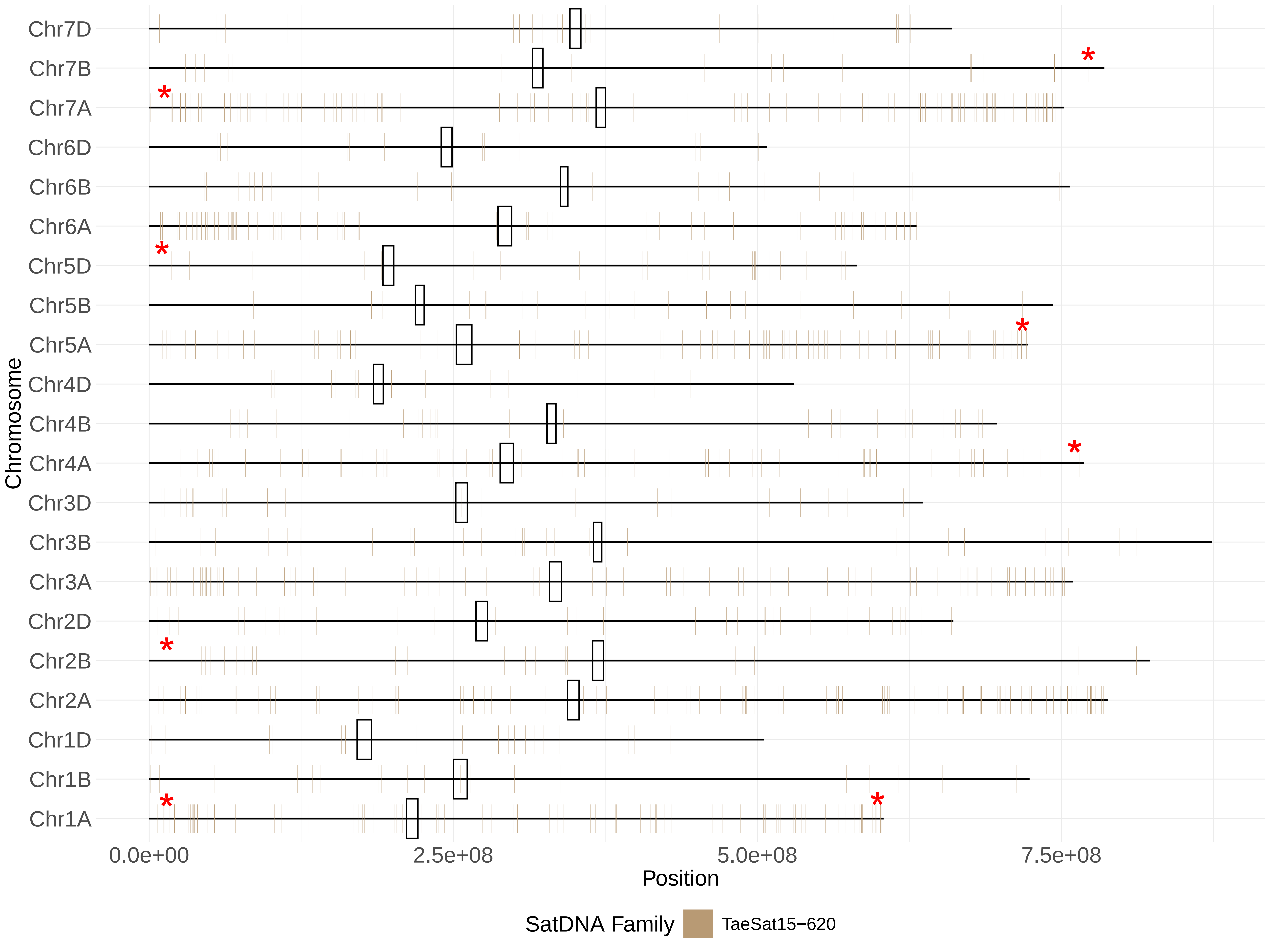

Chr7D
Chr7B
Chr7A
Chr6D
Chr6B
Chr6A
Chr5D
Chr5B
Chr5A
Chr4D
Chr4B
Chr4A
Chr3D
Chr3B
Chr3A
Chr2D
Chr2B
Chr2A
Chr1D
Chr1B
Chr1A
Chromosome
0.0e+00
2.5e+08
5.0e+08
7.5e+08
Position
SatDNA Family
TaeSat15−620

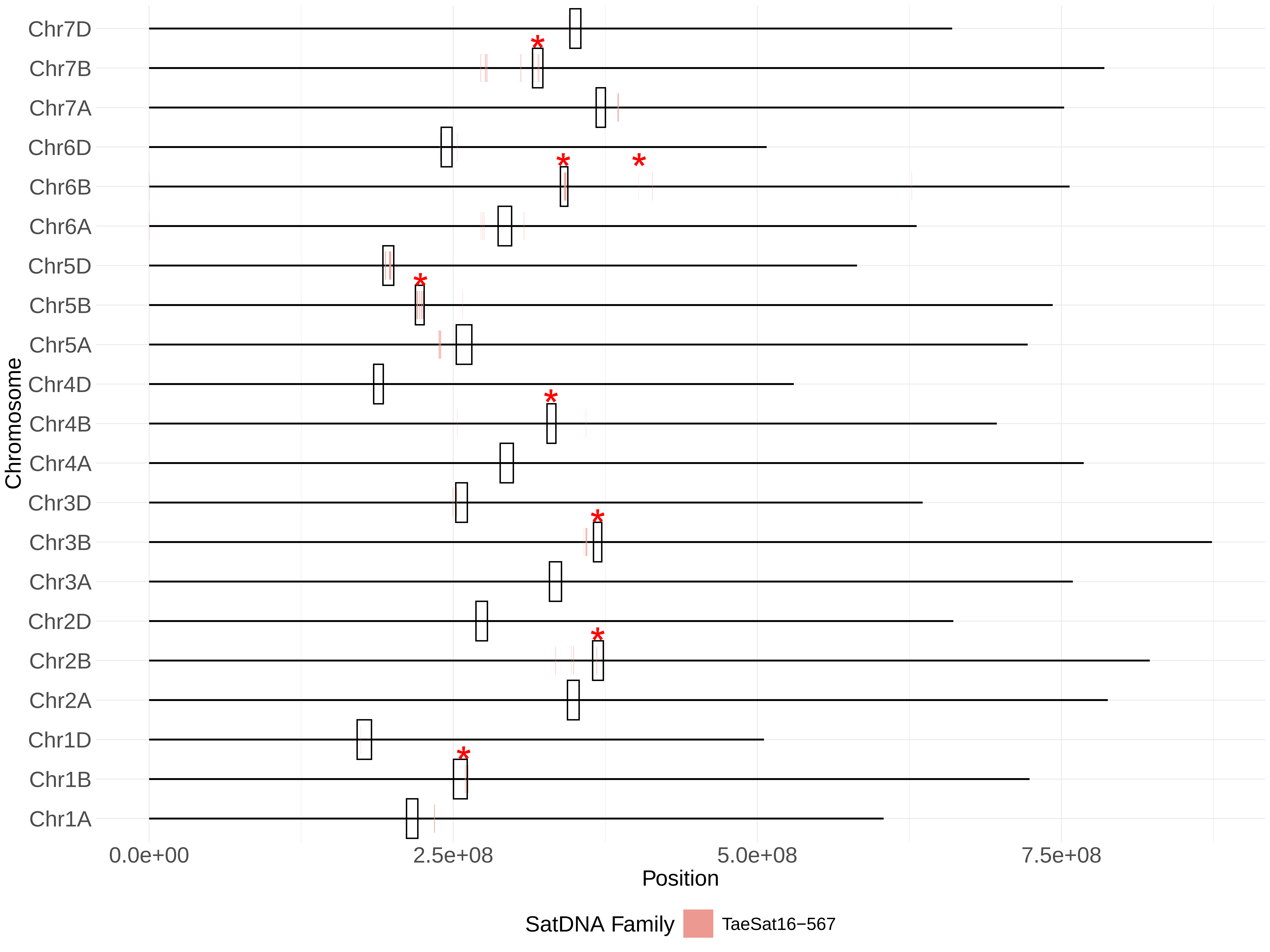
Chr7D
Chr7B
Chr7A
Chr6D
Chr6B
Chr6A
Chr5D
Chr5B
Chr5A
Chr4D
Chr4B
Chr4A
Chr3D
Chr3B
Chr3A
Chr2D
Chr2B
Chr2A
Chr1D
Chr1B
Chr1A
Chromosome
0.0e+00
2.5e+08
5.0e+08
7.5e+08
Position
SatDNA Family
TaeSat16−567

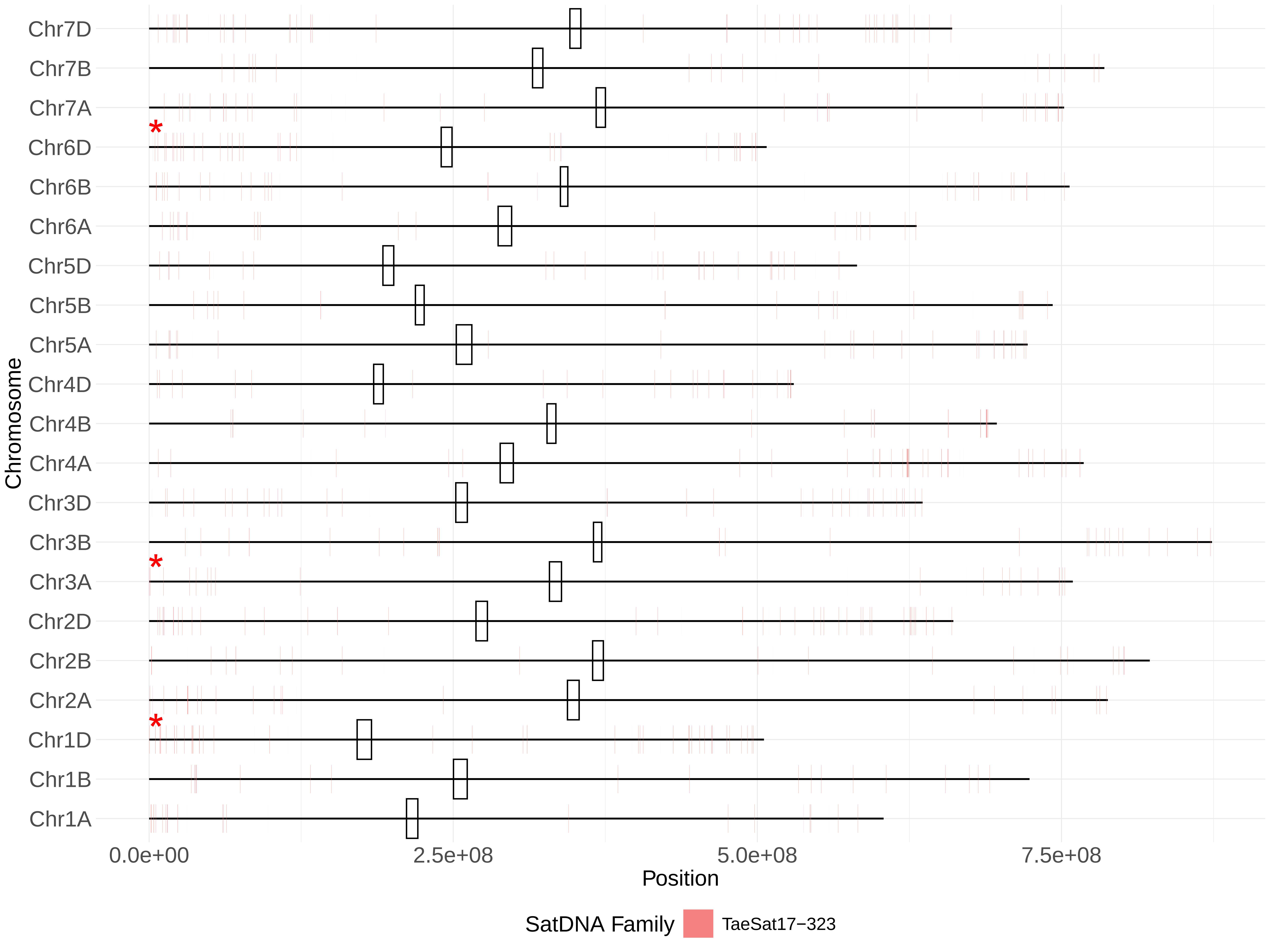

Chr7D
Chr7B
Chr7A
Chr6D
Chr6B
Chr6A
Chr5D
Chr5B
Chr5A
Chr4D
Chr4B
Chr4A
Chr3D
Chr3B
Chr3A
Chr2D
Chr2B
Chr2A
Chr1D
Chr1B
Chr1A
*
*
*
Chromosome
0.0e+00
2.5e+08
5.0e+08
7.5e+08
Position
SatDNA Family
TaeSat17−323

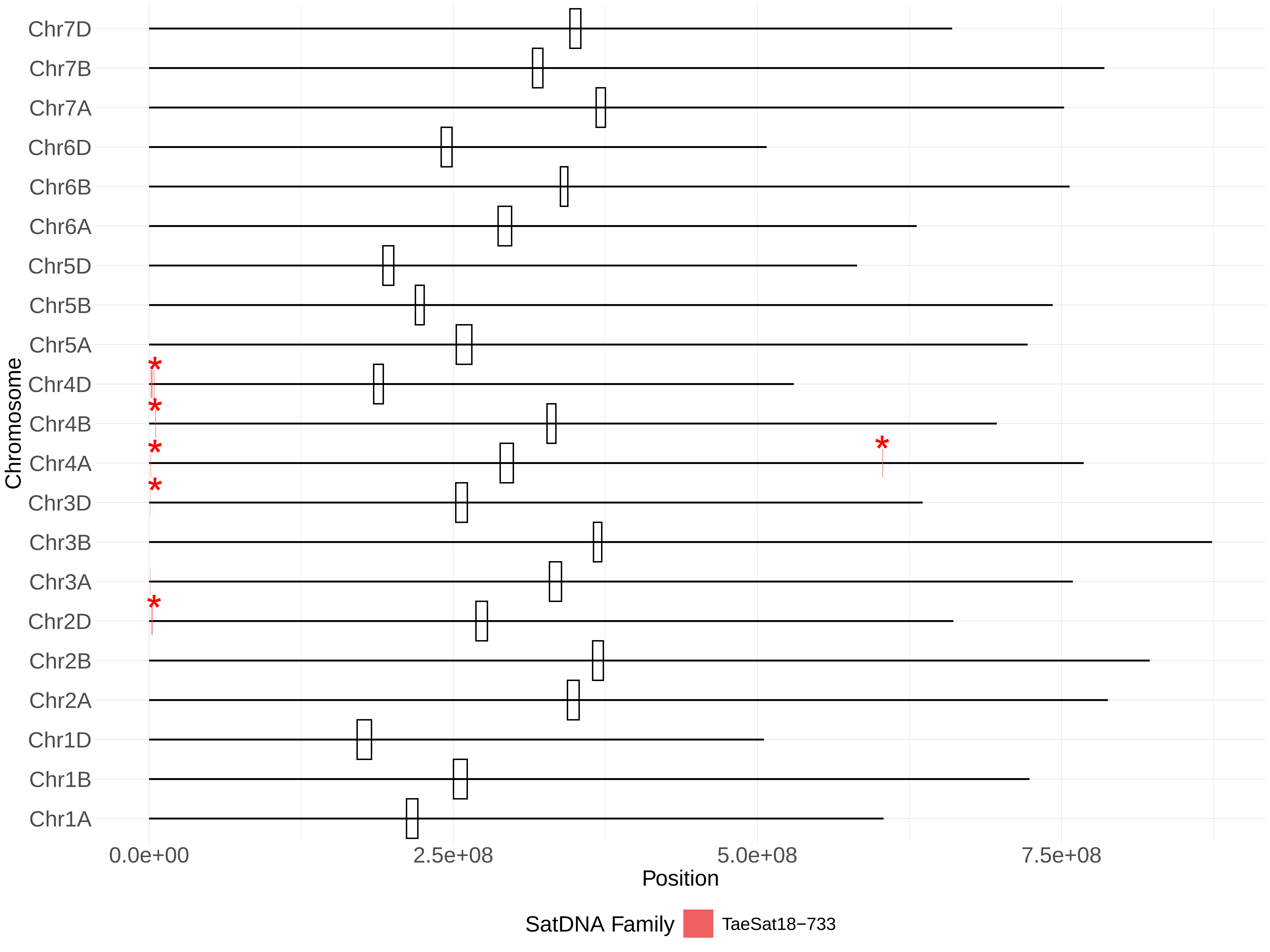

Chr7D
Chr7B
Chr7A
Chr6D
Chr6B
Chr6A
Chr5D
Chr5B
Chr5A
Chr4D
Chr4B
Chr4A
Chr3D
Chr3B
Chr3A
Chr2D
Chr2B
Chr2A
Chr1D
Chr1B
Chr1A
Chromosome
0.0e+00
2.5e+08
5.0e+08
7.5e+08
Position
SatDNA Family
TaeSat18−733

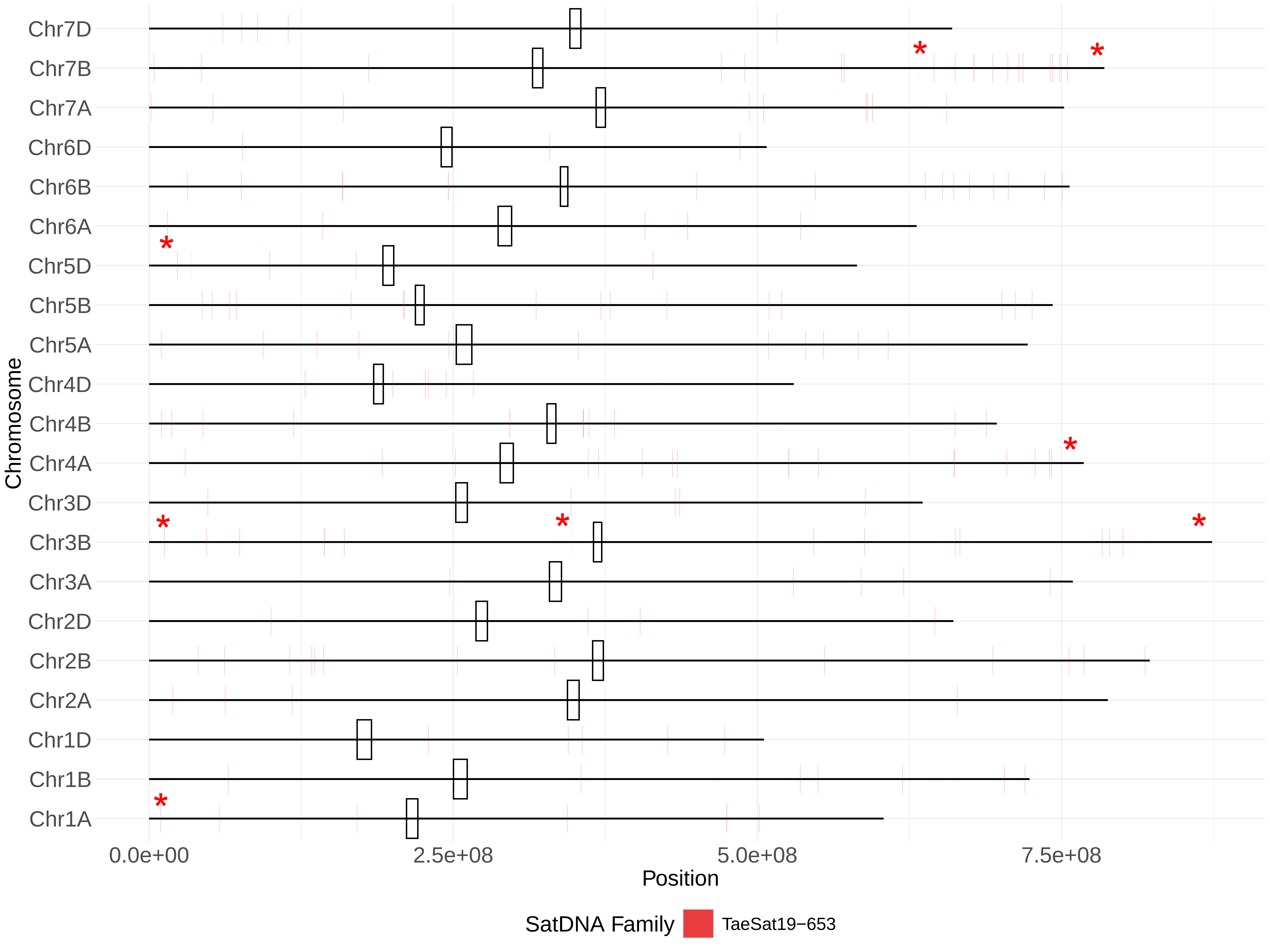
Chr7D
Chr7B
Chr7A
Chr6D
Chr6B
Chr6A
Chr5D
Chr5B
Chr5A
Chr4D
Chr4B
Chr4A
Chr3D
Chr3B
Chr3A
Chr2D
Chr2B
Chr2A
Chr1D
Chr1B
Chr1A
Chromosome
0.0e+00
2.5e+08
5.0e+08
7.5e+08
Position
SatDNA Family
TaeSat19−653

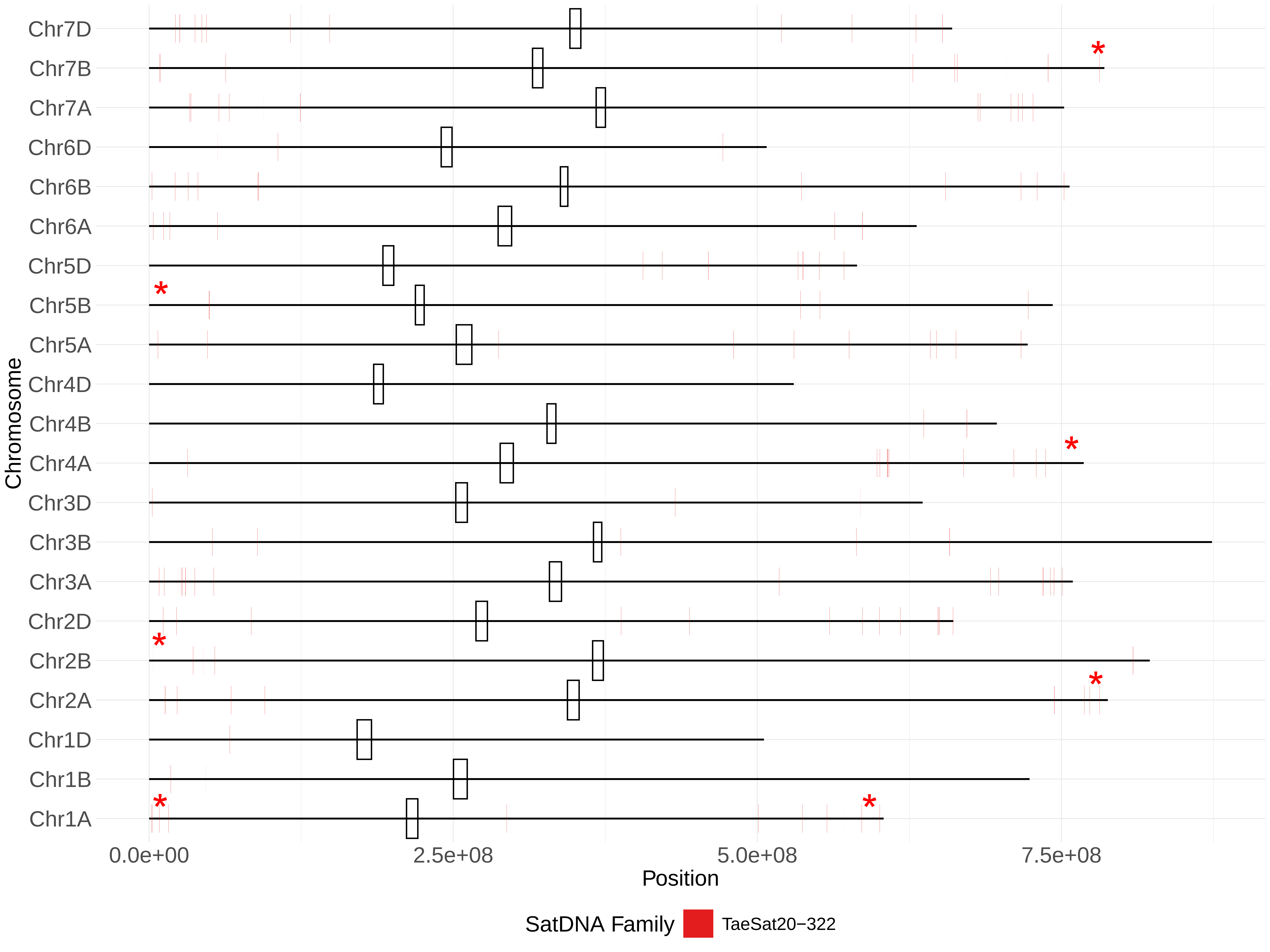

Chr7D
Chr7B
Chr7A
Chr6D
Chr6B
Chr6A
Chr5D
Chr5B
Chr5A
Chr4D
Chr4B
Chr4A
Chr3D
Chr3B
Chr3A
Chr2D
Chr2B
Chr2A
Chr1D
Chr1B
Chr1A
Chromosome
0.0e+00
2.5e+08
5.0e+08
7.5e+08
Position
SatDNA Family
TaeSat20−322

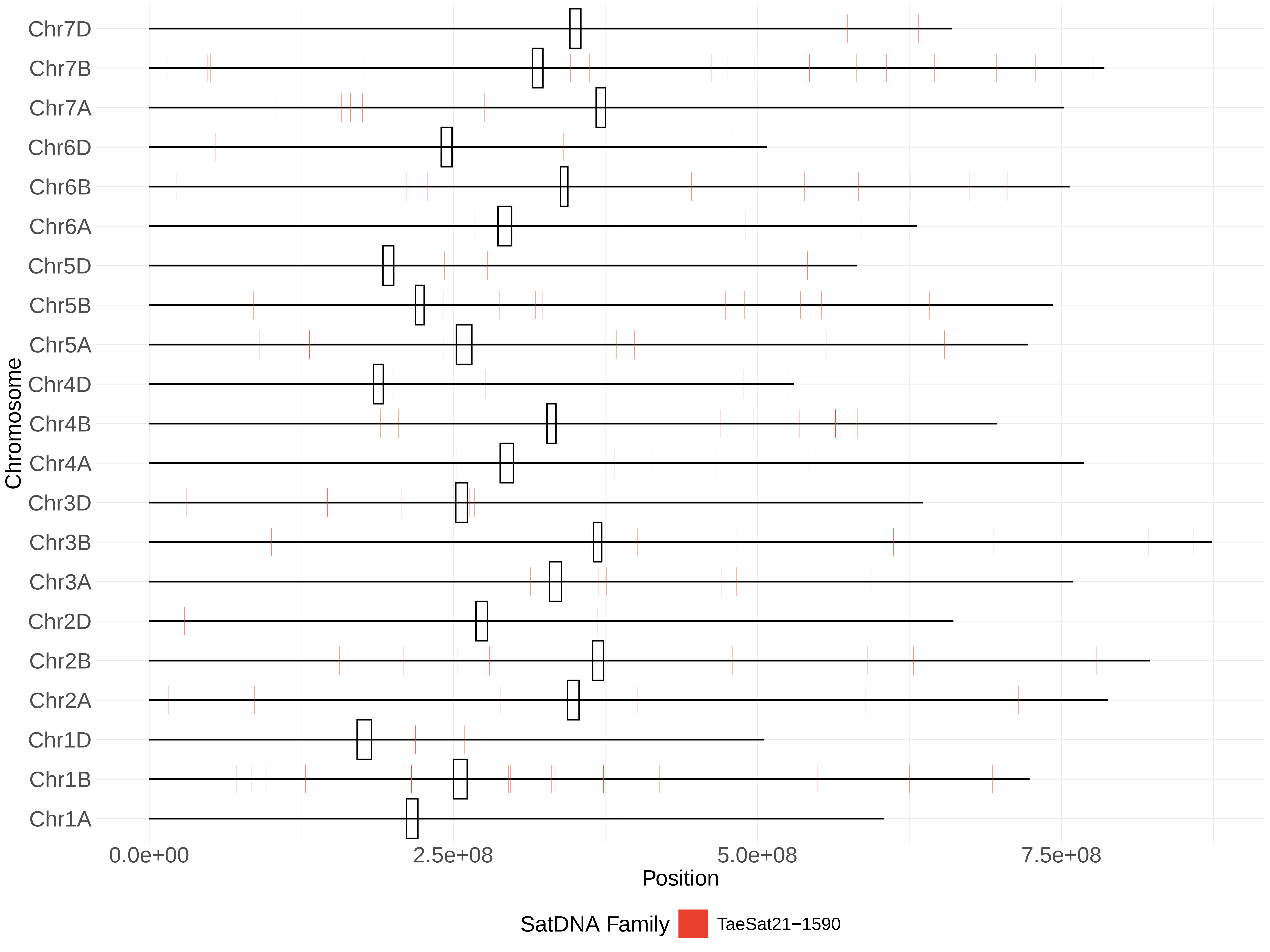
Chr7D
Chr7B
Chr7A
Chr6D
Chr6B
Chr6A
Chr5D
Chr5B
Chr5A
Chr4D
Chr4B
Chr4A
Chr3D
Chr3B
Chr3A
Chr2D
Chr2B
Chr2A
Chr1D
Chr1B
Chr1A
Chromosome
0.0e+00
2.5e+08
5.0e+08
7.5e+08
Position
SatDNA Family
TaeSat21−1590

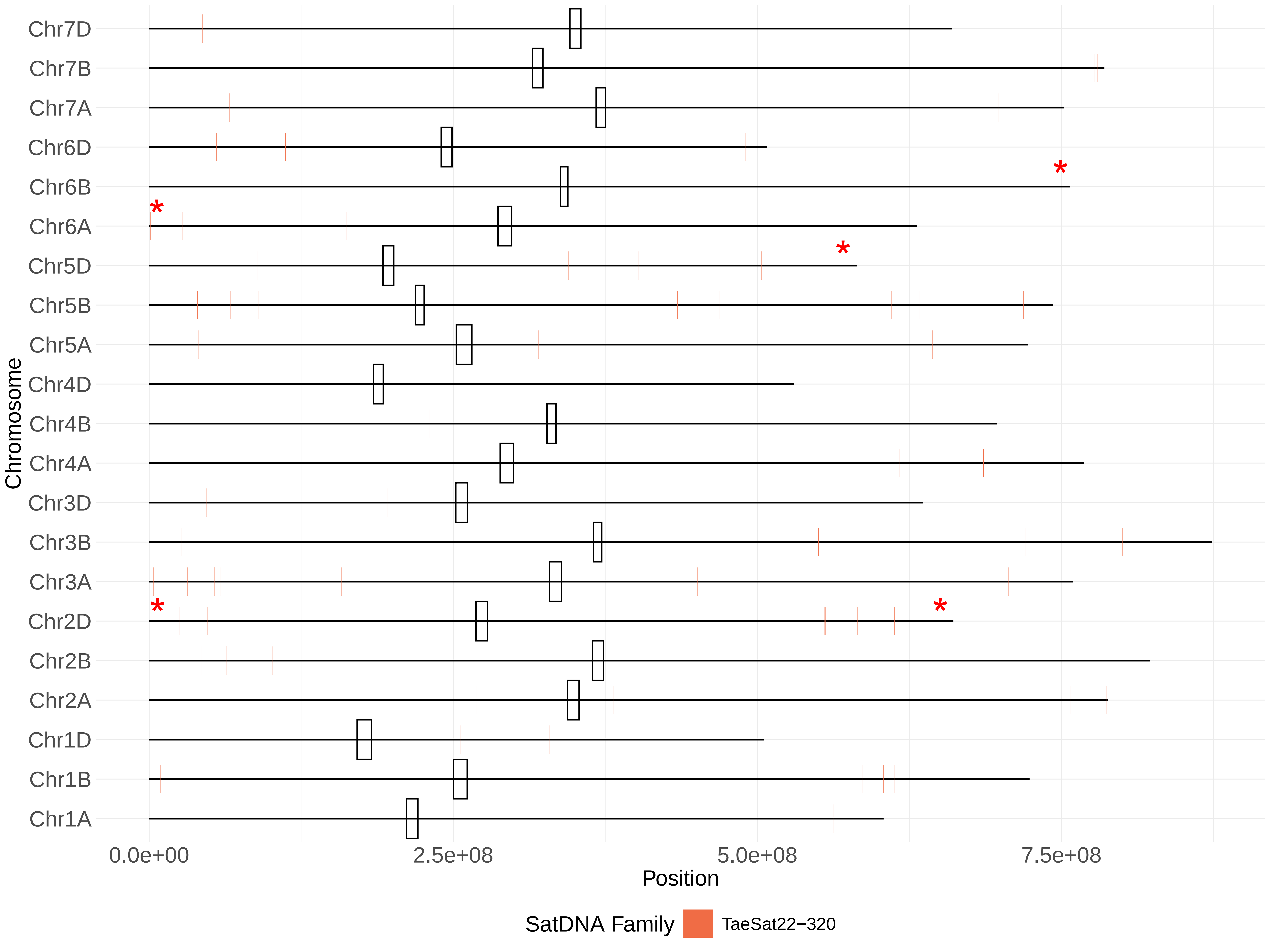

Chr7D
Chr7B
Chr7A
Chr6D
Chr6B
Chr6A
Chr5D
Chr5B
Chr5A
Chr4D
Chr4B
Chr4A
Chr3D
Chr3B
Chr3A
Chr2D
Chr2B
Chr2A
Chr1D
Chr1B
Chr1A
Chromosome
0.0e+00
2.5e+08
5.0e+08
7.5e+08
Position
SatDNA Family
TaeSat22−320

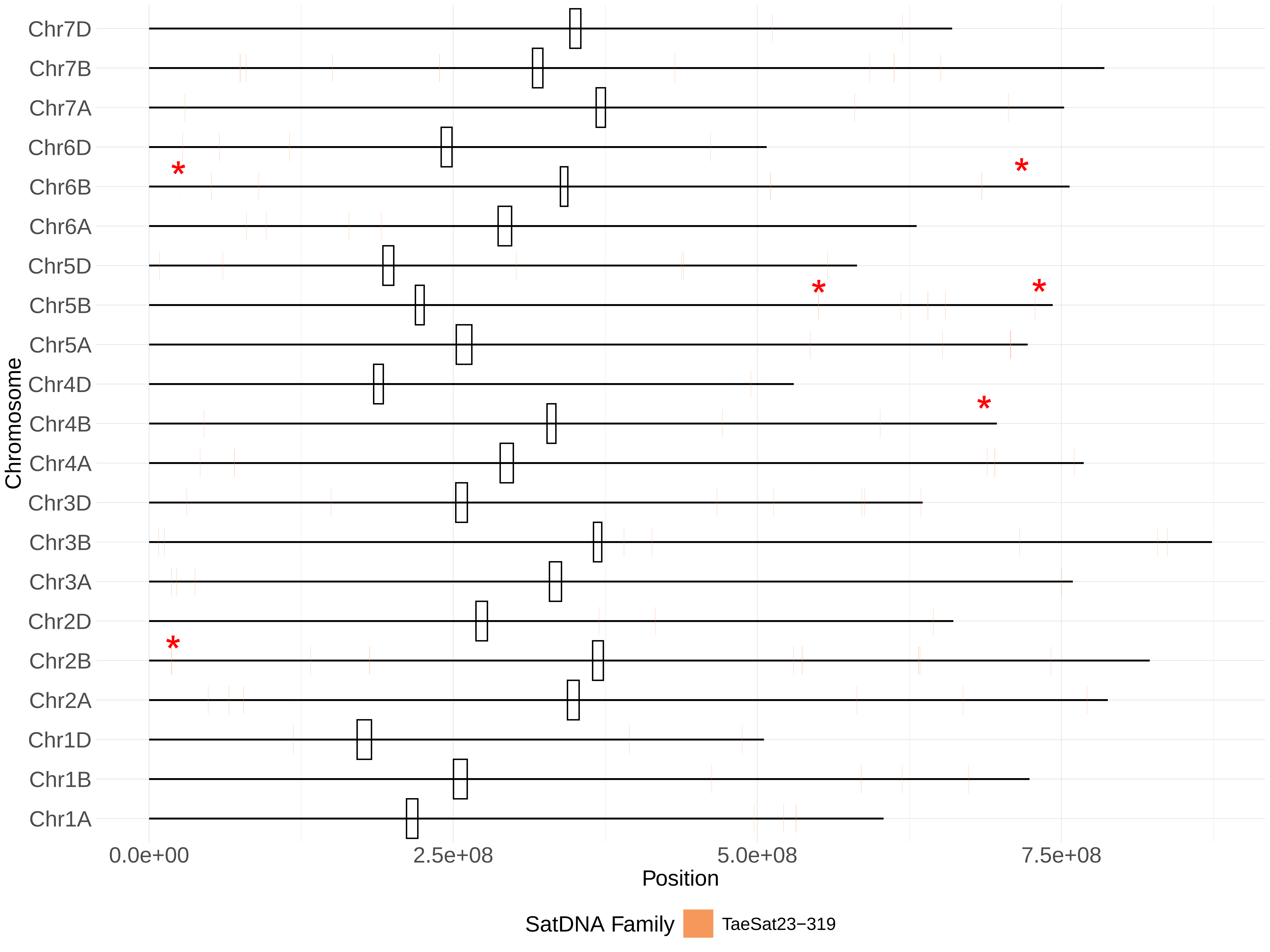

Chr7D
Chr7B
Chr7A
Chr6D
Chr6B
Chr6A
Chr5D
Chr5B
Chr5A
Chr4D
Chr4B
Chr4A
Chr3D
Chr3B
Chr3A
Chr2D
Chr2B
Chr2A
Chr1D
Chr1B
Chr1A
Chromosome
0.0e+00
2.5e+08
5.0e+08
7.5e+08
Position
SatDNA Family
TaeSat23−319

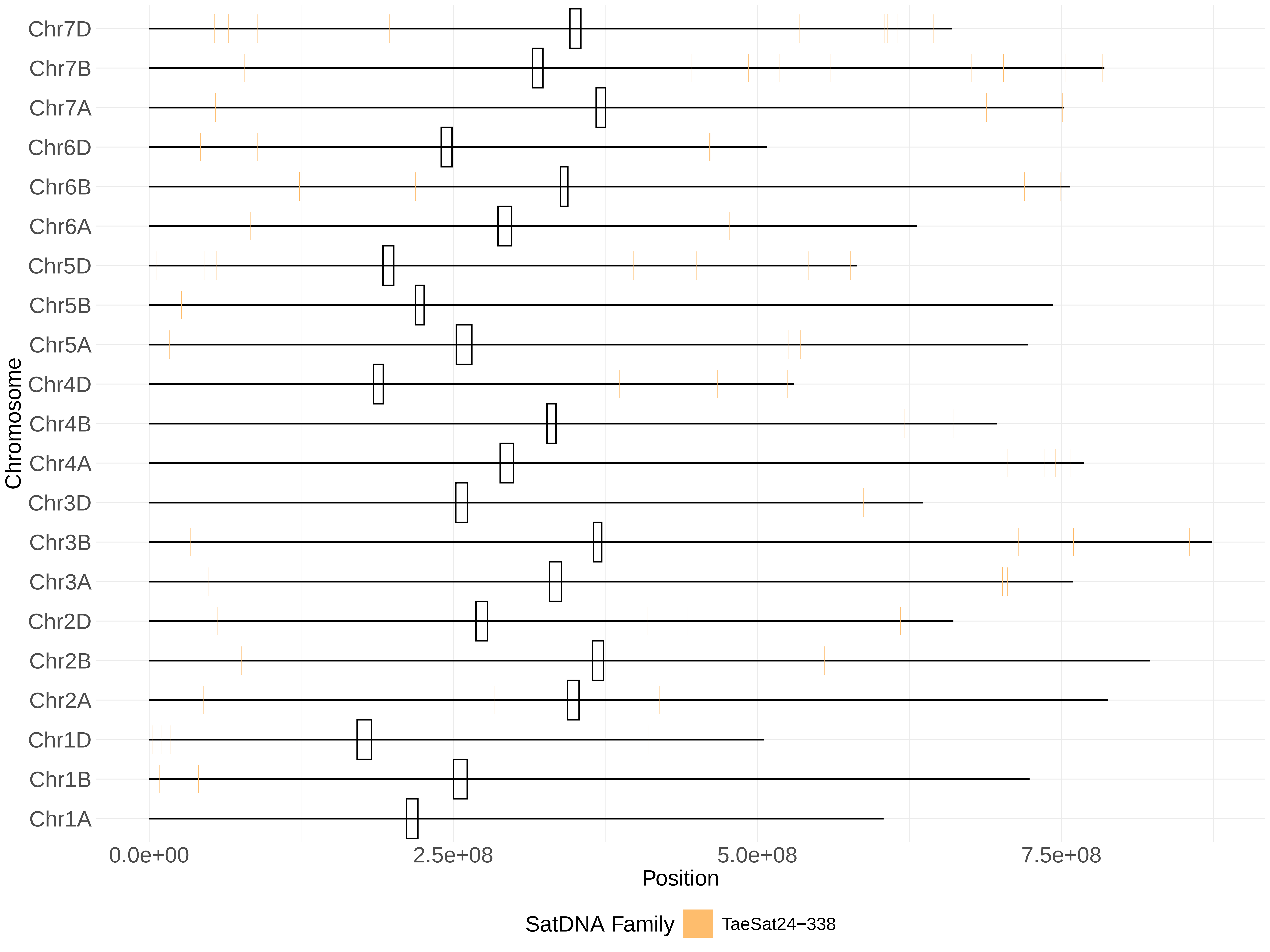
Chr7D
Chr7B
Chr7A
Chr6D
Chr6B
Chr6A
Chr5D
Chr5B
Chr5A
Chr4D
Chr4B
Chr4A
Chr3D
Chr3B
Chr3A
Chr2D
Chr2B
Chr2A
Chr1D
Chr1B
Chr1A
Chromosome
0.0e+00
2.5e+08
5.0e+08
7.5e+08
Position
SatDNA Family
TaeSat24−338

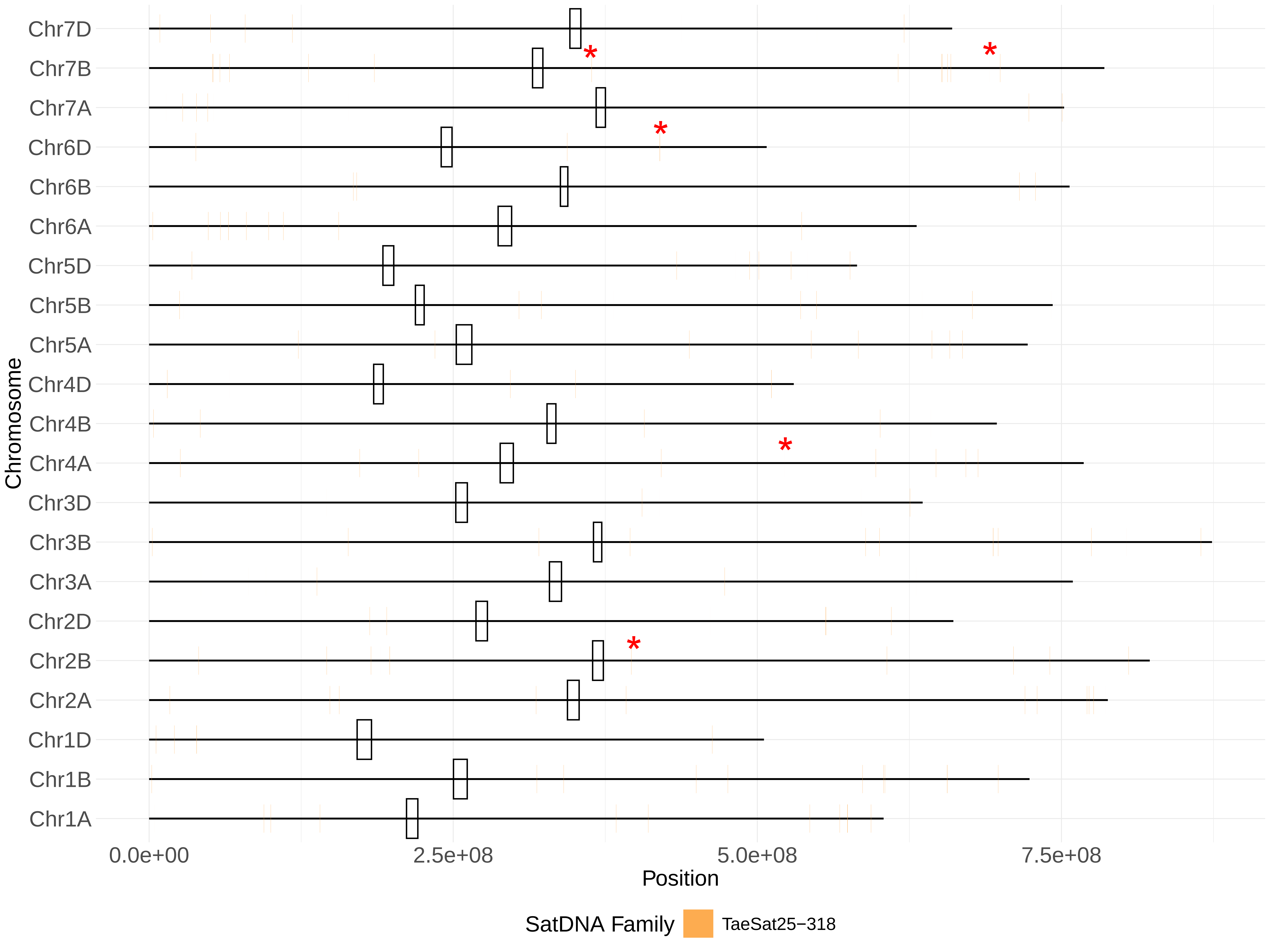

Chr7D
Chr7B
Chr7A
Chr6D
Chr6B
Chr6A
Chr5D
Chr5B
Chr5A
Chr4D
Chr4B
Chr4A
Chr3D
Chr3B
Chr3A
Chr2D
Chr2B
Chr2A
Chr1D
Chr1B
Chr1A
Chromosome
0.0e+00
2.5e+08
5.0e+08
7.5e+08
Position
SatDNA Family
TaeSat25−318

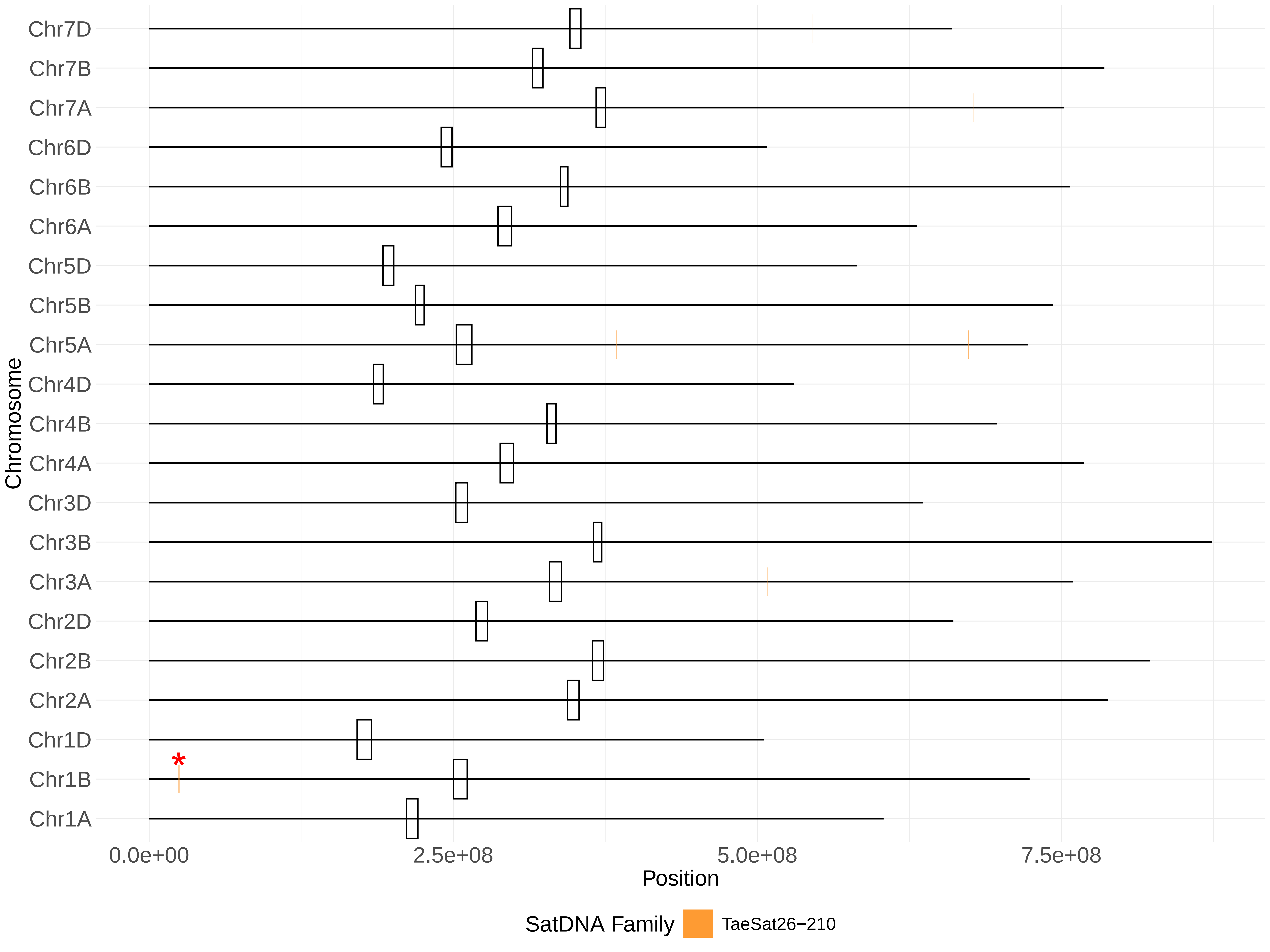

Chr7D
Chr7B
Chr7A
Chr6D
Chr6B
Chr6A
Chr5D
Chr5B
Chr5A
Chr4D
Chr4B
Chr4A
Chr3D
Chr3B
Chr3A
Chr2D
Chr2B
Chr2A
Chr1D
Chr1B
Chr1A
Chromosome
*
0.0e+00
2.5e+08
5.0e+08
7.5e+08
Position
SatDNA Family
TaeSat26−210

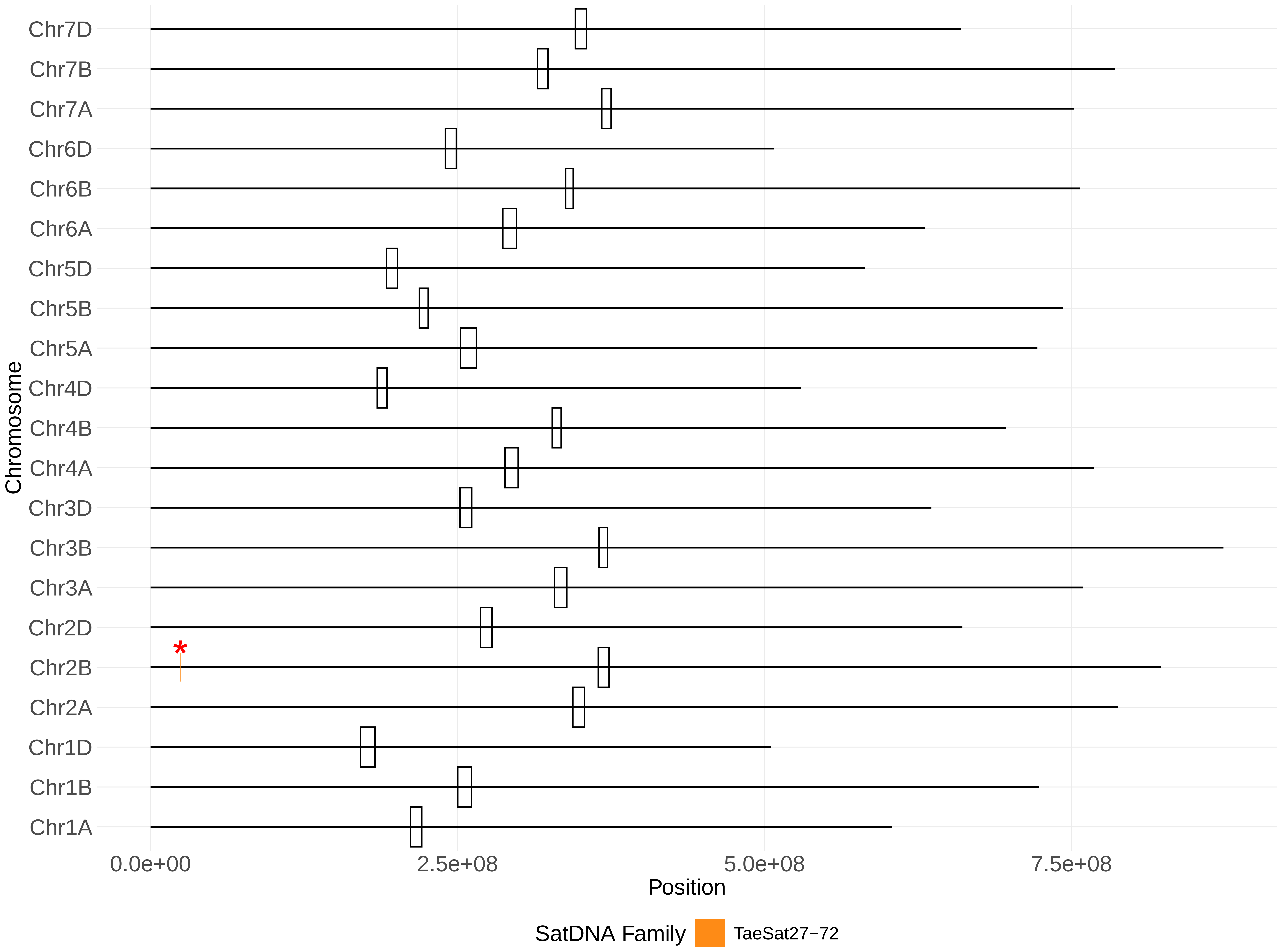
Chr7D
Chr7B
Chr7A
Chr6D
Chr6B
Chr6A
Chr5D
Chr5B
Chr5A
Chr4D
Chr4B
Chr4A
Chr3D
Chr3B
Chr3A
Chr2D
Chr2B
Chr2A
Chr1D
Chr1B
Chr1A
Chromosome
0.0e+00
2.5e+08
5.0e+08
7.5e+08
Position
*
SatDNA Family
TaeSat27−72

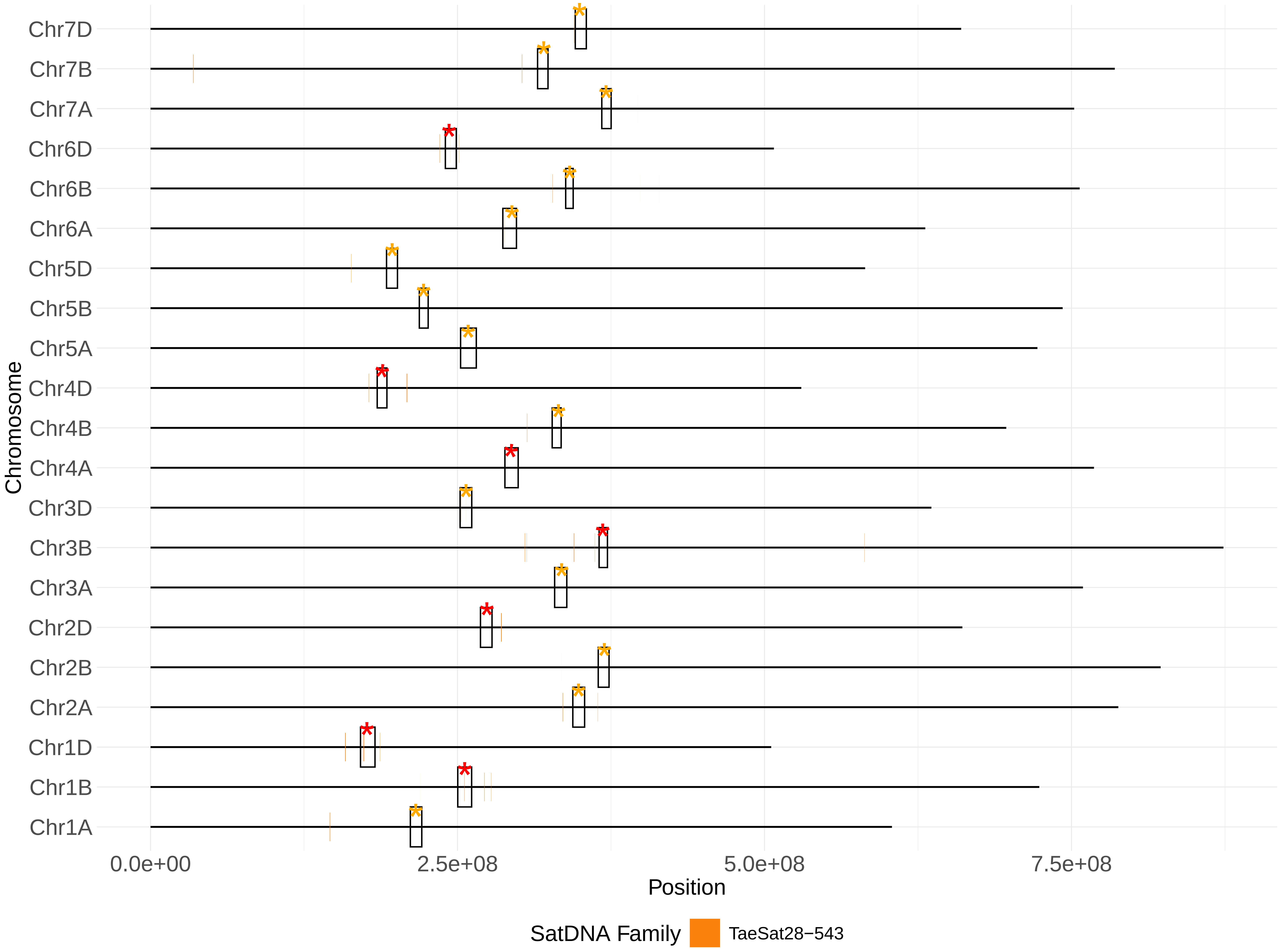

Chr7D
Chr7B
Chr7A
Chr6D
Chr6B
Chr6A
Chr5D
Chr5B
Chr5A
Chr4D
Chr4B
Chr4A
Chr3D
Chr3B
Chr3A
Chr2D
Chr2B
Chr2A
Chr1D
Chr1B
Chr1A
Chromosome
0.0e+00
2.5e+08
5.0e+08
7.5e+08
Position
SatDNA Family
TaeSat28−543

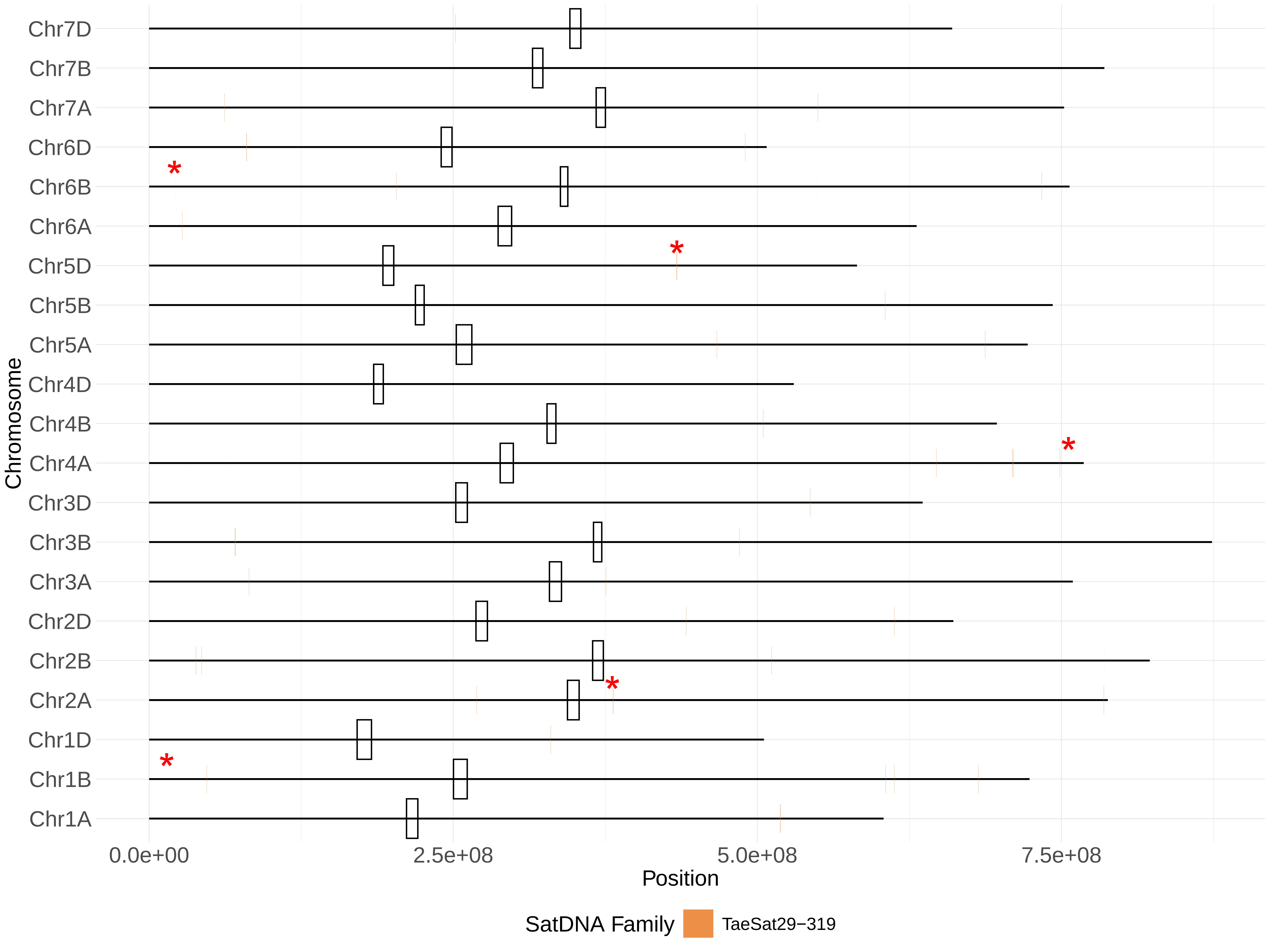
Chr7D
Chr7B
Chr7A
Chr6D
Chr6B
Chr6A
Chr5D
Chr5B
Chr5A
Chr4D
Chr4B
Chr4A
Chr3D
Chr3B
Chr3A
Chr2D
Chr2B
Chr2A
Chr1D
Chr1B
Chr1A
Chromosome
0.0e+00
2.5e+08
5.0e+08
7.5e+08
Position
SatDNA Family
TaeSat29−319

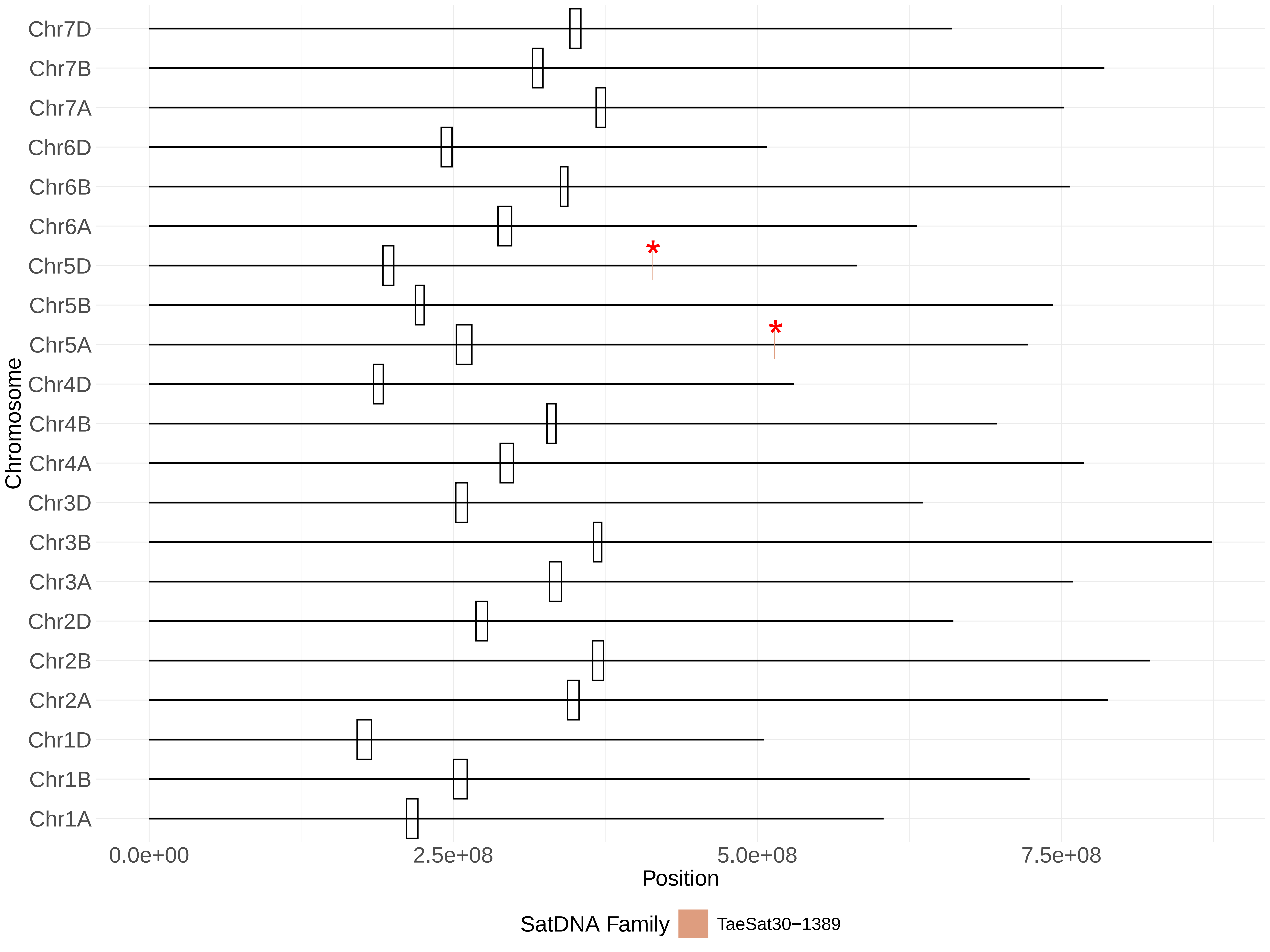

Chr7D
Chr7B
Chr7A
Chr6D
Chr6B
Chr6A
Chr5D
Chr5B
Chr5A
Chr4D
Chr4B
Chr4A
Chr3D
Chr3B
Chr3A
Chr2D
Chr2B
Chr2A
Chr1D
Chr1B
Chr1A
Chromosome
0.0e+00
2.5e+08
5.0e+08
7.5e+08
Position
SatDNA Family
TaeSat30−1389

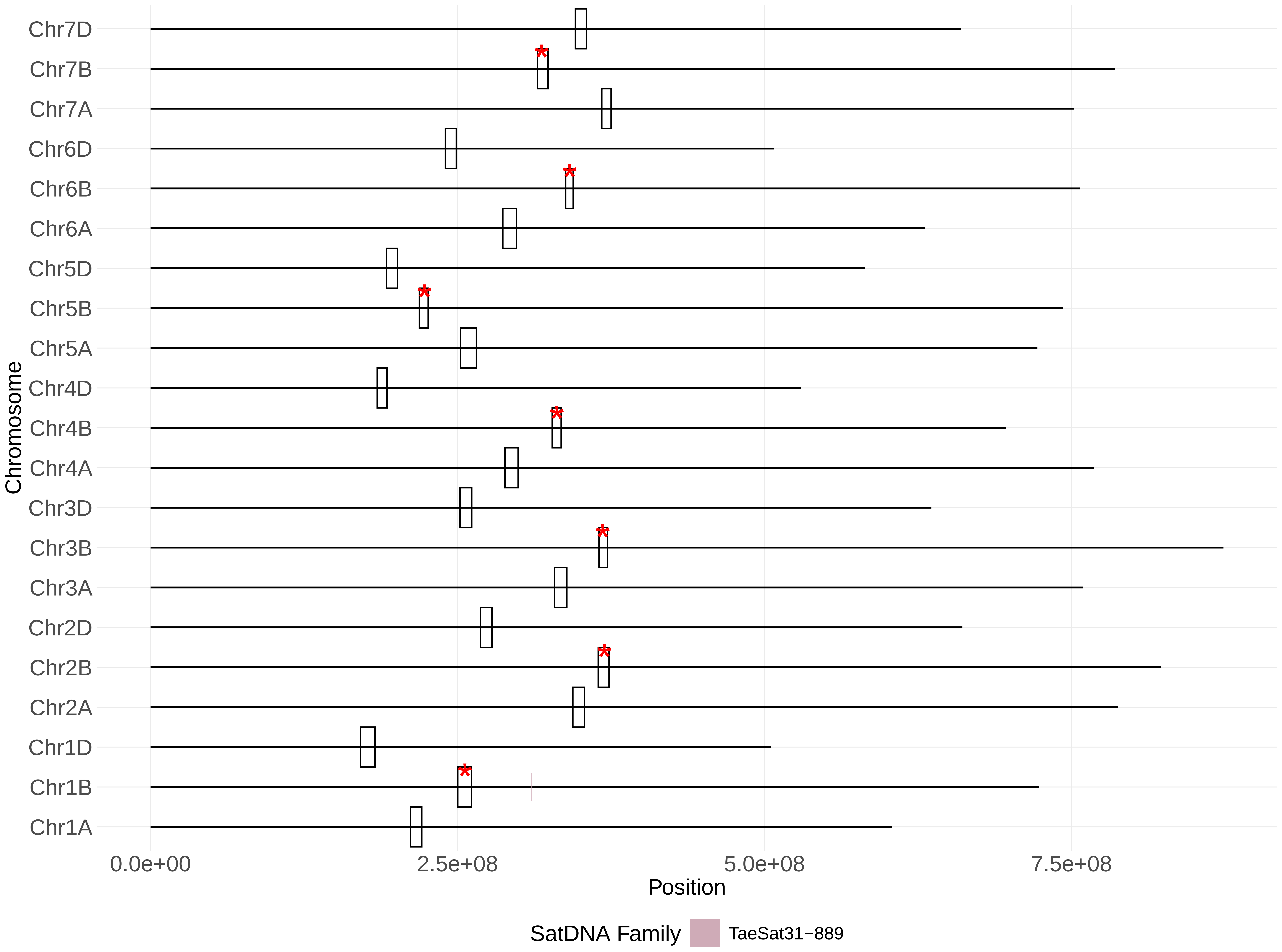
Chr7D
Chr7B
Chr7A
Chr6D
Chr6B
Chr6A
Chr5D
Chr5B
Chr5A
Chr4D
Chr4B
Chr4A
Chr3D
Chr3B
Chr3A
Chr2D
Chr2B
Chr2A
Chr1D
Chr1B
Chr1A
Chromosome
0.0e+00
2.5e+08
5.0e+08
7.5e+08
Position
SatDNA Family
TaeSat31−889

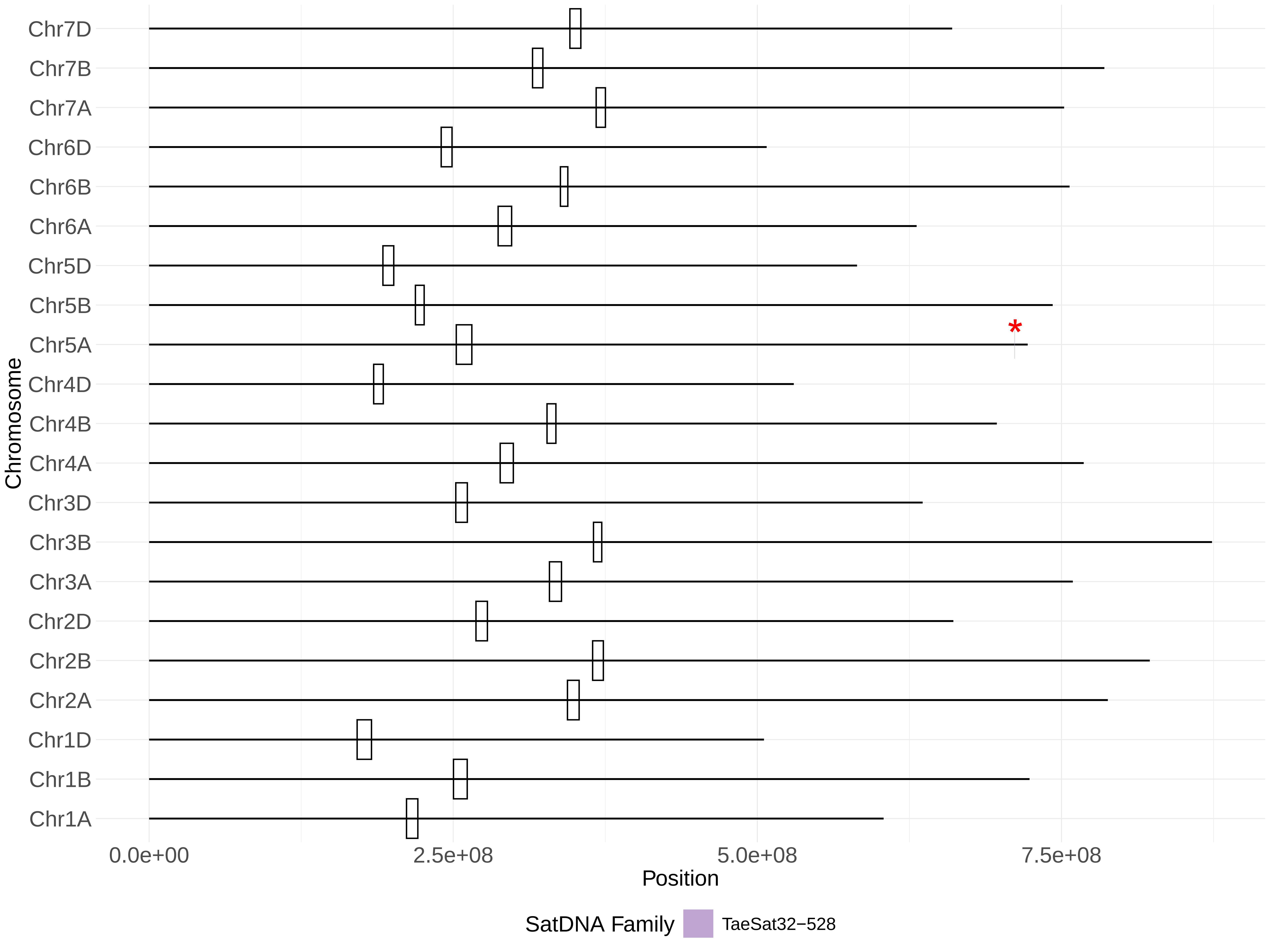
Chr7D
Chr7B
Chr7A
Chr6D
Chr6B
Chr6A
Chr5D
Chr5B
Chr5A
Chr4D
Chr4B
Chr4A
Chr3D
Chr3B
Chr3A
Chr2D
Chr2B
Chr2A
Chr1D
Chr1B
Chr1A
Chromosome
*
0.0e+00
2.5e+08
5.0e+08
7.5e+08
Position
SatDNA Family
TaeSat32−528

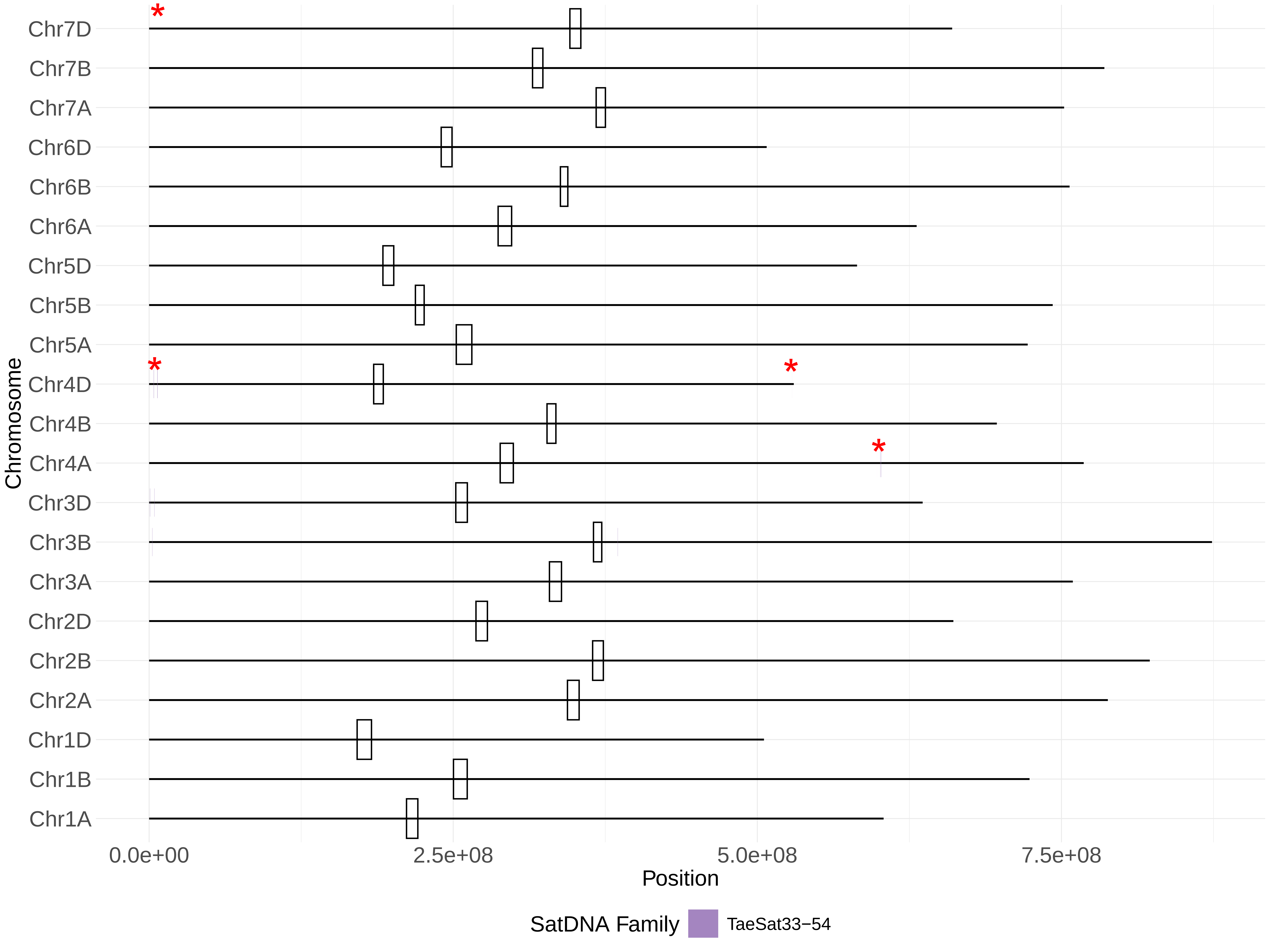
Chr7D
Chr7B
Chr7A
Chr6D
Chr6B
Chr6A
Chr5D
Chr5B
Chr5A
Chr4D
Chr4B
Chr4A
Chr3D
Chr3B
Chr3A
Chr2D
Chr2B
Chr2A
Chr1D
Chr1B
Chr1A
Chromosome
0.0e+00
2.5e+08
5.0e+08
7.5e+08
Position
SatDNA Family
TaeSat33−54

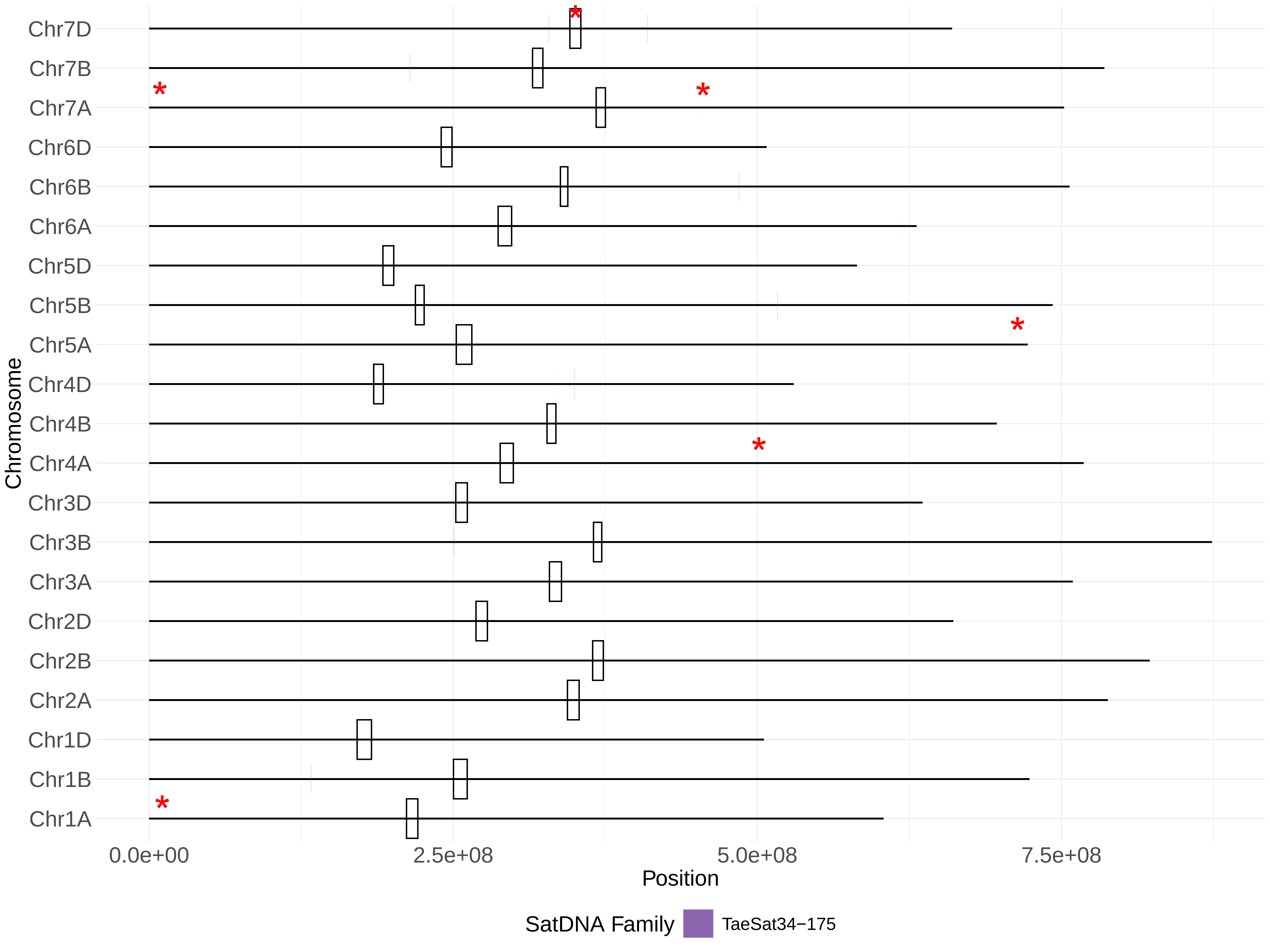
Chr7D
Chr7B
Chr7A
Chr6D
Chr6B
Chr6A
Chr5D
Chr5B
Chr5A
Chr4D
Chr4B
Chr4A
Chr3D
Chr3B
Chr3A
Chr2D
Chr2B
Chr2A
Chr1D
Chr1B
Chr1A
Chromosome
0.0e+00
2.5e+08
5.0e+08
7.5e+08
Position
SatDNA Family
TaeSat34−175

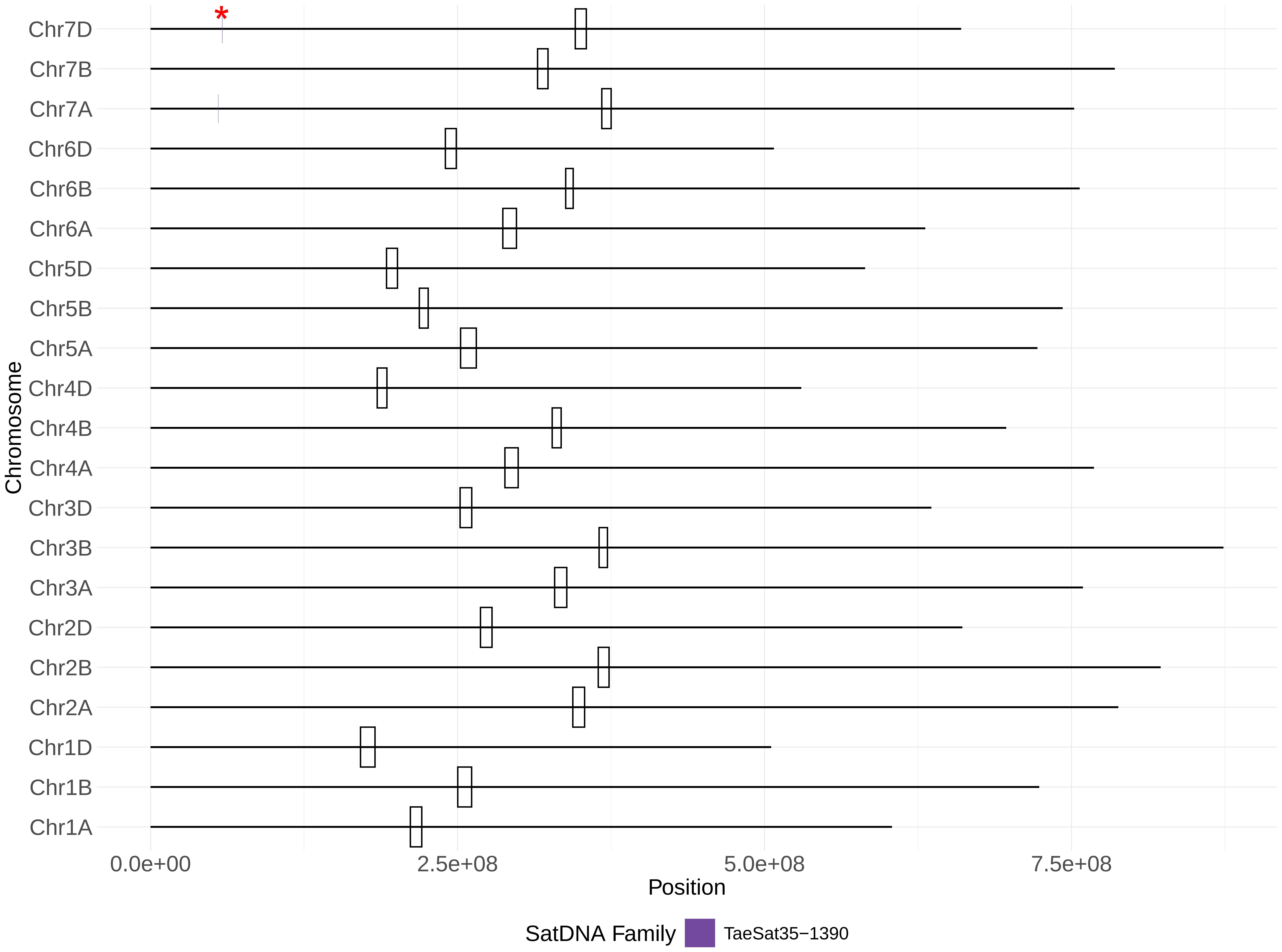
*
Chr7D
Chr7B
Chr7A
Chr6D
Chr6B
Chr6A
Chr5D
Chr5B
Chr5A
Chr4D
Chr4B
Chr4A
Chr3D
Chr3B
Chr3A
Chr2D
Chr2B
Chr2A
Chr1D
Chr1B
Chr1A
Chromosome
0.0e+00
2.5e+08
5.0e+08
7.5e+08
Position
SatDNA Family
TaeSat35−1390

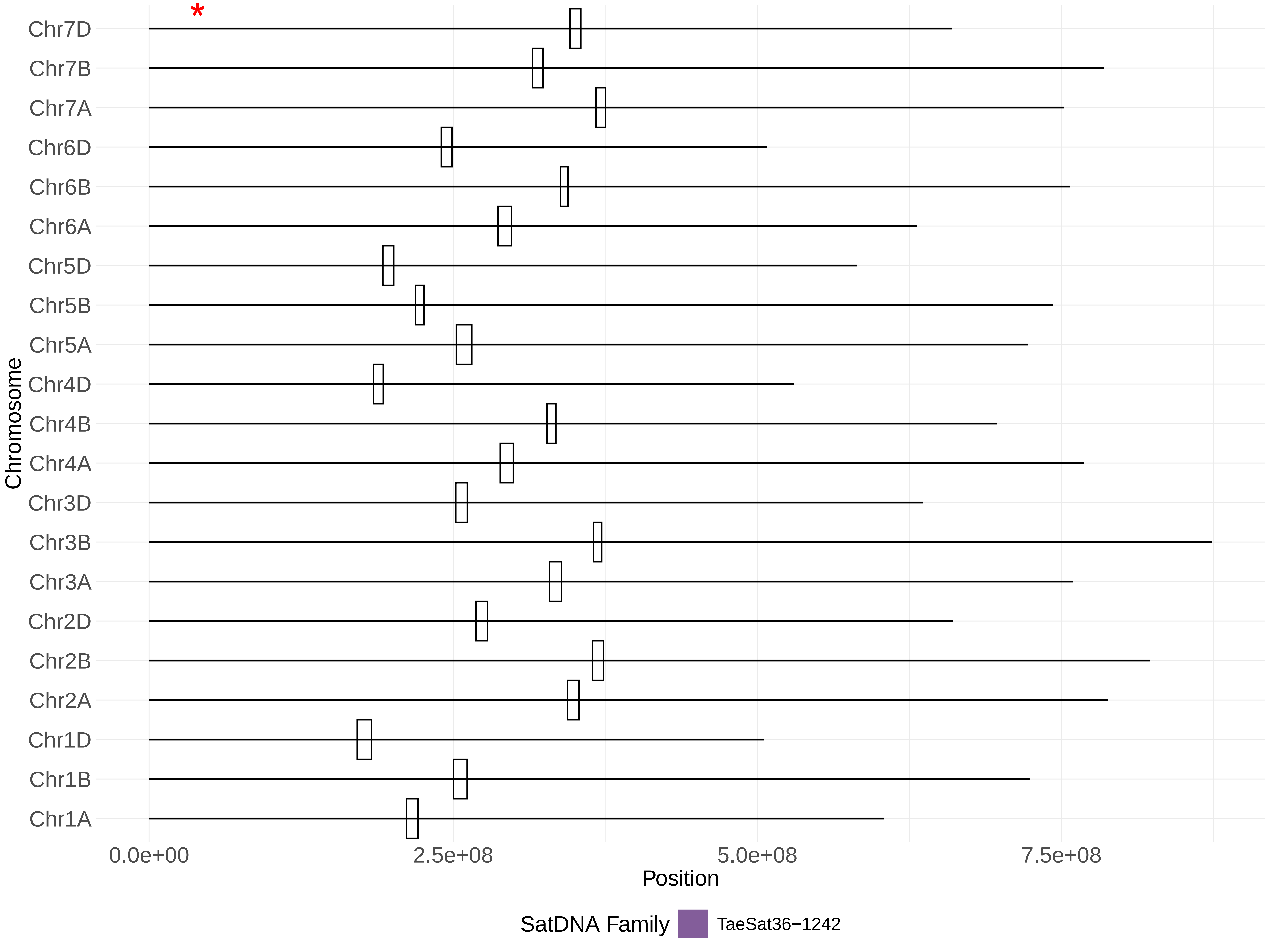
*
Chr7D
Chr7B
Chr7A
Chr6D
Chr6B
Chr6A
Chr5D
Chr5B
Chr5A
Chr4D
Chr4B
Chr4A
Chr3D
Chr3B
Chr3A
Chr2D
Chr2B
Chr2A
Chr1D
Chr1B
Chr1A
Chromosome
0.0e+00
2.5e+08
5.0e+08
7.5e+08
Position
SatDNA Family
TaeSat36−1242
