# Supplementary material for: Detailed re-analysis of satellitome mapping facilitated by the telomere-to-telomere (T2T) assembly of bread wheat genome
Source: Plant Mol Biol. 2026 Aug 28;116(5):92. doi: 10.1007/s11103-026-01745-3 (PMC13525055; doi:10.1007/s11103-026-01745-3)
Supplement: Supplementary file 3 [file 11103_2026_1745_MOESM3_ESM.pdf]

Supplementary Figure 2

Detailed re-analysis of satellitome mapping facilitated by the telomere-to-telomere (T2T) assembly of bread wheat genome

Manuel A. Garrido-Ramos and Pilar Prieto

Figure S2. *T. aestivum* cv. Chinese Spring centromeres representation showing the distribution of different satDNA families individually represented (a page per satDNA) as obtained through the CHRISMAPP approach.

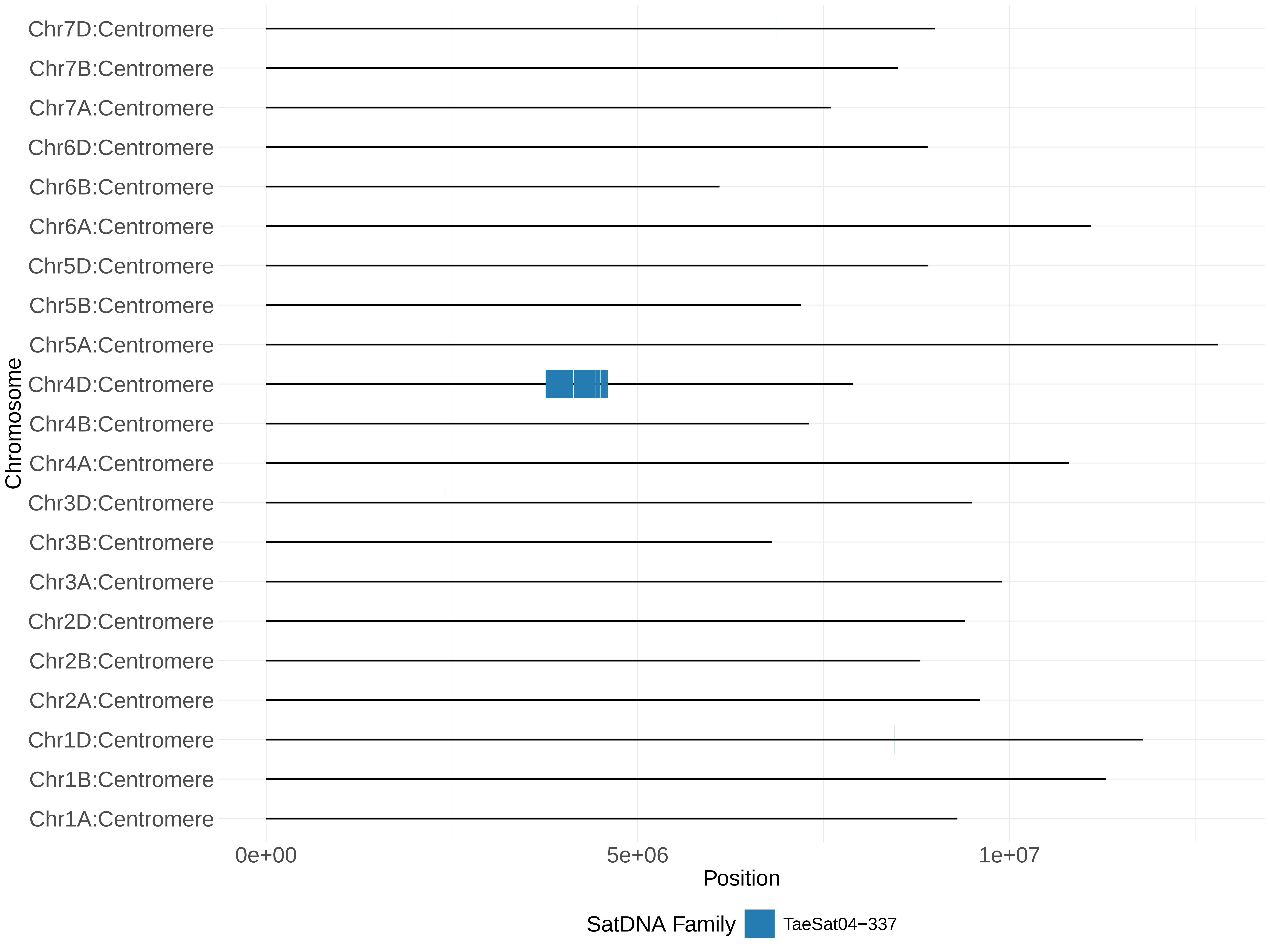

Chr7D:Centromere
Chr7B:Centromere
Chr7A:Centromere
Chr6D:Centromere
Chr6B:Centromere
Chr6A:Centromere
Chr5D:Centromere
Chr5B:Centromere
Chr5A:Centromere
Chr4D:Centromere
Chr4B:Centromere
Chr4A:Centromere
Chr3D:Centromere
Chr3B:Centromere
Chr3A:Centromere
Chr2D:Centromere
Chr2B:Centromere
Chr2A:Centromere
Chr1D:Centromere
Chr1B:Centromere
Chr1A:Centromere
Chromosome
0e+00
5e+06
1e+07
Position
SatDNA Family
TaeSat04−337

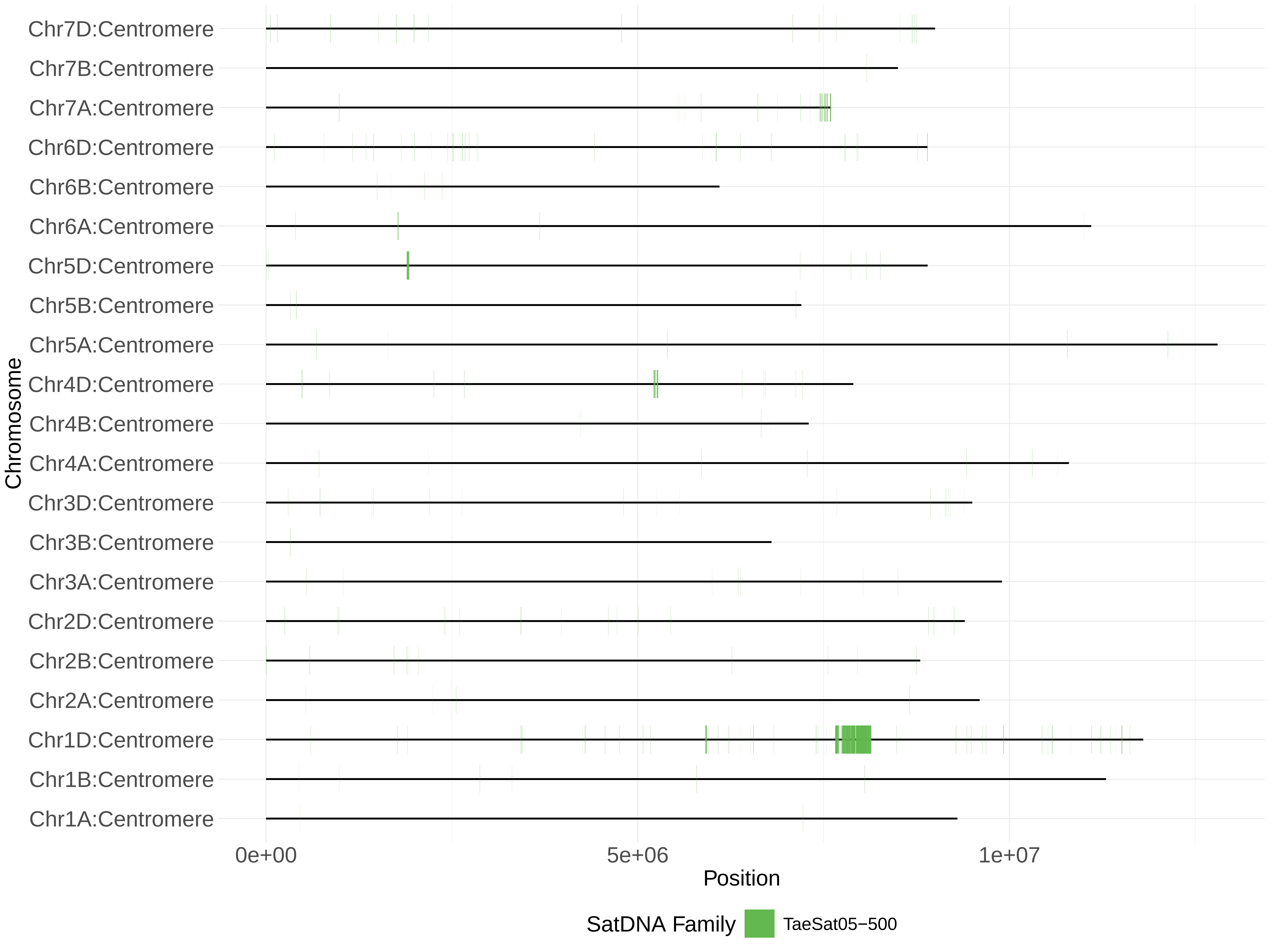

Chr7D:Centromere
Chr7B:Centromere
Chr7A:Centromere
Chr6D:Centromere
Chr6B:Centromere
Chr6A:Centromere
Chr5D:Centromere
Chr5B:Centromere
Chr5A:Centromere
Chr4D:Centromere
Chr4B:Centromere
Chr4A:Centromere
Chr3D:Centromere
Chr3B:Centromere
Chr3A:Centromere
Chr2D:Centromere
Chr2B:Centromere
Chr2A:Centromere
Chr1D:Centromere
Chr1B:Centromere
Chr1A:Centromere
Chromosome
0e+00
5e+06
1e+07
Position
SatDNA Family
TaeSat05−500

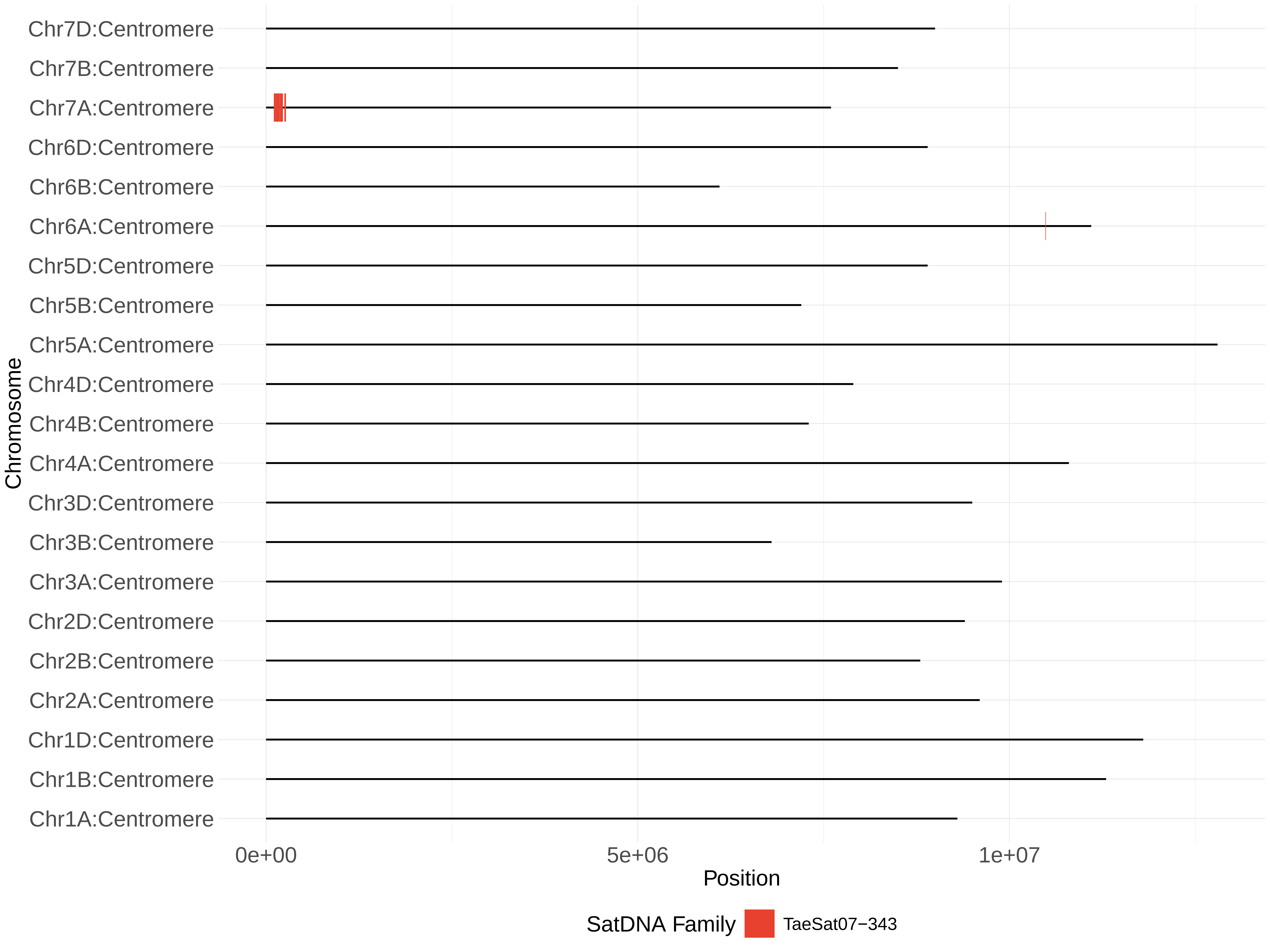

Chr7D:Centromere
Chr7B:Centromere
Chr7A:Centromere
Chr6D:Centromere
Chr6B:Centromere
Chr6A:Centromere
Chr5D:Centromere
Chr5B:Centromere
Chr5A:Centromere
Chr4D:Centromere
Chr4B:Centromere
Chr4A:Centromere
Chr3D:Centromere
Chr3B:Centromere
Chr3A:Centromere
Chr2D:Centromere
Chr2B:Centromere
Chr2A:Centromere
Chr1D:Centromere
Chr1B:Centromere
Chr1A:Centromere
Chromosome
0e+00
5e+06
1e+07
Position
SatDNA Family
TaeSat07−343

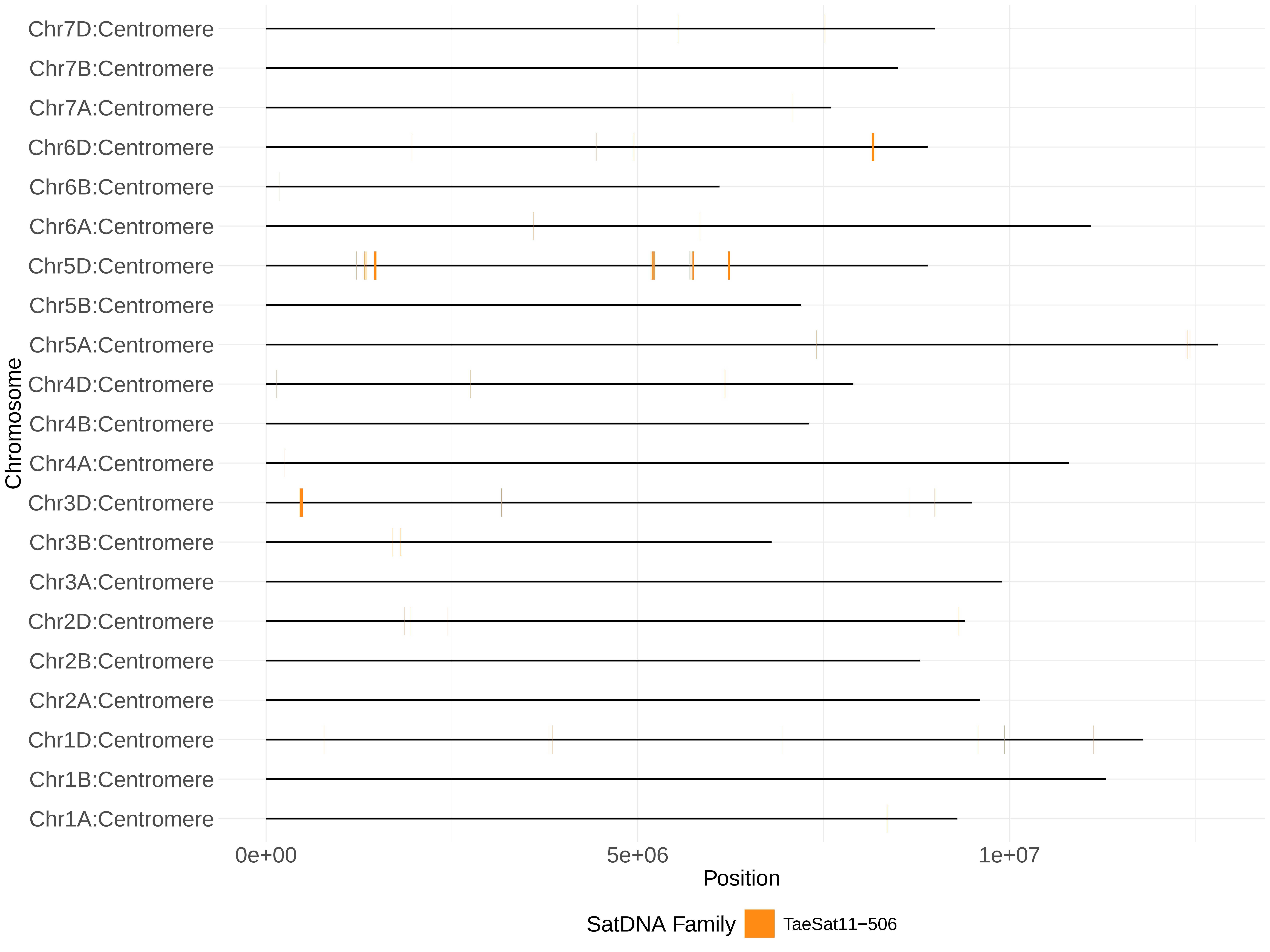

Chr7D:Centromere
Chr7B:Centromere
Chr7A:Centromere
Chr6D:Centromere
Chr6B:Centromere
Chr6A:Centromere
Chr5D:Centromere
Chr5B:Centromere
Chr5A:Centromere
Chr4D:Centromere
Chr4B:Centromere
Chr4A:Centromere
Chr3D:Centromere
Chr3B:Centromere
Chr3A:Centromere
Chr2D:Centromere
Chr2B:Centromere
Chr2A:Centromere
Chr1D:Centromere
Chr1B:Centromere
Chr1A:Centromere
Chromosome
0e+00
5e+06
1e+07
Position
SatDNA Family
TaeSat11−506

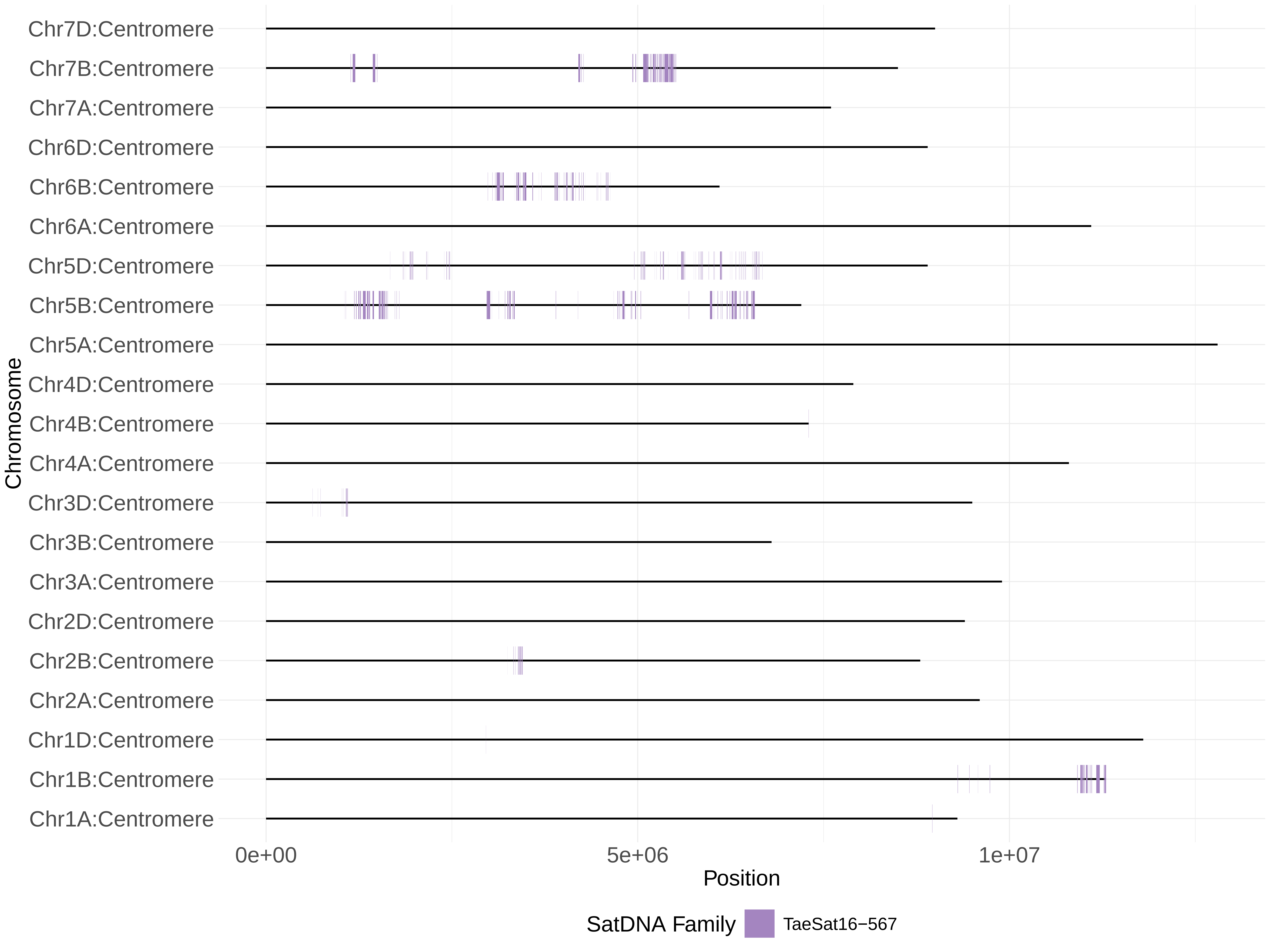

Chr7D:Centromere
Chr7B:Centromere
Chr7A:Centromere
Chr6D:Centromere
Chr6B:Centromere
Chr6A:Centromere
Chr5D:Centromere
Chr5B:Centromere
Chr5A:Centromere
Chr4D:Centromere
Chr4B:Centromere
Chr4A:Centromere
Chr3D:Centromere
Chr3B:Centromere
Chr3A:Centromere
Chr2D:Centromere
Chr2B:Centromere
Chr2A:Centromere
Chr1D:Centromere
Chr1B:Centromere
Chr1A:Centromere
Chromosome
0e+00
5e+06
1e+07
Position
SatDNA Family
TaeSat16−567

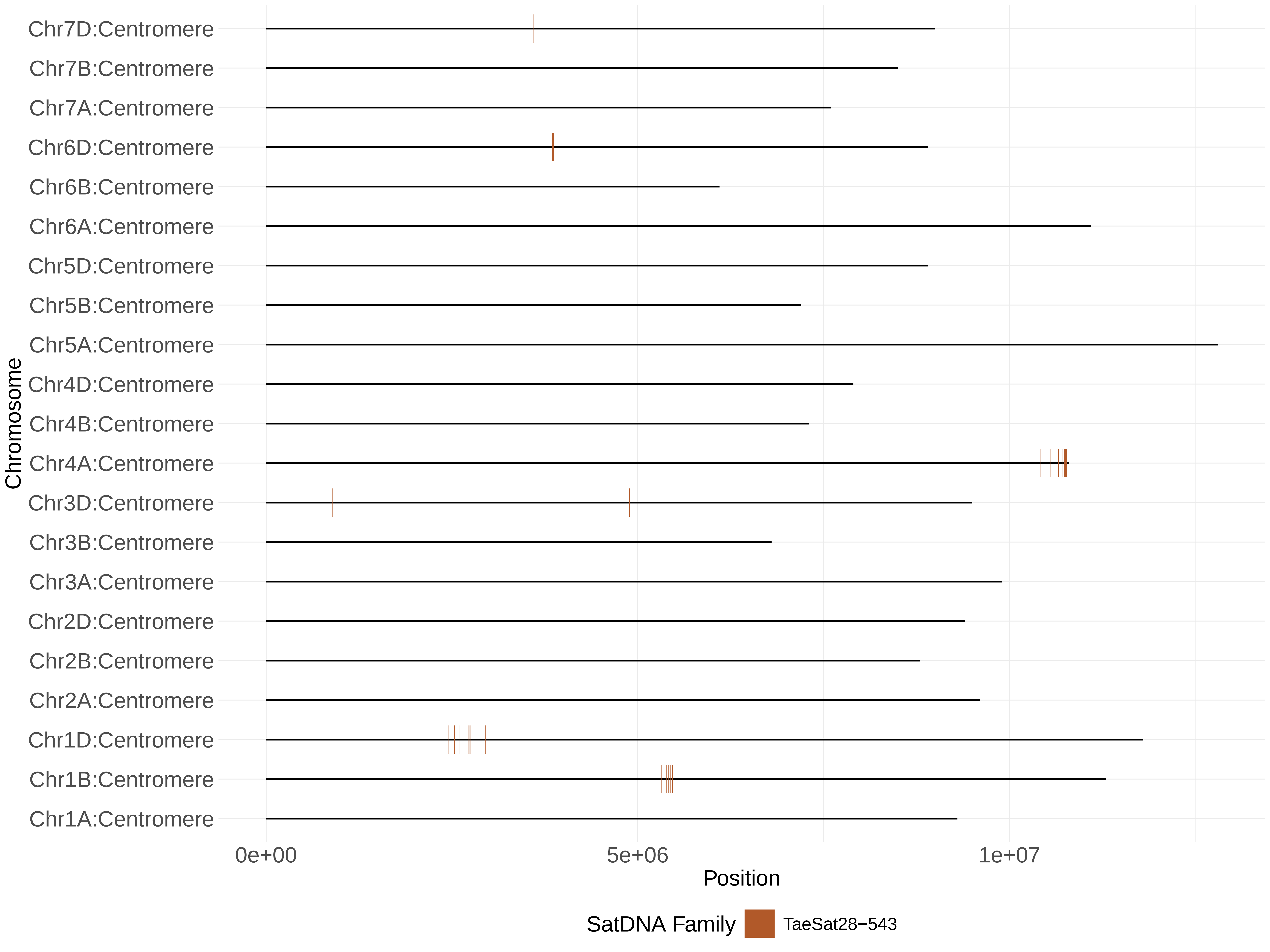

Chr7D:Centromere
Chr7B:Centromere
Chr7A:Centromere
Chr6D:Centromere
Chr6B:Centromere
Chr6A:Centromere
Chr5D:Centromere
Chr5B:Centromere
Chr5A:Centromere
Chr4D:Centromere
Chr4B:Centromere
Chr4A:Centromere
Chr3D:Centromere
Chr3B:Centromere
Chr3A:Centromere
Chr2D:Centromere
Chr2B:Centromere
Chr2A:Centromere
Chr1D:Centromere
Chr1B:Centromere
Chr1A:Centromere
Chromosome
0e+00
5e+06
1e+07
Position
SatDNA Family
TaeSat28−543
